# Phosphoproteomics identifies dual-site phosphorylation in an extended basophilic motif regulating FILIP1-mediated degradation of filamin-C

**DOI:** 10.1038/s42003-020-0982-5

**Published:** 2020-05-22

**Authors:** Lena Reimann, Anja N. Schwäble, Anna L. Fricke, Wignand W. D. Mühlhäuser, Yvonne Leber, Keerthika Lohanadan, Martin G. Puchinger, Sascha Schäuble, Erik Faessler, Heike Wiese, Christa Reichenbach, Bettina Knapp, Christian D. Peikert, Friedel Drepper, Udo Hahn, Clemens Kreutz, Peter F. M. van der Ven, Gerald Radziwill, Kristina Djinović-Carugo, Dieter O. Fürst, Bettina Warscheid

**Affiliations:** 1 Biochemistry and Functional Proteomics, Institute of Biology II, Faculty of Biology, University of Freiburg, 79104 Freiburg, Germany; 2 Department of Molecular Cell Biology, Institute for Cell Biology, University of Bonn, 53121 Bonn, Germany; 3 Department of Structural and Computational Biology, Max F. Perutz Laboratories, University of Vienna, A-1030 Vienna, Austria; 4 Jena University Language & Information Engineering (JULIE) Lab, Friedrich-Schiller-University Jena, 07743 Jena, Germany; 5 Systems Biology and Bioinformatics Unit, Leibniz Institute for Natural Product Research and Infection Biology, Hans Knöll Institute, Jena, Germany; 6 Institute of Medical Biometry and Statistics, Faculty of Medicine and Medical Center, University of Freiburg, 79104 Freiburg, Germany; 7 Signalling Research Centres BIOSS and CIBSS, University of Freiburg, Freiburg, Germany; 8 Present Address: Institute of Pharmacology and Toxicology, University of Ulm, 89081 Ulm, Germany; 9 Present Address: Bioinformatics Research & Development, BioNTech SE, 55131 Mainz, Germany

**Keywords:** Phosphorylation, Proteome, Mass spectrometry

## Abstract

The PI3K/Akt pathway promotes skeletal muscle growth and myogenic differentiation. Although its importance in skeletal muscle biology is well documented, many of its substrates remain to be identified. We here studied PI3K/Akt signaling in contracting skeletal muscle cells by quantitative phosphoproteomics. We identified the extended basophilic phosphosite motif RxRxxp[S/T]xxp[S/T] in various proteins including filamin-C (FLNc). Importantly, this extended motif, located in a unique insert in Ig-like domain 20 of FLNc, is doubly phosphorylated. The protein kinases responsible for this dual-site phosphorylation are Akt and PKCα. Proximity proteomics and interaction analysis identified filamin A-interacting protein 1 (FILIP1) as direct FLNc binding partner. FILIP1 binding induces filamin degradation, thereby negatively regulating its function. Here, dual-site phosphorylation of FLNc not only reduces FILIP1 binding, providing a mechanism to shield FLNc from FILIP1-mediated degradation, but also enables fast dynamics of FLNc necessary for its function as signaling adaptor in cross-striated muscle cells.

## Abstract (web summary)

Reimann, Schwäble et al. perform quantitative proteomics to study PI3K/Akt signaling in contracting myotubes. They identify a dual-site phosphorylation motif in the actin cross-linker and signaling adaptor filamin C, which regulates its degradation and mobility, suggesting the importance of dual phosphorylation for filamin C function in striated muscle cells.

## Introduction

Due to its importance in cellular physiology, the PI3K/Akt pathway has been widely studied in various cell types^1^. In skeletal muscle, PI3K/Akt signaling promotes the differentiation of proliferating myoblasts into multinuclear myotubes and muscle growth^2,3^. Pathway activation leads to phosphorylation of Akt at T308 by the kinase PDK1. Subsequently, Akt reaches its full activity by mTORC2-mediated phosphorylation at S473^4^. mTORC2 can also activate PKCα, resulting in a modulation of the actin cytoskeleton^5^. Fully activated Akt promotes protein synthesis and cell growth via mTORC1 and controls gene expression by phosphorylating Foxo3^6^.

The PI3K p110 alpha pathway, activated by IGF-1, directly alters the expression of a small number of genes important for sarcomere maturation and Z-disc alignment in cross-striated muscle cells^7,8^. Among the genes affected by PI3K are the Z-disc-associated components FLNc, synaptopodin 2 (Synpo2/myopodin) and LIM domain binding 3 (Ldb3/ZASP/cypher). More recently, we identified myofibrillar Z-discs as a hotspot of protein phosphorylation^9^, underscoring their central role in myocyte signaling^10^. In particular, FLNc and several of its binding partners including Synpo2, Ldb3, Bcl-2-associated athanogene 3 (Bag3) and Xin actin-binding repeat-containing protein 1 (Xirp1) were identified as multi-site phosphorylated proteins in contracting skeletal myotubes^9^.

Together with FLNa and FLNb, FLNc belongs to the filamin family of actin-crosslinking proteins. All three filamins function as structural and signaling scaffolds and are composed of an amino-terminal actin-binding domain and a rod of 24 immunoglobulin-like domains (d) which are connected by flexible hinge regions between d15 and d16 (hinge 1) and d23 and d24 (hinge 2)^11^. Filamins form homodimers via their carboxy-terminal d24, enabling them to effectively cross-link actin filaments^12,13^. FLNa and FLNb are almost ubiquitously expressed across tissues. In contrast, FLNc is predominantly expressed in cross-striated muscle, where it mainly localizes at Z-discs and only in small amounts at costameres in association with the dystrophin-associated glycoprotein complex^14,15^. Because of its dynamic shuttling between Z-discs and the sarcolemma or other cellular compartments, FLNc has been suggested to function as signaling adaptor rather than structural scaffold^15–17^. Notably, massive redistribution of FLNc was observed in myofibres of patients suffering from muscle diseases^18,19^. Due to its function as scaffold during Z-disc assembly, FLNc is crucial for the development of myofibrils^20^ and the repair of damaged myofibrils^17^. In humans, mutations in the gene encoding FLNc cause myopathies and cardiomyopathies^21–23^. However, despite its vital roles in sarcomere formation, maintenance, repair and signal integration, knowledge about its regulation is largely incomplete. Since we and others identified FLNc as phosphoprotein^9,24,25^, we propose that it is under control of different cytosolic kinases which precisely modulate its protein interactions, dynamics and functions by site-specific phosphorylations. We have shown that the hinge 2 region of FLNc is targeted by PKCα at distinct serines, which is important for its functional control^9^. A further site of regulation is d20, which directs FLNc to the Z-disc^26^. This domain is involved in multiple protein interactions^26^ and it is phosphorylated by Akt in response to insulin or growth factor stimulation^24^.

Here, we studied the IGF-1-PI3K/Akt pathway in contracting C2 mouse myotubes by quantitative phosphoproteomics. We identified an extended basophilic motif in various members of the PI3K/Akt/mTOR signaling network including FLNc, and established Akt and PKCα as the kinases responsible for dual-site phosphorylation of FLNc within the extended motif. We identified filamin-A-interacting protein 1 (FILIP1, first cloned in mouse as FILIP) as a direct binding partner of FLNc and uncovered that dual-site phosphorylation of FLNc modulates FILIP1 binding. Expression levels of FLNc and FILIP1, unlike FLNa, strongly increase during skeletal muscle cell differentiation and both are essential for the formation of functional sarcomeres. We here show that dual-site phosphorylation within the extended motif renders FLNc more resistant to FILIP1-promoted degradation. Moreover, these phosphorylations are required for fast dynamics and high mobility of FLNc and thus for its function as signaling adaptor in cross-striated muscle cells.

## Results

### Analysis of PI3K/Akt signaling in contracting myotubes

**Fig. 1:**
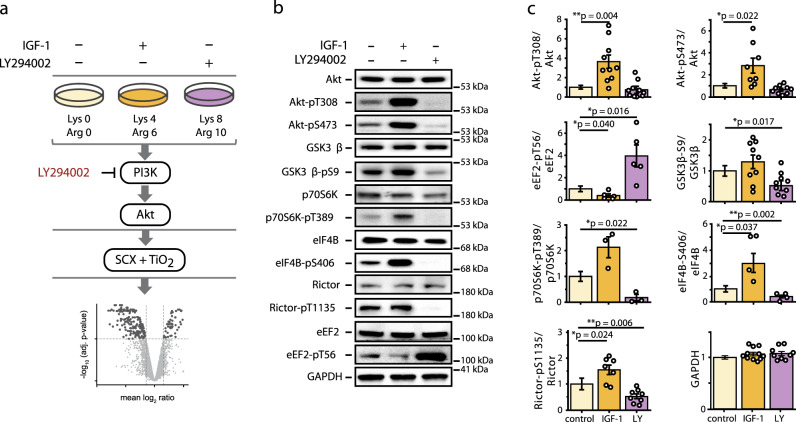
Experimental setup to analyze PI3K/Akt signaling in contracting C2 myotubes. **a** Contracting C2 myotubes, differentially labeled by stable isotopes using a triple stable isotope labeling with amino acids in cell culture (SILAC) approach and subjected to mild electrical pulse stimulation, were treated for 1 h with IGF-1 or LY294002 to stimulate or inhibit PI3K/Akt signaling as indicated. Cell lysates from triple SILAC experiments (*n* = 3 independent experiments) were individually subjected to SDS-PAGE and immunoblot analysis or mixed in equal amount for quantitative phosphoproteome analysis. Phosphopeptides were enriched using strong cation exchange chromatography and titanium dioxide chromatography (SCX-TiO_2_) and measured by LC-MS followed by computational data analysis. **b** Immunoblot analysis of PI3K/Akt/mTOR pathway activity using total and phospho-specific antibodies against established substrates of the canonical PI3K/Akt/mTOR pathway. **c** Quantification of immunoblot data from (**b**). Calculated signal intensities were normalized to the control and a two-tailed paired student’s *t*-test was performed. Error bars represent the SEM, *n* = 3-11 independent experiments.

To explore the PI3K/Akt-associated signaling network in contracting skeletal myotubes, we analyzed changes in the phosphoproteome following stimulation of cells with IGF-1 or inhibition of PI3K/mTOR activity using LY294002 (LY) (Fig. 1a). To promote the formation of fully differentiated contracting skeletal myotubes with mature Z-discs, myocytes were subjected to mild electrical pulse simulation (EPS)^9^. EPS resulted in a mild activation of PI3K/Akt/mTOR signaling, which was reduced to basal level when myotubes were starved (Supplementary Figs. 1a and 2). IGF-1 treatment resulted in a strong increase in Akt activity, which was abrogated by LY as monitored by the levels of Akt-pT308/-pS473 and GSK3β-pS9 (Fig. 1b, c and Supplementary Fig. 2). Levels of p70S6K-pT389 and eIF4B-pS406, used as readouts for mTORC1, and Rictor-pT1135 and Akt-pS473 for mTORC2, were increased in response to IGF-1 and decreased by LY. In agreement with these data, phosphorylation levels of eukaryotic elongation factor 2 (eEF2) at T56, a substrate of eEF2 kinase downstream of mTORC1, were reversed (Fig. 1b, c and Supplementary Fig. 2).

Quantitative phosphoproteomic analysis resulted in 7,202 protein identifications, including 3,490 phosphoproteins with 16,633 identified and 11,493 (69%) localized phosphosites (Fig. 1a, Supplementary Fig. 1b–e, and Supplementary Data 1 and 2). 73% (8,441) of all localized phosphosites were quantified in at least two replicates (Supplementary Fig. 1f–i). Most phosphopeptides did not change in abundance and treatments had no effect on protein abundance (Supplementary Fig. 1j–l).

### Identification of an extended basophilic kinase motif

**Fig. 2:**
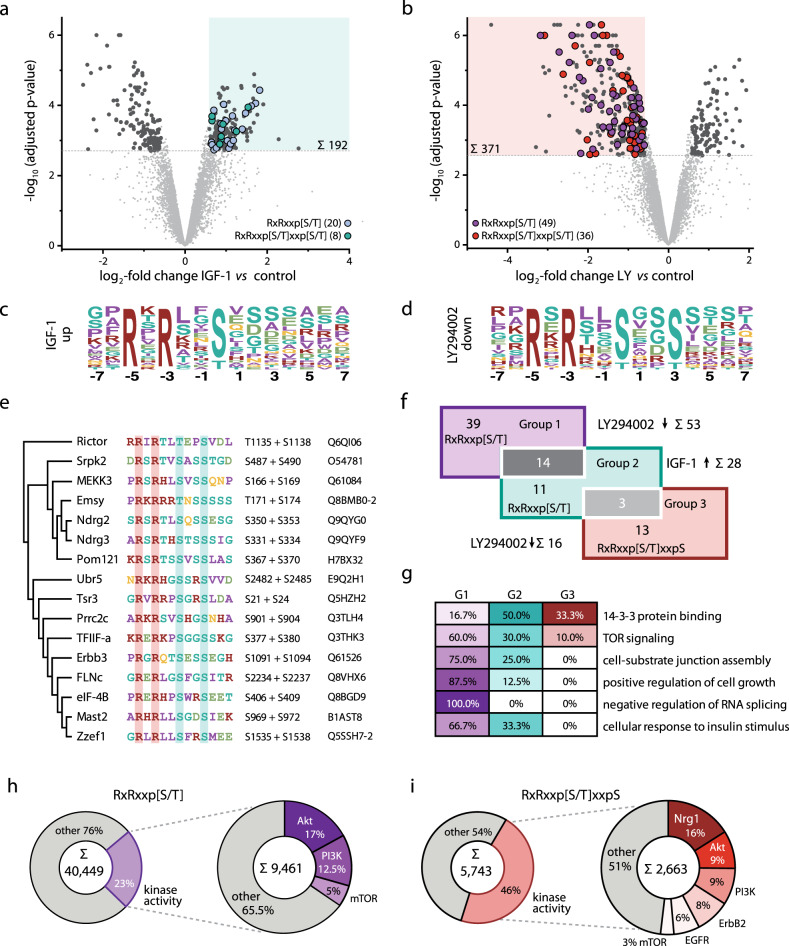
Identification of an extended basophilic motif by quantitative phosphoproteomics. **a**, **b** Volcano plots of quantified phosphopeptides with localized phosphosites from fully differentiated contracting skeletal myotubes following treatment with IGF-1 (left) or LY294002 (right). Log_2_-transformed mean SILAC ratios (control/treatment) were plotted against −log_10_ adjusted *p* values. Phosphopeptides with a minimum fold change of 1.5 and an adjusted *p* value lower than 0.05 (*n* = 3 independent experiments; two-tailed moderated student’s *t*-test) are shown as dark gray circles. Among these, phosphopeptides are labeled according to their phosphorylation sequence motif as indicated and regions of interest are highlighted in each plot. **c**, **d** Motif-X analysis of regulated phosphopeptides. The basophilic motif RxRxxS (**c**) was 30-fold enriched upon IGF-1 treatment, whereas the extended sequence motif RxRxxSxxS (**d**) was found to be 123-fold enriched following LY294002 treatment. x, any amino acid. **e** Clustal Omega analysis of the 16 amino acid sequence window from the proteins comprising regulated peptides with the extended basophilic RxRxxp[S/T]xxpS. **f** Overlap of unique phosphopeptides comprising the classical basophilic motif RxRxxp[S/T] (Group 1, LY294002 downregulated), RxRxxp[S/T] (Group 2, IGF-1 upregulated), or the extended basophilic motif RxRxxp[S/T]xxpS (Group 3, LY294002 downregulated). **g** Combined GO enrichment analysis of phosphopeptides of group 1, 2 and 3 (G1–3) shown in (**e**). Shown are overrepresented pathways with a Benjamini-Hochberg corrected *p* value ≤ 0.05. **h**, **i** Text mining results for interaction partners of proteins comprising the RxRxxp[S/T] (**h**) or the extended RxRxxp[S/T]xxpS motif (**i**). Analysis resulted in 40,449 (**h**) and 5,743 matches (**i**) of which 9,461 (23%) and 2,663 (46%) were annotated with the term “kinase activity”.

Phosphoproteome data were visualized in Volcano plots showing that 192/167 and 123/371 phosphopeptides were up/downregulated in response to IGF-1 and inhibition of PI3K (LY), respectively (Fig. 2a, b, Supplementary Fig. 3a, b, and Supplementary Data 2). The basophilic motif RxRxxS was enriched (31-fold) in IGF-1 upregulated phosphopeptides, whereas the extended motif RxRxxSxxS was strongly overrepresented (123-fold) in LY downregulated peptides (Fig. 2c, d and Supplementary Data 3). Manual annotation of IGF-1 upregulated phosphopeptides by verifying sequence windows comprising pS/pT resulted in the identification of the classical motif RxRxxp[S/T] in 20 regulated phosphopeptides, while 8 additional peptides contained the extended motif RxRxxp[S/T]xxp[S/T] (Fig. 2a). In response to LY, the classical and extended motifs were identified in 49 and 36 downregulated phosphopeptides (Fig. 2b). Altogether, 16 proteins comprised the extended motif, with S/T in the central and +3 position being concurrently phosphorylated (Fig. 2e and Supplementary Data 3).

Cross-comparison of regulated phosphopetides with these basophilic motifs showed only minor overlaps between groups (Fig. 2f and Supplementary Data 3). GO enrichment analysis revealed that G1 proteins are predominantly involved in negative regulation of RNA splicing, induction of cell growth or TOR signaling, and G2 and G3 proteins in 14–3–3 binding (Fig. 2g and Supplementary Data 4). Furthermore, many proteins in G1 and G2 function in insulin response and assembly of cell-to-substrate junctions. In contrast, for IGF-1 down- and LY upregulated phosphopeptides, the proline-directed motif pSxxxpSP was overrepresented in proteins functioning in actomyosin structure organization or transcriptional processes (Supplementary Fig. 3a–f). STRING network analysis highlights the prevalence of the classical and extended basophilic motif in proteins of the PI3K/Akt/mTOR network, whereas proteins with functions in gene expression comprised proline-directed motifs (Supplementary Fig. 3g). We further employed a text mining pipeline to reveal PI3K/Akt/mTOR network-associated proteins comprising the basophilic motifs. Using protein lists of G1–G3, text mining revealed 40,449 and 5,743 interaction events for the classical and extended motif, respectively (Fig. 2h, i). Filtering these results for events associated with the GO term ‘kinase activity’ showed that Akt and PI3K are most prominent for proteins with the classical motif (Fig. 2h and Supplementary Data 5). For the extended motif, nearly half of the interactions are associated with the term ‘kinase activity’, with neuregulin 1 (Nrg1)/Erb-B2 receptor tyrosine kinase 2 (ErbB2) and Akt/PI3K being prominent events (Fig. 2i and Supplementary Data 5).

### Akt targets substrates within the extended basophilic motif

**Fig. 3:**
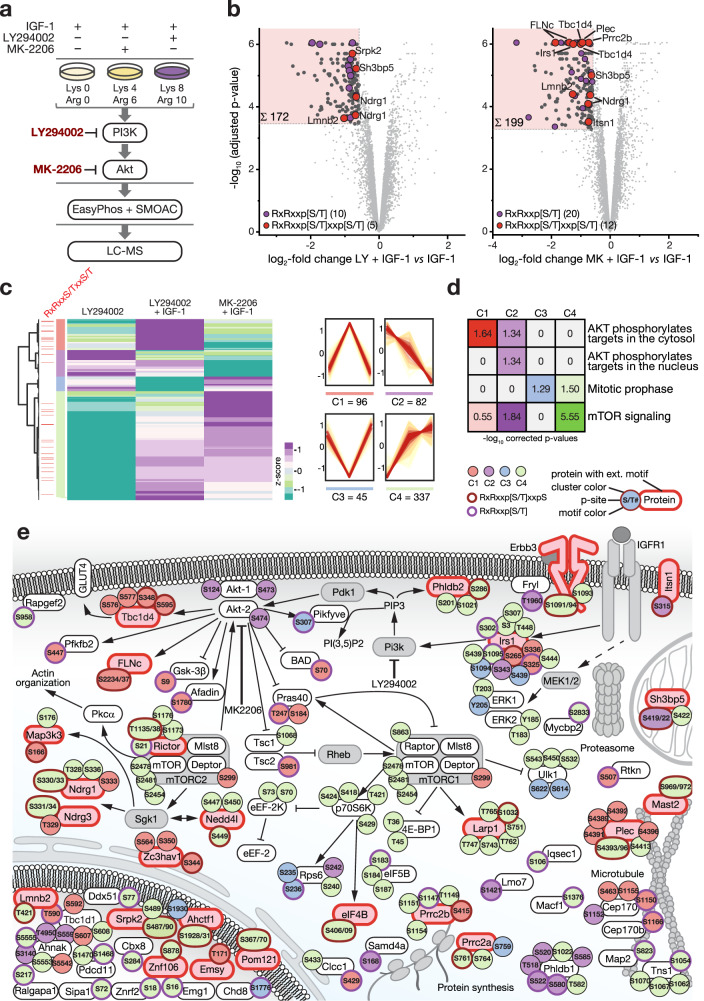
FLNc is a component of the PI3K/Akt/mTOR signaling network and differentially phosphorylated within the extended motif RxRxxpSxxpS. **a** Contracting C2 myotubes differentially labeled using SILAC amino acids were treated for 30 min with IGF-1 or IGF-1 together with MK-2206 (MK) or LY294002 (LY) to stimulate or inhibit PI3K/Akt signaling as indicated. Cell lysates were mixed in equal amount for phosphopeptide enrichment employing the EasyPhos method in combination with sequential enrichment using metal oxide affinity chromatography (SMOAC) and LC-MS analysis. **b** Volcano plots of phosphopeptides with localized phosphosites quantified in skeletal myotubes treated with IGF-1 + LY (left) or IGF-1 + MK (right) in comparison to IGF-1. Log_2_-transformed mean SILAC ratios were plotted against -log_10_ adjusted *p* values. Phosphopeptides with a minimum fold change of 1.5 and a FDR lower than 0.01 (*n* = 6 independent experiments; two-tailed moderated student’s *t*-test) are shown as dark gray circles or color-coded according to their phosphorylation sequence motif as indicated. Regions of interest are highlighted in each plot. **c**. Hierarchical cluster analysis of cluster 2 shown in Supplementary Fig. 4f and the respective phosphopeptide profiles in each subcluster. Phosphopeptides with dual-site phosphorylation in the extended basophilic motif are indicated (light red lines, left side). **d** Reactome^29^ analysis of phosphopeptides of the cluster 1–4 depicted in (**c**). Shown are overrepresented pathways with a corrected *p* value ≤ 0.05. **e** Excerpt from the canonical IGF-1 activated signaling pathway, comprising the downstream signaling branch of the PI3K/Akt signaling cascade. Interactions were curated from the literature and the KEGG database. Phosphorylation sites are color-coded according to their cluster affiliation shown in (**c**) and the presence of the short or extended basophilic motif. Proteins comprising the extended basophilic motif are further highlighted in red; proteins not identified are shown in light gray. Proteins are represented by their gene name or UniProt shortname.

To identify proteins comprising the extended motif as substrates of Akt, we designed a differential myotube phosphoproteome study using IGF-1 in combination with LY or the Akt inhibitor MK-2206 (MK) (Fig. 3a and Supplementary Figs. 4a and 2). For direct comparison, LY/MK and IGF-1/LY data were searched together, resulting in 10,326 localized and reproducibly quantified phosphosites in the LY/MK dataset (Supplementary Fig. 4b, c and Supplementary Data 6). Following MK treatment, a considerably higher number of downregulated phosphopeptides with the short or extended motif was found (Fig. 3b). Kinase-substrate enrichment analysis (KSEA)^27^ showed that LY mainly decreased p70S6K and mTOR activity, whereas MK predominantly diminished Akt activity and PKC was mostly reduced when directly comparing the effects of MK and LY (Supplementary Fig. 4d and Supplementary Data 6).

To distinguish between substrates of Akt and p70S6K downstream of mTOR (both phosphorylate the RxRxxS/T motif^28^), we performed sequential hierarchical cluster analysis and Reactome^29^ analysis (Supplementary Data 7). We first identified a main cluster of 560 phosphopeptides downregulated in response to LY or MK compared to IGF-1 (Supplementary Fig. 4e). Further clustering of these peptides resulted in four clusters with an over-representation of cytosolic and nuclear Akt substrates in cluster 1 and 2 (Fig. 3c, d). Distinct from Akt targets, mTOR/p70S6K substrates were strongly overrepresented in the cluster 4. Proteins phosphorylated in the extended motif were mainly present in cluster 1 (11 phosphopeptides, 8 proteins) and cluster 4 (18 phosphopeptides, 16 proteins), suggesting that these are direct substrates of Akt and p70S6K, respectively.

### FLNc is phosphorylated within the extended basophilic motif

We mapped our quantitative phosphoproteome data to the canonical PI3K/Akt/mTOR pathway (Fig. 3e). Rictor, a central component of mTORC2, was found to be differentially phosphorylated at both sites (pT1135, pS1138) of the extended motif. In addition, altered phosphorylation within the extended motif were found for downstream targets of mTORC1 (eIF4B, Larp1) and mTORC2 (Map3k3, Ndrg1, Nrdg3, Nedd4l), plasma membrane proteins (Erbb3, Itsn1, Phldb2), insulin-responsive factors (Irs1, Tbc1d4), nuclear proteins (Ahctf1, Emsy, Lmnb2, Pom121, Srpk2, Znf106), microtubule-associated proteins (Mast2, Plec), the mitochondria-associated protein Sh3bp5, and putative RNA-binding proteins (Prrc2a, Prrc2b, Zc3hav1). We also identified the muscle-specific protein FLNc to be differentially phosphorylated in this motif. Among the 17 phosphosites of FLNc identified, the two abundant and regulated sites, pS2234 and pS2237, are located in the extended motif, which is 100% conserved among mammals (Supplementary Figs. 4f-g and 5, Supplementary Data 2 and 6). Quantitative immunoblots confirmed a significant decrease of pS2234 following LY treatment (Supplementary Figs. 4h and 2). Furthermore, while levels of pS2234 and pS2234/pS2237 were strongly reduced following inhibition of PI3K/Akt signaling and increased upon activation, levels were reversed for pS2237 (Supplementary Fig. 4i). Thus, our MS data suggest that concurrent phosphorylation of FLNc at S2234 and S2237 prevails in myotubes with pS2234 being the main site regulated by PI3K/Akt.

### Akt and PKC target the unique insert of FLNc domain 20

Our finding that mouse FLNc (mFLNc) is concurrently phosphorylated at S2234 and S2237 prompted us to examine the responsible kinases. In human FLNc (hFLNc), the two orthologous sites, S2233 and S2236, were found to be phosphorylated in vivo^30^. In silico kinase prediction indicated that the first serine is a substrate of Akt, whereas the second serine (+3 position) is a substrate of PKC/PKCα (Supplementary Fig. 6a). Using the inhibitor MK, we provide evidence that mFLNc-S2234 is a direct target of Akt in skeletal myotubes (Supplementary Figs. 6b and 2).

**Fig. 4:**
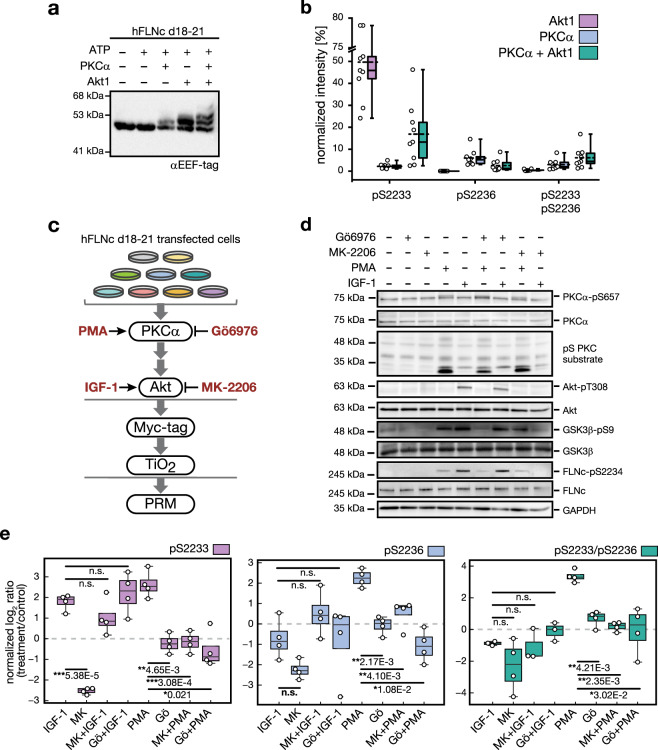
Phosphorylations within the extended motif located in the insert of hFLNc domain 20 are mediated by Akt and PKCα. **a** In vitro kinase assay coupled to phosphorylation-dependent mobility shift analysis. Reactions were performed using recombinant hFLNc d18–21 in the presence (+) or absence (−) of ATP and the two basophilic kinases Akt and PKCα. Immunoblot analysis was performed with an antibody directed against the EEF-tag that was fused to the carboxy-terminus of hFLNc d18–21. A specific phosphorylation-dependent mobility shift was detected following incubation with PKCα. Akt-mediated phosphorylation led to band shift of approximately 50% of the protein. Incubation with both kinases resulted in two shifted bands, indicating dual-site phosphorylation of the protein. d, domain. **b** In vitro kinase assay coupled to quantitative MS analysis for site determination. Reactions were performed as described in (**a**). MS data for each kinase were quantified using Skyline. Intensities of phosphopeptides distinctive for a specific phosphorylation site in hFLNc d18–21 were added up per experiment and represented as normalized mean ± SEM (*n* = 3 independent experiments, each with 3 technical replicates). **c** Cell-based kinase assay. PKC activator PMA, PKCα inhibitor Gö6976 and Akt activator IGF-1 and Akt inhibitor MK-2206 were used to activate or block signaling pathways in C2 cells. For targeted MS analysis, phosphopeptides were enriched by Myc-tag and TiO_2_-based enrichment. **d** Immunoblot analysis for monitoring the activity levels of PKCα and Akt in C2 cells following pharmacologic interventions as indicated in (**c**). Specific antibodies were used to detect total protein amounts and phosphoisoforms. GSK3β-pS9 is a direct substrate of Akt and pS PKC substrate is an antibody directed against PKC substrates. GAPDH was used as loading control. **e** Targeted MS data showing changes in phosphorylation of hFLNc at S2233 and S2236. For parallel reaction monitoring, the phosphopeptides LGpSFGSITR, LGSFGpSITR, LGpSFGpSITR, ERLGpSFGSITR and ERLGpSFGpSITR were selected. MS data were quantified using Skyline and normalized to an internal phosphopeptide standard and a two-tailed paired student’s *t*-test was performed. Shown are the normalized mean log_2_ ratios (treatment/control) ± SEM, *n* = 4 independent experiments.

To investigate phosphorylation of hFLNc by Akt and PKCα, we performed in vitro kinase assays using recombinant hFLNc d18–21 (Supplementary Fig. 7a). PhosTag PAGE analysis revealed that both kinases led to phosphorylation-dependent electrophoretic mobility shifts (Fig. 4a). In vitro kinase assays combined with LC-MS confirmed hFLNc-S2233 as a substrate of Akt, whereas PKCα preferentially targeted S2236. Accordingly, incubation with both kinases led to an increase of the doubly phosphorylated form of hFLNc (Fig. 4a, b and Supplementary Data 8).

To validate these findings in myocytes, we performed cell-based kinase assays with C2 cells expressing Myc-tagged hFLNc d18–21 and pharmacologic interventions using IGF-1 and PMA for stimulation or Gö6976 and MK for inhibition, or a combination of each of the two inhibitors with IGF-1 or PMA followed by parallel reaction monitoring (PRM) (Fig. 4c). Immunoblot analysis showed that IGF-1 increased Akt activity, which was diminished by MK but not Gö6976 (Fig. 4d and Supplementary Fig. 2). PMA stimulated PKC whose activity was blocked by Gö6976. Since Gö6976 is a non-competitive inhibitor^31^, PKCα-pS657 levels remained constant following inhibition but were strongly increased with PMA. Stimulation of PKCα by PMA also induced Akt activity as monitored by GSK3β-pS9, which was blocked by MK.

PRM showed that levels of FLNc-pS2233, -pS2236 and -pS2233/pS2236 were strongly increased with PMA-induced high activity of PKCα and Akt (Fig. 4e and Supplementary Data 9). In contrast, IGF-1 led to an increase of pS2233 only, which was strongly decreased by MK. pS2236 was significantly reduced upon PKCα inhibition by Gö6976 alone or in combination with PMA. Similarly, pS2233/pS2236 levels were strongly reduced when PKCα was blocked. Since S2233A or S2233D point mutations in hFLNc d18–21 did not alter phosphorylation of S2236 (Supplementary Fig. 6c–e), a priming phosphorylation at S2233 appears not to be required.

In sum, our data show that Akt and PKCα are the two basophilic kinases responsible for phosphorylating FLNc within the extended motif RERLGSFGS located in its unique insert in d20.

### FILIP1 is a binding partner of FLNc in skeletal myotubes

**Fig. 5:**
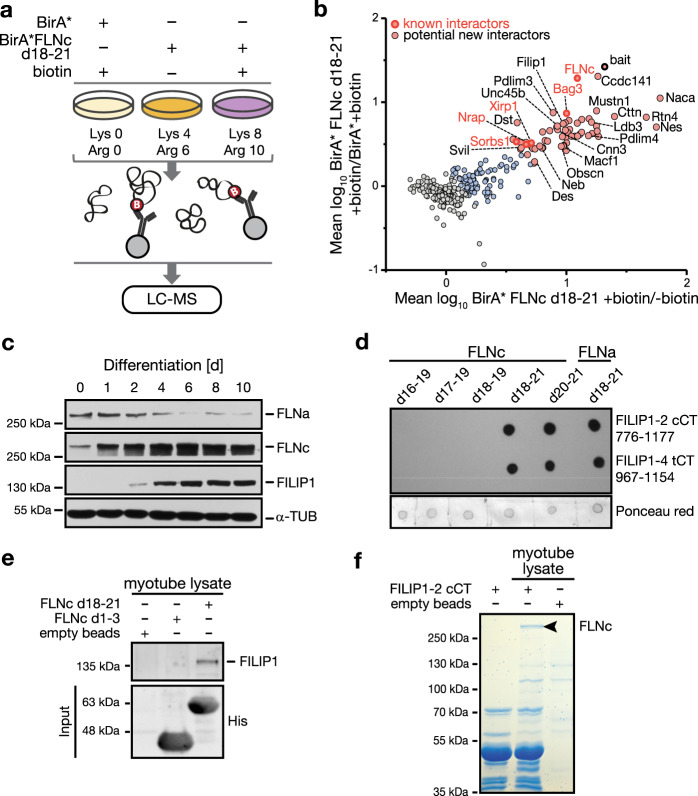
Identification of FILIP1 as binding partner of hFLNc d18–21 in skeletal myocytes. **a** SILAC-based proximity proteomic approach. Differentially stable isotope labeled contracting C2 myotubes (*n* = 3 independent experiments) transiently expressing the promiscuous biotin ligase BirA* or BirA*hFLNc d18–21 were incubated with biotin as indicated. Cell lysates were mixed, biotinylated proteins were enriched via streptavidin and tryptic digests thereof were quantitatively analyzed by LC-MS. **b** Scatterplot of proteins grouped in clusters 1–3 shown in Supplementary Fig. 8a. Components of cluster 1 (pink) were enriched in relation to both controls (BirA* + biotin; BirA*hFLNc d18–21—biotin). Shown are the mean log_10_ SILAC ratios from 3 independent experiments. Proteins are represented by their gene name. **c** Immunoblot analysis of the levels of FLNa, FLNc and FILIP1 during differentiation of C2C12 cells to myotubes. α-Tubulin (α-TUB) was used as loading control. **d** Dot-blot overlays showing binding of different EEF-tagged fragments of FLNc and FLNa to the complete carboxy-terminus (cCT) of FILIP1-2 (amino acids 776–1177) and the truncated carboxy-terminus (tCT) FILIP1-4 (amino acids 967–1154) immobilized on nitrocellulose. Staining with anti-EEF-tag antibody shows binding of the filamin fragments. Staining with Ponceau red indicates loading of equal protein amounts. **e** Pull-down experiments confirming the binding of endogenous FILIP1 from skeletal myotube lysates to His_6_-FLNc d18–21 bound to Ni^2+^-NTA agarose beads by immunoblot analysis. No FILIP1 binding was observed for empty beads or His_6_-FLNc d1–3. **f** Pull-down experiments verifying the binding of endogenous FLNc from skeletal myotube lysates to the carboxy-terminus of FILIP1-2 bound to Ni^2+^-NTA agarose beads. Bound proteins were analyzed by SDS-PAGE and Coomassie staining. This revealed a specific protein band not present in control lanes at an approximate molecular mass of 290 kDa, which was identified as FLNc by LC-MS analysis shown in Supplementary Fig. 8h.

FLNc interacts with various proteins via its carboxy-terminal portion including d20^15,25,26,32,33^ and phosphorylation may provide a mechanism to modulate these interactions. To analyze the interactome of the FLNc region around d20 in contracting myotubes, we performed SILAC and in vivo proximity labeling using the promiscuous biotin ligase BirA*^34^ fused to hFLNc d18–21 (Fig. 5a and Supplementary Fig. 7b). Through hierarchical clustering of SILAC data, we established the hFLNc d18–21 interactome (cluster 1), with an over-representation of Z-disc-associated proteins including endogenous mFLNc, Bag3, Ldb3 and Xirp1 (Fig. 5b, Supplementary Fig. 8a, b and Supplementary Data 10 and 11). Bag3, Xirp1, Nrap and Sorbs1 are known direct binding partners of FLNc d18–21^32,35–37^. Other putative binding partners of FLNc are involved in muscle development and contraction (Naca, Mustn1, Unc45b, Cnn3) or contribute to the structural integrity of sarcomeres (Des, Neb), which highlights the central role of FLNc in skeletal muscle differentiation and maintenance. Further candidates are involved in cytoskeletal organization and cell migration (Ccdc141, Cttn, Rtn4, Nes, Pdlim3, Pdlim4, Dst, Macf1 and Svil). Interestingly, FILIP1, which binds FLNa in neural cells and targets it for degradation^38^, was among the potential interaction partners of hFLNc d18–21 in skeletal myotubes.

An interaction of FILIP1 with FLNc has not been reported. To address the role of the interaction of FILIP1 with the filamins in muscle cells, we analyzed its expression in differentiating cultured myotubes and compared it with the expression profiles of FLNa and FLNc. FLNa levels steadily decreased during differentiation, whereas FLNc levels increased simultaneously (Fig. 5c). FILIP1 was not detected in proliferating myoblasts and early differentiating myotubes, when FLNa levels were highest. Instead, its expression was only revealed two days after induction of differentiation when FLNa starts to be replaced by FLNc. For immunoblot analysis, we used an affinity-purified antiserum raised against the carboxy-terminus (CT) of a new isoform of FILIP1, termed FILIP1–4, lacking exon 6 in its mRNA due to alternative splicing (Supplementary Fig. 8c, d). Specificity of the antiserum was verified by blocking its reactivity by addition of the T7-tagged antigen (Supplementary Fig. 8e). As a result, the signal observed on Western blots prepared from extracts of mouse skeletal muscle and cultured myotubes was completely blocked by the antigen. Interestingly, a new signal appeared in the high molecular mass region above 250 kDa. Incubation with anti-T7-tag antibody confirmed the presence of the recombinant antigen at this position. We interpreted these data as binding of the antigen/antibody complex to a ligand of FILIP1. Incubation with anti-FLNc antiserum showed the presence of FLNc at this position (Supplementary Fig. 8e).

Yeast two-hybrid analyses indicated that FLNc fragments d17–19, d18–21 and d20–21 interact with FILIP1-4 CT, whereas only a weak or no interaction was observed for FLNc d16–19 and d18–19 (Supplementary Fig. 8f). Dot-blot overlay assays confirmed strong binding of this FILIP1-4 fragment to FLNc d20-21 and d18–21 (Fig. 5d, left panel). FLNa d18–21 also bound FILIP1–4 CT (Fig. 5d, right panel). We obtained identical results for FILIP1-2 CT, indicating that the interaction is not isoform-specific and that FILIP1 binds via its carboxy-terminal region preferentially, but not only, to FLNc d20-d21.

To verify this domain-specific binding of FILIP1, we recombinantly expressed His_6_-tagged FLNc d1–3 and d18–21 (Supplementary Fig. 7a, c), coupled them to Ni^2+^-NTA beads and performed a pull-down experiment with skeletal myotube lysates. Using FILIP1 antibody, we found that FILIP1 binds to FLNc d18–21 and not FLNc d1–3 (Fig. 5e), which was confirmed by LC-MS (Supplementary Fig. 8g and Supplementary Data 12). Finally, in a reversed pull-down experiment using His_6_-tagged FILIP1-2 CT and myotube lysate, we confirmed the binding of endogenous full-length FLNc to the carboxy-terminus of FILIP1 by SDS-PAGE and LC-MS analysis (Fig. 5f, Supplementary Fig. 8h, and Supplementary Data 13).

### Phosphorylation regulates FILIP1 binding and FLNc dynamics

Since FILIP1 directly binds FLNc d18–21, we asked whether phosphorylations within the extended basophilic motif in d20 affect this interaction. We performed co-immunoprecipitation and pull-down experiments with recombinant hFLNc d18–21 and phosphosite mutants, while hFLNc d1–3 or hFLNc d22–24 served as negative controls (Supplementary Fig. 7a-d). In hFLNc d18–21, we mutated either S2233 (AS, DS) or S2236 (SA, SD), or both (AA, DD) to aspartate (D) or alanine (A) to mimic constitutively phosphorylated or non-phosphorylated variants of hFLNc. For pull-down assays, GST-FILIP1 CT (Supplementary Fig. 7e) was incubated with the different His_6_-tagged hFLNc d18–21 variants and bound proteins were analyzed by immunoblotting (Supplementary Fig. 9a). Quantification revealed significantly less efficient binding of FILIP1 to the DD mutant in comparison to wild-type hFLNc and its other variants (Supplementary Fig. 9b).

**Fig. 6:**
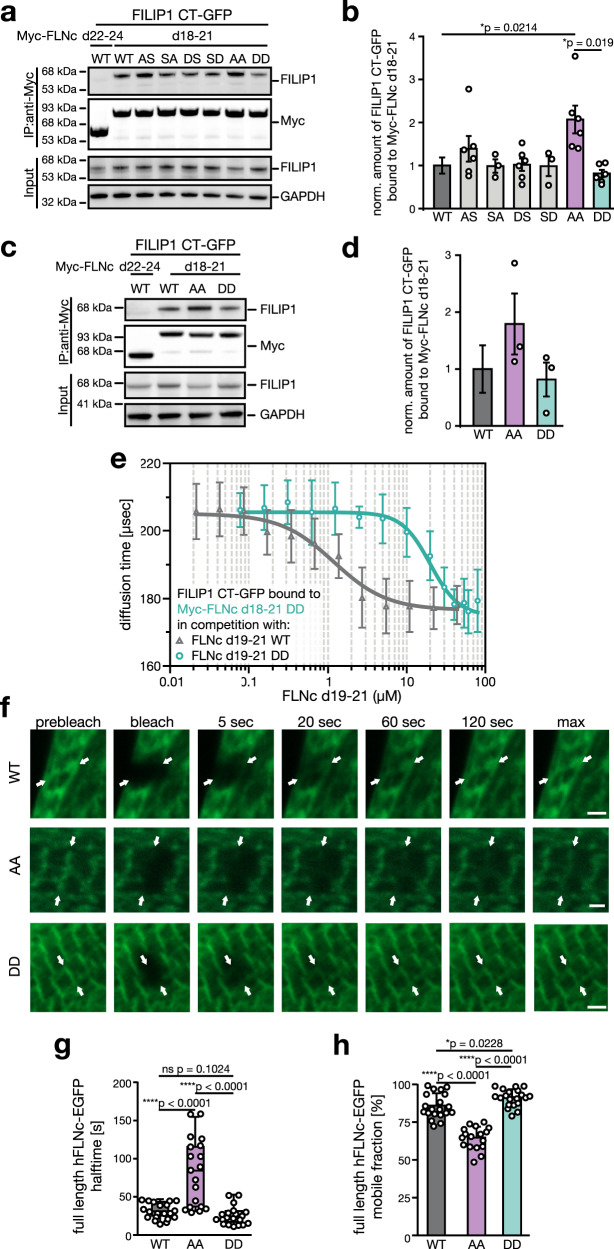
Dual-site phosphorylation at S2233/S2236 modulates binding of hFLNc to FILIP1 and increases hFLNc dynamics and mobility. **a** Co-immunopurification experiments in HEK293 cells transiently expressing FILIP1 CT-GFP and hFLNc d18–21 or single and double phosphosite mutants as indicated. FLNc d22–24, negative control. **b** Quantification of immunoblot data shown in (**a**) normalized to FLNc d18–21. SEM was calculated and a two-tailed student’s *t*-test was performed (*n* = 3–6 independent experiments). **c** Co-immunopurification experiments in C2 cells transiently expressing FILIP1 CT-GFP and hFLNc d18–21 or double phosphosite mutants as indicated. **d** Quantification of immunoblot data shown in (**c**) normalized to hFLNc d18–21. SEM was calculated and a two-tailed student’s *t*-test was performed (*n* = 3 independent experiments). **e** Fluorescence correlation spectroscopy analysis of co-expressed wild-type FILIP1 CT-GFP and Myc-hFLNc d18–21 DD mutant in competition with recombinantly expressed wildtype hFLNc d19–21 (*K*_d_ = 1.17 + 0.28 μM) and hFLNc d19–21 DD mutant (*K*_d_ = 20.03 + 2.33 μM), respectively. *n* = 3 independent experiments. **f** Immortalized mouse myoblasts transiently expressing EGFP-tagged full-length wild-type hFLNc (WT) or S2233/22236 phosphosite mutants (AA, DD) were differentiated for 6 days. Shown are representative FRAP experiments photographed before bleaching (prebleach), immediately (bleach) or 5, 20, 60 and 120 s after bleaching, and after full recovery (max). Arrows indicate the bleached Z-disc. Scale bar: 2 µm. **g** Quantification of FRAP data shown in (**a**). Statistical data are depicted in box and whisker plots with median halftimes of 33 (WT), 82 (AA) and 27 s (DD). Each median halftime is shown as a line surrounded by a box, which represents the interquartile range comprising the median 25% of the data. Whiskers extend at most two standard deviations from the median. Unpaired *t*-test with Welch correction was performed for *n* = 19–20 independent experiments. **h** Percentage of mobile fractions calculated from FRAP experiments in (**a**). The AA variant shows a significantly decreased mobility (65%) compared to WT FLNc (86%). The DD variant (91%) is significantly more mobile than WT FLNc. Shown is the mean ± S.D. Unpaired *t*-test with Welch correction was performed for *n* = 19–20 independent experiments. hFLNc d22–24 negative control; WT wild-type.

To verify this finding in human cells, we co-expressed FILIP1 CT-GFP (Supplementary Fig. 7e) with hFLNc d18–21 WT, the respective phosphosite mutants or Myc-tagged hFLNc d22–24 as control in HEK293 cells and performed co-immunoprecipitation experiments using anti-Myc antibody (Fig. 6a and Supplementary Fig. 2). Quantitative immunoblot analysis revealed a significant increase in FILIP1 binding to the AA mutant in comparison to both the wild-type and the DD mutant of hFLNc d18–21 (Fig. 6b). Since the difference in FILIP1 binding is highest for the obligatory non-phosphorylated AA mutant, our data indicate that also in HEK293 cells wild-type hFLNc d18–21 is largely phosphorylated, which is supported by MS data (Supplementary Fig. 9c). Analysis of this interaction in cultured mouse skeletal muscle cells confirmed preferred binding of FILIP1 to the AA mutant (Fig. 6c, d and Supplementary Fig. 2). We thus concluded that a major portion of FLNc is phosphorylated at both serines within the extended basophilic motif.

To determine the dissociation constant of this phosphorylation-dependent protein interaction, we used fluorescence correlation spectroscopy (FCS) using lysates of HEK293 co-expressing FILIP1 CT-GFP and Myc-hFLNc d18–21 or the phosphomimetic mutant (DD) (Supplementary Fig. 7b, e). For competition assays, recombinant hFLNc d19–21 or the respective DD mutant (Supplementary Fig. 7f) was added to the lysate. Non-phosphorylated hFLNc d19–21 was able to compete with Myc-hFLNc d18–21 DD bound to FILIP1 CT-GFP, whereas the phosphomimetic mutations significantly reduced the ability to compete (Fig. 6e). Thus, the non-phosphorylated form of FLNc binds FILIP1 more strongly, as reflected by an equilibrium dissociation constant (K_D_) of 1.17 + 0.28 μM determined for hFLNc d19–21 and 20.03 + 2.33 μM for its phosphomimicking variant. This finding was confirmed by the observation that hFLNc d19–21 DD mutant did not dissociate the interaction of FILIP1 CT-GFP and Myc-hFLNc d18–21 (Supplementary Fig. 9d).

FLNc is a highly dynamic and extremely mobile protein, two features that are of critical importance for its vital role in sarcomere assembly, maintenance and repair^17^. Obviously, these properties indicate a requirement for tight regulation of FLNc interactions and turnover in vivo. We performed fluorescence recovery after photobleaching (FRAP) experiments to assess whether phosphorylations within the extended motif modulate the dynamics and mobility of FLNc in living cells. FRAP experiments were performed in cultured mouse myotubes expressing full-length wild-type FLNc-EGFP, or the respective AA and DD variants (Supplementary Fig. 7h). Localization of the AA mutant was more strictly restricted to the Z-disc, whereas wild-type FLNc and the phosphomimicking DD mutant showed in addition a more diffuse distribution (Fig. 6f, prebleach, Supplementary Fig. 10). Through photobleaching of single Z-discs, we established recovery profiles of the different FLNc variants (Fig. 6f, bleach and subsequent images, Supplementary Fig. 10). Mean halftimes were significantly increased for the AA mutant (82 s) in comparison to the DD mutant (27 s) and wild-type FLNc (33 s) (Fig. 6g). Accordingly, the percentage of FLNc in the mobile fraction was reduced for the AA mutant (65%) and increased for the DD mutant (91%), when compared to wild-type FLNc (86%) (Fig. 6h). The small but significant difference in the mobile fraction between DD and wild-type FLNc indicates that most, but not all of the expressed FLNc is phosphorylated in muscle cells.

In conclusion, FILIP1 directly binds to FLNc and this binding is negatively regulated by phosphorylation of S2233 and S2236 in the conserved FLNc d20-specific insert. Dual-site phosphorylation controls FLNc dynamics and mobility in skeletal myotubes. Here, constitutive phosphorylation strongly reduced halftime and increased mobility of FLNc, which closely resembled the behavior of wild-type FLNc in differentiated myocytes.

### Dual-site phosphorylation protects FLNc from degradation

**Fig. 7:**
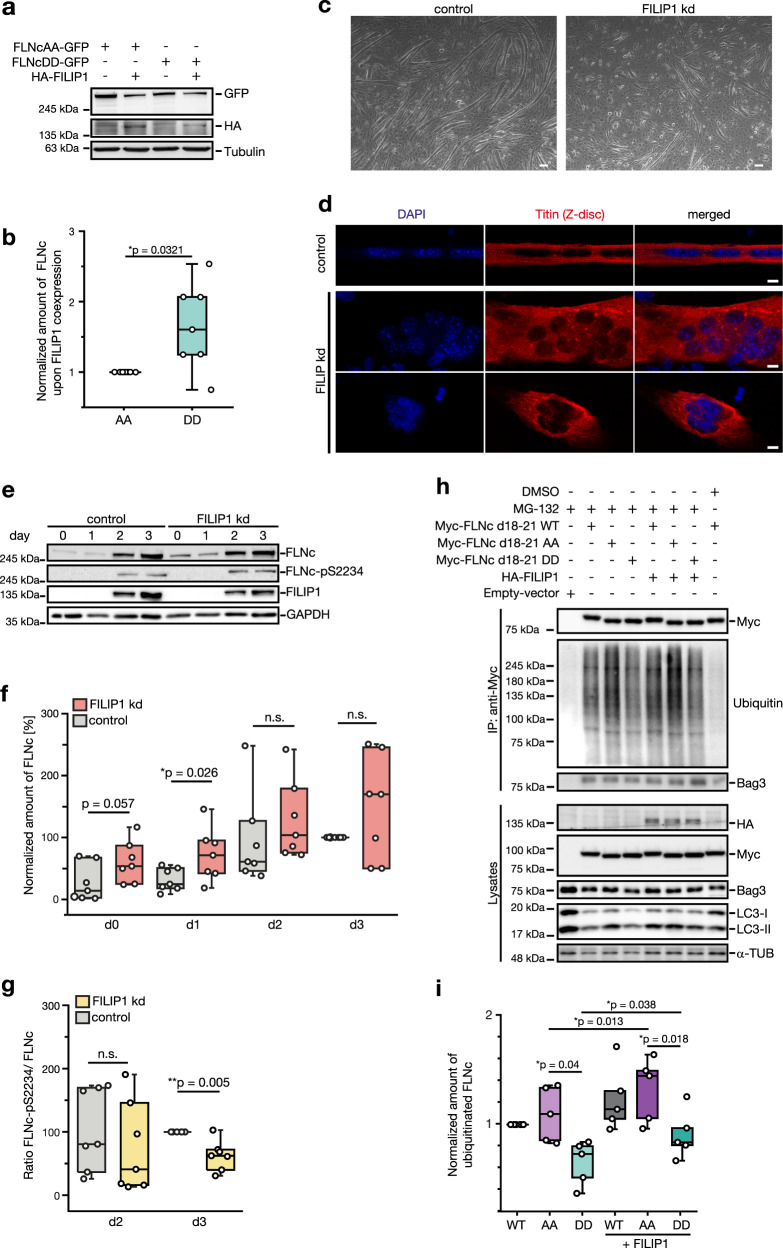
Dual-site phosphorylated FLNc is protected from FILIP1-mediated degradation during myocyte differentiation. **a** Immunoblot analysis of co-expression experiments in C2 skeletal muscle cells transiently expressing GFP fusion proteins of full-length FLNc AA and DD mutants in the presence or absence of HA-tagged FILIP1. **b** Quantification of immunoblot data shown in (**a**). GFP signal intensities were normalized to tubulin signals. Ratios of HA-FILIP1 co-expression to control were normalized to FLNc AA mutant. Data are presented as box and whisker plots; *n* = 7 independent experiments, paired two-tailed student’s *t*-test. **c** Bright-field microscopy images of C2 cells after transfection with FILIP1 siRNA (FILIP1 kd) or scrambled siRNA (control). Images of cells were taken after 4 days of differentiation. Scale bar: 100 µm; kd, knockdown. **d** Fluorescence microscopy images of C2C12 cells after transfection with FILIP1 siRNA (FILIP1 kd) or scrambled siRNA (control). Cells were differentiated for 5 days and fixed cells were stained using antibody against the Z-disc associated part of titin. DAPI was used for nuclei staining. Scale bar: 10 µm. **e**. Immunoblot analysis of FILIP1 kd and control cells. Cells were differentiated 24 h after siRNA transfection and lysed at different time points during differentiation (d0-d3). d, day. **f**, **g** Quantification of immunoblot data shown in (**e**), shown as box and whisker plots; *n* = 7 independent experiments, paired two-tailed student’s *t*-test. **f** FLNc signals in control and FILIP1 kd cells at d0-d3 normalized to the control at d3. **g** Intensity ratios FLNc-pS2233/FLNc at d2 and d3 normalized to the control at d3. **h** Immunoblot analysis of C2 cells transfected with Myc-tagged FLNc d18–21 WT or AA, DD mutants with or without co-expression of HA-FILIP1. Cells were treated 6 hours with 10 µM MG-132 inhibitor or DMSO before lysis. FLNc WT and phosphosite mutants were immunoprecipitated using anti-Myc beads and ubiquitinated species were detected using anti-ubiquitin antibody. **i** Quantification of immunoblot data shown in (**h**). Ubiquitin signal intensities of immunoprecipitated FLNc d18–21 mutants were normalized to Myc signal intensities after immunoprepcipitation. Data normalized to WT are presented as box and whisker plots; *n* = 5 independent experiments, paired two-tailed student’s *t*-test.

Since FILIP1 induced FLNa degradation in neural cells^38^, we asked whether FILIP1 promotes FLNc degradation in skeletal myocytes and whether this process is affected by phosphorylation. We co-expressed full-length FLNc phosphosite variants (AA, DD) with full-length FILIP1 in C2 cells (Supplementary Fig. 7g), and found that the DD mutant was more stable than the AA mutant (Fig. 7a, b and Supplementary Fig. 2). We reasoned that FLNc levels should be increased upon depletion of FILIP1. As previously observed^39^, knockdown of FILIP1 strongly impaired differentiation of myoblasts to myotubes (Fig. 7c and Supplementary Fig. 11). Fluorescence microscopy analysis revealed an almost complete absence of Z-discs and thus functional myofibrils in FILIP1 knockdown cells (Fig. 7d and Supplementary Fig. 12). FILIP1 depletion resulted in increased FLNc levels during myocyte differentiation with a considerably lower ratio of phosphorylated FLNc (pS2234) to total FLNc (Fig. 7e–g). Thus, our data reveal a reduced stability for endogenous FLNc in the presence of FILIP1. Phosphorylated FLNc (pS2234) remained unaltered, confirming that phosphorylation in the extended motif is important for FLNc stability.

To further study FILIP1-mediated degradation of FLNc, we analyzed ubiquitination levels of FLNc d18–21 variants upon proteasome inhibition in the presence or absence of FILIP1 (Supplementary Fig. 7b, g), revealing ubiquitinated FLNc upon proteasome inhibition (Fig. 7h and Supplementary Figs. 13 and 2). Compared to the DD mutant, levels of ubiquitinated FLNc AA were increased and this difference was even more pronounced when FILIP1 was co-expressed (Fig. 7i). MG-132 treatment resulted in increased Bag3 and LC3 levels in C2 cells (Fig. 7h, lower part), indicative of an active Bag3 machinery and chaperone-assisted selective autophagy (CASA), which removes damaged FLNc^40,41^. A small fraction of cellular Bag3 was co-purified with ubiquitinated FLNc (Fig. 7h, upper part), pointing to the degradation of FLNc via CASA.

## Discussion

Even though the PI3K/Akt pathway plays a central role in muscle cells, knowledge about the associated substrate network in skeletal myotubes is still incomplete. To address this gap in knowledge, we quantified relative changes in the phosphoproteome following modulation of PI3K/Akt/mTOR signaling in differentiated skeletal myotubes, which were subjected to mild EPS to ensure the formation of mature Z-discs and contractility^9^. We confirmed pathway activation (IGF-1) and inhibition of PI3K/mTOR (LY294002) and Akt (MK-2206) in starved myotubes under EPS condition (Figs. 1 and 3 and Supplementary Figs. 1 and 4). In this setting, PI3K/Akt/mTOR pathway activity was markedly induced by IGF-1. In contrast, non-exercised human muscle only shows low level Akt activity^30^, and Akt is inactive in both myoblasts^24^ and non-EPS-stimulated differentiated myotubes^3,42^. Our data show that contraction induced in vitro by mild EPS is sufficient to elicit low level PI3K/Akt/mTOR pathway activation in differentiated skeletal myotubes, which is supported by previous work demonstrating that Akt activity rapidly increases in skeletal muscle upon contraction^43^.

We compiled a vast phosphoproteome of contracting skeletal myotubes. This analysis revealed the basophilic motif RxRxxp[S/T]xxpS in phosphopeptides responsive to PI3K/Akt/mTOR pathway inhibition (Figs. 2 and 3 and Supplementary Figs. 1, 3 and 4). A higher preference of Akt towards substrate sites with a serine in +1 and +2 position has been described^44^, which is in accordance with our data showing a preference for serine in +2 position. Furthermore, in the extended motif, glycine and phosphoserine were preferred in +1 and +3 position, respectively (Fig. 2). For most PI3K/Akt/mTOR network components comprising the extended motif, our data reveal phosphorylation of serine/threonine in the central and +3 position (Fig. 3). Several proteins (Ahctf1, eIF4B, ErbB3, FLNc, Mast2, Ndrg1, Ndrg3, Plec, Pom121, Rictor, Sh3bp5, Srpk2) were regulated at both phosphosites in this motif. However, the responsible kinases and functional implications are largely unknown.

To explore the role of dual-site phosphorylation for fine-tuning interactions and function in an exemplary fashion, we investigated the effects of phosphorylations in the extended motif in d20 of FLNc, a signaling adaptor and actin-crosslinking protein predominantly expressed in skeletal and cardiac muscle^15,21^. Loss of FLNc is embryonic lethal in mice^20^ and mutations in FLNc lead to severe skeletal and/or cardiac muscle phenotypes in patients^21^. This strongly indicates that precise control of FLNc homeostasis, dynamics and functions is pivotal for proper muscle cell development, maintenance, and contractility. Our data confirm hFLNc as substrate of Akt at S2233 (mFLNC-S2334^24^) and PKCα at S2236 (+3 position; mFLNc-S2337) (Fig. 4 and Supplementary Fig. 6). Thus, Akt phosphorylates in concert with PKCα FLNc in its unique insert in d20, which is sufficient for Z-disc targeting^26^ and facilitates the interaction with numerous proteins including Xin/XIRP1^32^, myopodin/SYNPO2^33^, myotilin^16^, NRAP^37^ and Bag3^35^. In this work, FILIP1 was identified as an additional direct interaction partner of FLNc in skeletal muscle cells (Figs. 5 and 6, and Supplementary Figs. 8 and 9).

**Fig. 8:**
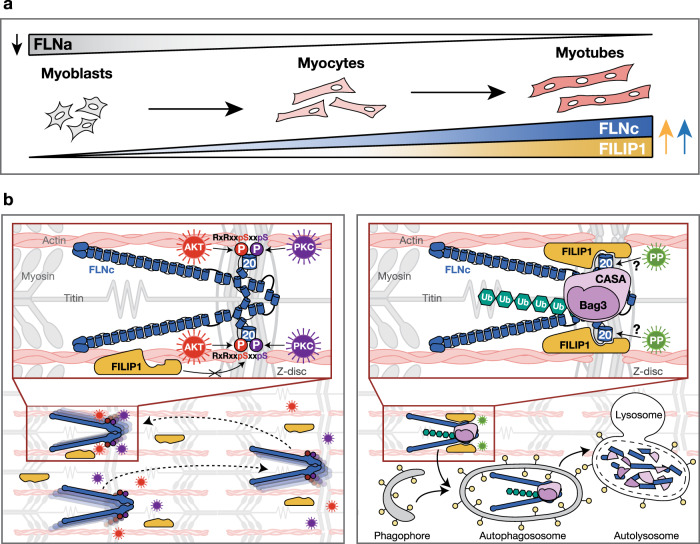
Model of the regulation of FILIP1-mediated FLNc degradation. **a** Differentiation-dependent expression of FILIP1, FLNc and FLNa in skeletal muscle cells. **b** Akt- and PKCα-dependent dual-site phosphorylation occurs in the extended basophilic motif located in the unique insert in domain 20 of FLNc. Dual-site phosphorylation reduces binding to FILIP1 and ensures stability and high dynamics of FLNc in muscle cells. Activation of protein phosphatases (PP) may result in the dephosphorylation of FLNc and thus, FILIP binding promoting the removal of nonphosphorylated FLNc of low mobility and dynamics by the Bag3-machinery and chaperone-assisted selective autophagy (CASA). Ub, ubiquitin.

In muscle cells, expression of *FILIP1* mRNA is regulated by the long non-coding RNA Myolinc and *FILIP1* knockdown inhibits the differentiation of myoblasts into myotubes^39^. While *FILIP1* mRNA is not expressed at detectable levels in myoblasts, its expression begins at day 2 in differentiating cultured C2C12 cells and steadily increases until cells are fully differentiated^39^. Thus, the observed differentiation-dependent *FILIP1* mRNA expression pattern correlates well with FILIP1 protein levels. FLNa levels, however, are highest in myoblasts when FILIP1 is not expressed and strongly decrease with FILIP1 expression (Fig. 5c, summarized in Fig. 8a). Concomitantly, the timely controlled degradation of FLNa was shown to be essential for differentiation and sarcomere formation in cardiac myocytes^45^. We also show that distinct from FLNa, the expression of FLNc strongly increases during differentiation of C2C12 cells (Fig. 5c; see also ref. ^26^ and Fig. 8a). In line with this, FLNc was found to be essential for proper myogenesis and the maintenance of structural integrity both in vitro and in vivo^20,46^. The fundamental importance of FLNa homeostasis for cell differentiation was demonstrated in neuronal cells, where FILIP1-mediated FLNa degradation controls neocortical neuronal cell migration from the ventricular zone^38^. Our data therefore suggest a mechanism for FILIP1-dependent clearance of FLNa in differentiating muscle cells, while simultaneously FLNc is protected by Akt/PKCα-mediated phosphorylation. Several lines of evidence support this hypothesis: (i) In contracting skeletal muscle cells, mFLNc is phosphorylated at S2234 to a high extent even at low Akt activity (Fig. 1b, c. Supplementary Fig. 4h and Supplementary Data 1) which qualifies it as a high-quality substrate of Akt^47^. (ii) Concurrent Akt- and PKCα-dependent dual-site phosphorylation of FLNc within the extended motif, located in a FLNc-specific insert in d20^48^, prevails in cells (Figs. 3 and 4, Supplementary Figs. 4f–i and 9c) (iii). Our interaction data consistently show that dual-site phosphorylated FLNc binds FILIP1 significantly weaker than non-phosphorylated FLNc (Fig. 6a–e and Supplementary Fig. 9). (iv) Our microscopical analyses in living skeletal muscle cells demonstrate that wild-type, as well as AA and DD FLNc phosphosite mutants localize to myofibrillar Z-discs, indicating that Z-disc targeting per se is not regulated by phosphorylation. However, our FRAP data implicate that dual-site phosphorylation of d20 is mandatory to ensure the extremely high dynamics and mobility of FLNc (Fig. 6f–h). This finding is in accordance with earlier work showing that phosphorylated FLNc has a lower apparent binding affinity to isolated myofibrils^14^ and supports our previous observation that PKCα controls FLNc dynamics^9^, a property that is essential for the role of FLNc as signaling adaptor protein in myocytes. (v) We show that the phosphomimicking DD mutant of FLNc is significantly more stable in the presence of FILIP1 compared to the AA mutant that binds much stronger to FILIP1 (Fig. 7a, b). (vi) Total levels of endogenous FLNc (i.e., corresponding to both the phosphorylated and non-phosphorylated form) were increased in FILIP1-depleted differentiating myocytes (Fig. 7c–f). (vii) FLNc phosphorylation stabilized the protein and protected it against FILIP1-associated degradation (Fig. 7g). (viii) Inhibition of the proteasome resulted in increased ubiquitination of non-phosphorylated FLNc, especially upon co-expression of FILIP1 (Fig. 7h, i). The associated increased LC3-II levels that we detected in myocytes treated with MG-132 confirms the observation that inhibition of the proteasome induces the Bag3-machinery and CASA^49^. Indeed, the co-chaperone Bag3 bound ubiquitinated FLNc, which points to degradation via this machinery of which FLNc is a known client^40,41^. Thus, our data indicate that FILIP1 may help to recruit the CASA machinery to degrade non-phosphorylated FLNc. The activation of protein phosphatases under stress conditions, and the concurrent dephosphorylation of FLNc may thus be a mechanism for selective autophagic removal of less- or non-functional FLNc mediated by FILIP1.

In sum, Akt- and PKCα-mediated dual-site phosphorylation of FLNc in its unique insert in d20 provides a mechanism that selectively protects phosphorylated FLNc, but not non-phosphorylated FLNc or other filamins, from FILIP1-mediated degradation. This dual mechanism negatively modulates binding to FILIP1 and ensures both stability and high dynamics of FLNc in contracting cross-striated muscle cells (Fig. 8b). Further work is needed to evaluate the role of the described dual-site phosphorylation of FLNc for precise regulation of its function as actin cross-linker and signaling adaptor in cross-striated muscle. We further propose that FILIP1-mediated homeostasis of filamins is required for the proper development of skeletal and cardiac muscle cells and likely also their maintenance under conditions of sustained mechanical stress.

## Methods

### Cell culture

For stable isotope labeling by amino acids in cell culture (SILAC), C2 myoblasts were cultured in high glucose SILAC-DMEM medium (PAA, GE Healthcare Life Sciences, Freiburg, Germany) supplemented with 15% dialyzed fetal bovine serum (FBS, PAA), 1% non-essential amino acids, 1% penicillin/streptomycin, 1% sodium pyruvate, 1% proline (all Life Technologies) as well as 84 mg/l arginine and 146 mg/l lysine (Cambridge Isotope Laboratories Inc., Tewksbury, USA) for at least nine cell doublings. Light, medium heavy and heavy stable isotope labeling of cells was performed with ^13^C_6_-l-arginine/^12^C_6_-l-lysine, ^12^C_6_-l-arginine/D_4_-l-lysine and ^13^C_6_^15^N_4_-l-arginine/^13^C_6_^15^N_2_-l-lysine, respectively. Stable isotope-labeled myoblasts were seeded into 6-well plates (Corning Incorporated, New York, USA) and grown to 90% confluency. Differentiation of cells was induced by reduction of the dialyzed FBS or in-house dialyzed horse serum content to 2% in the absence of sodium pyruvate. Differentiation medium was changed every 48 h until day 4. Subsequently, cells were serum-starved for 16 h and sarcomere formation was improved by EPS (0.05 Hz, 4 ms, 10 V) for 4 h using a C-Pace EP Culture Pacer (IonOptix, Milton, USA). One hour prior to cell lysis, cells were either treated with 10 ng/ml IGF-1, 10 μM LY294002 or DMSO for the IGF1/LY study. Experiments were performed in three independent biological replicates with a label switch.

For the SILAC-based LY/MK study, contracting myotubes were either treated with 10 ng/ml IGF-1, 10 ng/ml IGF-1 + 10 μM LY294002 or 10 ng/ml IGF-1 + 10 μM MK-2206 30 min prior to cell lysis. Experiments were performed in six independent biological replicates and two technical replicates with label switches.

Human embryonic kidney 293 T (HEK293T) cells were cultured in DMEM supplemented with 10% FBS and 1% sodium pyruvate. HEK293 cells were transfected using polyethylenimine (PEI, 1 µg/µl, Polysciences Europe GmbH), which was mixed in a 3:1 ratio with DNA, diluted with a 6-fold volume of Opti-MEM (Life Technologies) and incubated for 15 min prior transfection. C2 cells were transfected using Lipofecamine2000 (Invitrogen, Darmstadt, Germany) or jetPRIME (VWR, Darmstadt, Germany). Reagents and DNA were mixed according to the manufacturer´s protocol and myoblast cells were seeded in Opti-MEM (for Lipofecamine2000 transfection) or culture medium (for jetPRIME transfection) for in-solution transfection. Medium was changed to culture medium after 6 h and cells were lysed 24–48 h after transfection. C2C12 cells were grown in high-glucose DMEM supplemented with 1% non-essential amino acids, 1% penicillin/streptomycin, 1% sodium pyruvate (all LifeTechnologies) and 15% FBS (Sigma). Differentiation was initiated by exchanging the FBS in the culture medium by 2% horse serum (LifeTechnologies). Cells were regularly tested for mycoplasma contamination and found to be mycoplasma-negative.

### Sample processing

Cell lysis of C2 myotubes was performed using urea lysis buffer (7 M urea, 2 M thiourea, 1 mM sodium orthovanadate, 10 mM β-glycerophosphate, 9.5 mM sodium fluoride and 10 mM sodium pyrophosphate). In brief, cells were placed on ice and washed twice with ice-cold phosphate-buffered saline (PBS), supplemented with 0.75 mM CaCl_2_ and 0.75 mM MgCl_2_. 150 µl urea lysis buffer were used for each well of a 6-well plate. Cells were scraped from the dish, sonicated two times for 10 s on ice and centrifuged at 21,000 *g*. Protein concentrations of supernatants were equalized using the Bradford assay (BioRad, Munich, Germany) and control, IGF-1- and LY294002-treated samples were mixed in a 1:1:1 ratio equal to a total protein amount of 9 mg. Reduction and alkylation of proteins was performed as described^9^ with slight modifications. Each reaction was carried out for 20 min using a final concentration of 5 mM tris(2-carboxyethyl)phosphine (TCEP) and 50 mM 2-chloroacetamide before alkylation was quenched with 5 mM DTT. For tryptic digestion, protein samples were diluted 1:4 (v/v) with 50 mM ammonium bicarbonate solution and incubated with sequencing grade trypsin (Promega) in a 1:50 (w/w) ratio for 3.5 h at 42 °C and 200 rpm. Peptides were desalted using an Oasis HLB cartridge (Waters Corporation, Milford, USA) according to the manufacturer’s protocol. Eluates were aliquoted, lyophilized and stored at −80 °C. In addition, cells in one well of a 6-well plate were lysed with 150 μl modified RIPA buffer [50 mM Tris, 150 mM NaCl, 1% NP-40, 0.25% sodium deoxycholate, pH 7.6] supplemented with phosphatase and protease inhibitors [1 mM sodium orthovanadate, 10 mM β-glycerophosphate, 9.5 mM sodium fluoride, 10 mM sodium pyrophosphate, protease inhibitor (Roche)] for Western blot analyses.

### Peptide fractionation

For peptide fractionation, a Dionex Ultimate 3000 UHPLC system equipped with a Polysulfoethyl-A column (Ø: 4.6 mm, 20 cm, 5 μm, 200 Å, PolyLC, Columbia, USA) was used with a binary solvent system consisting of strong cation exchange (SCX) buffer A [5 mM potassium dihydrogen phosphate, 20% ACN (v/v), pH 2.8] and buffer B [5 mM potassium dihydrogen phosphate, 20% ACN (v/v), 500 mM KCl, pH 2.8]. Tryptic digests were dissolved in 400 μl buffer A, centrifuged and the supernatant was loaded onto the SCX column. Peptides separation was performed at a flow rate of 700 μl/min using a linear gradient of 0–30% SCX buffer B in 50 min starting 10 min after sample injection. Subsequently, the gradient was ramped from 30 to 50% and 50 to 100% solvent B in 10 min each, held at 100% B for 5 min and re-equilibrated for 20 min with 100% buffer A. Three-minutes fractions (30 fractions per run) were collected and each fraction was used for titanium dioxide (TiO_2_)-based enrichment of phosphopeptides. Samples from three independent biological experiments were further processed for phosphopeptide enrichment in a randomized procedure. Before enrichment, an aliquot of each SCX fraction was taken, dried in vacuo and stored at −80 °C for LC-MS.

### Phosphopeptide enrichment

In the IGF-1/LY study, phosphopeptides were enriched using TiO_2_ spherical beads as described before^9^ with slight modifications. Briefly, 30 μl of TiO_2_ material (TiO_2_, 5 μm, GL Science Inc.) resuspended in ACN 1:1 (v/v) were used for SCX fractions and 7.5 μL for Myc-tag enriched FLNc peptides. TiO_2_ beads were washed twice (80% ACN, 0.1% TFA) and pre-equilibrated with 50 μl loading buffer [20% (v/v) acetic acid, 20 mg/ml 2,3-dihydroxybenzoic acid, 420 mM octasulfonic acid, 0.1% (v/v) heptafluorobutyric acid]. Peptide samples were mixed 1:1 (v/v) with 2x loading buffer, added to pretreated TiO_2_ beads, vortexed and incubated for 20 min at 4 °C with slight agitation. After incubation, the material was washed twice as before and incubated with 50 μl elution buffer (50 mM ammonium dihydrogen phosphate, pH 10.5) for 15 min. Subsequently, 50 μl ACN were added and supernatants were acidified with 8 μl TFA on ice, split in two equal parts, and dried in vacuo.

In the LY/MK study, phosphopeptides were first enriched using TiO_2_ spherical beads using the EasyPhos method^50^ in two technical replicates. Phosphopeptides were also enriched from each of the supernatants using sequential metal oxide affinity chromatography (SMOAC)^51^. In brief, the supernatant and the first washing step from the TiO_2_ enrichment procedure were combined and lyophilized overnight. Dried peptides were resolved in 0.1% TFA and desalted using C18 solid phase extraction cartridges (3 M Empore) according to manufacturer’s protocol. Eluted peptides in 80% ACN were loaded onto 100 μl self-made Fe(III)-IMAC resin. To this end, Ni^2+^-NTA beads (Quiagen) were washed three times with pure water, incubated for 30 min with 100 mM EDTA on a rotation wheel and washed further three times. Subsequently, beads were incubated with 10 mM FeCl_3_ for 30 min on a rotation wheel to obtain Fe(III)-IMAC beads. Washed beads were loaded with desalted samples and rotated for 30 min. Following three washing steps with 80% ACN/0.1% TFA, samples were eluted from the beads using 2 × 30 µl elution buffer (50% ACN, 5% ammonia water) and a magnetic rack. Phosphopeptides were dried in vacuo and stored at −80 °C before LC-MS analysis.

### LC-MS analysis

Reversed-phase liquid chromatography-mass spectrometry was performed using the UltiMateTM 3000 RSLCnano system (Dionex LC Packings/Thermo Fisher Scientific, Dreieich, Germany) coupled online to a Velos Orbitrap Elite or a Q Exactive Plus (both Thermo Fisher Scientific, Bremen, Germany) instrument. The UHPLC system was equipped with two C18 μ-precolumns (Ø 0.3 mm × 5 mm; PepMap™, Thermo Fisher Scientific) and an Acclaim® PepMap™ analytical column (ID: 75 μm x 500 mm, 2 μm, 100 Å, Dionex LC Packings/Thermo Fisher Scientific). This LC setup was used for the IGF-1/LY294002 phosphoproteome, PRM analyses, BioID experiments and FILIP1 pull-down analysis. For the LY294002/MK-2206 phosphoproteome and FLNc pull-down analyses the UHPLC system was equipped with two C18 pre-columns (μPAC™ trapping column, PharmaFluidics) and a C18 endcapped analytical column (50 cm μPAC™ column, PharmaFluidics). The MS instruments were externally calibrated using standard compounds and equipped with a nanoelectrospray ion source and a stainless steel emitter (Thermo Fischer Scientific). MS/MS analyses were generally performed on multiply charged peptide ions applying a normalized collision energy (NCE) of 35% with an activation q of 0.25 and an activation time of 30 ms unless otherwise stated. Peptide mixtures from SCX fractions were analyzed by LC-MS using a 2 h LC gradient and CID for peptide fragmentation. Phosphopeptide-enriched mixtures from SCX fractions were each analyzed in two technical replicates applying a 2 h LC gradient and multistage activation (MSA) with neutral loss (NL) masses of 32.7, 49 and 98 Da for technical replicate 1 and higher-energy collisional dissociation (HCD) with a NCE of 28% for technical replicate 2. Phosphopeptide samples from EasyPhos and SMOAC experiments were each analyzed in two technical replicates applying a 4 h LC gradient and HCD for peptide fragmentation with a NCE of 28% on a QExactive Plus instrument.

### Parallel reaction monitoring assay

Mouse C2 cells transiently expressing BirA* FLNc d18–21 were treated with 10 μM MK-2206 (Selleck) or 10 μM Gö6976 (Merck) for 1 h or with 300 nM PMA (Sigma Aldrich) or IGF-1 (Sigma Aldrich) for 15 min. In experiments using both, inhibitor and activator, cells were first treated with the respective inhibitor (MK-2206, Gö6976) for 1 h and PMA or IGF-1 were added for the last 15 min of the treatment. Cells, seeded on 6 cm dishes, were lysed on ice with 500 µl modified RIPA buffer. Enrichment of the Myc-tagged FLNc fragment was performed with 10 μl Myc-Dynabeads (Invitrogen) according to the manufacturer’s protocol. On-bead tryptic digestion was performed in 200 µl ammonium bicarbonate solution (50 mM) for 3.5 h at 42 °C and 800 rpm on a thermoshaker using sequencing grade trypsin (Promega) in a 1:50 (w/w) ratio. Phosphopeptides were enriched as described above and mixed with 47.5-95 fmol phosphopeptides of a phosphopeptide standard comprising 181 phosphopeptides (Intavis). Samples from 4 biological replicates were analyzed in randomized order by LC-MS using an 1 h LC gradient and HCD (NCE of 28) for peptide fragmentation on a Q Exactive Plus instrument. For parallel reaction monitoring (PRM), an inclusion list comprising 16 precursors with 96 transitions was generated with Skyline 2.6.0.6709 and is available in the PanoramaWeb interface. One scan cycle consisted of a full MS1 scan at a resolution of 70,000, an automatic gain control (AGC) target of 3e6 ions, a max. ion time of 20 ms and a scan range from *m*/*z* 400 to 1600, followed by 10 PRM scans. Each PRM scan targeted one precursor of the inclusion list at a resolving power of 17,500, an AGC target of 2e5, a max. ion time of 49 ms and an isolation window of 2 *m*/*z*. Raw files were analyzed with Skyline and intensities of hFLNc phosphopeptides were normalized to the summed intensities of eight phosphopeptides (SEGpSPVLPHEPAK, TGMGSGpSAGKEGGPFK, pSTVASMMHR, VIEDNEpYTAR, LIEDNEpYTAR, pSFNGSLKNVAVDELSR, pSGGQRHSPLSQR, AYpTHQVVTR) from the phosphopeptide standard.

### Proximity-dependent biotin identification

SILAC-encoded C2 cells transiently expressing BirA* or BirA* FLNc d18–21 were differentiated for 4 days and subjected to EPS (4 ms, 10 V, 0.05 Hz) for 4 h prior to cell lysis. To enhance biotinylation, 50 μM biotin (Amresco, Solon, USA) were added to the culture medium 24 h prior to cell lysis. One set of cells expressing BirA* FLNc d18–21 was cultured without adding biotin and served as an additional control. Cell lysis was performed as described^34^. In brief, cells were washed three times with PBS, lysed in lysis buffer [50 mM Tris, pH 7.4, 500 mM NaCl, 0.4% SDS, 5 mM EDTA, 1 mM DTT, and 1x Complete protease inhibitor (Roche)] and sonicated. Triton X-100 was added to 2% final concentration and lysates were sonicated. An equal volume of 50 mM Tris (pH 7.4) was added before additional sonication and lysates were centrifuged at 16,000 *g*. Protein concentrations of supernatants were equalized using the Bradford assay followed by mixing of samples in a 1:1:1 ratio to a final protein amount of 900 μg per replicate. Coupling of biotinylated proteins to streptavidin Dynabeads (Invitrogen, Karlsruhe, Germany) and subsequent washing steps were performed as described^34^. In brief, samples were incubated with 600 µl Dynabeads overnight. Beads were washed twice with 1 ml wash buffer 1 (2% SDS in dH2O) at RT, once with wash buffer 2 (0.1% deoxycholate, 1% Triton X-100, 500 mM NaCl, 1 mM EDTA, and 50 mM Hepes, pH 7.5) and wash buffer 3 (250 mM LiCl, 0.5% NP-40, 0.5% deoxycholate, 1 mM EDTA, and 10 mM Tris, pH 8.1), and twice with wash buffer 4 (50 mM Tris, pH 7.4, and 50 mM NaCl). For tryptic on-bead digestion, protein samples were washed twice with 50 mM ammonium bicarbonate solution and incubated with sequencing grade trypsin (Promega) in a 1:50 (w/w) ratio for 3.5 h at 42 °C and 200 rpm on a thermoshaker. Supernatants were acidified to a final concentration of 1% TFA on ice, split in four equal parts and dried in vacuo. Tryptic peptide mixtures from three biological replicates were analyzed each in two technical replicates by LC-MS using a 3 h LC gradient and CID for peptide fragmentation on a Velos Orbitrap Elite instrument.

### In vitro kinase assay

Recombinantly expressed and purified human FLNc d18–21 was dialyzed overnight at 4 °C in dialysis buffer (1 mM DTT, 100 mM KCl, 20 mM HEPES pH 7.4, 10 mM MgCl_2_). For MS-coupled kinase assays, H_2_O was added to 100 μg protein to a total volume of 200 μl and mixed with 10× kinase buffer (NEB, Frankfurt, Germany). The assay was started by adding 200 ng Akt (Proteinkinase, Kassel, Germany) and/or 200 ng PKCα (Sigma-Aldrich) in the presence of 1× PKC lipid activator (Merck Millipore, Darmstadt, Germany). The reaction was carried out for 20 min at 30 °C and 200 rpm. One tenth of each of three independent replicates was used for analysis by SDS-PAGE and immunoblotting using an antibody directed against the EEF-tag fused carboxy-terminally to FLNc d18–21. The remaining sample was diluted 1:4 (v/v) with 50 mM ammonium bicarbonate and subjected to in-solution digestion using sequencing grade trypsin (1:50) (Promega) for 3.5 h at 42 °C and 200 rpm on a thermoshaker. Single protein digests were acidified with TFA [final concentration 1% (v/v)]. Phosphopeptides were enriched using TiO_2_ beads and analyzed by LC-MS applying a 1 h LC gradient and MSA, HCD or electron transfer dissociation (ETD) for peptide fragmentation. The activation time for ETD fragmentation was set to 100 ms. using MSA, HCD and ETD fragmentation.

### Bioinformatics

For quantitative phosphoproteomic data of the IGF-1/LY study, Andromeda integrated in MaxQuant 1.5.1.0^52^ was used to search peak lists against the UniProt ProteomeSet mouse database (release 12.2016, 58,430 protein entries). For the LY/MK study, MaxQuant 1.6.10.43 was used with the UniProt ProteomeSet mouse database (release 11.2019, 63,405 protein entries). The precursor mass tolerance was set to 20 ppm for the first search and to 4.5 ppm for the main search. For MSA/CID data, the fragment mass tolerance was set to 0.5 Da. Trypsin was set as proteolytic enzyme (≤2 missed cleavages). Oxidation of methionine and phosphorylation of serine (S), threonine (T) and tyrosine (Y) were set as variable modifications and cysteine carbamidomethylation as fixed modification. A false discovery rate (FDR) of 1% was applied on both peptide (on modified peptides separately) and protein lists. Numbers of unique phosphopeptides were counted based on the MaxQuant peptide ID in the Phospho(STY) sites table. Phosphosites with a MaxQuant localization probability of ≥0.75 were deemed “localized”, while sites with a localization probability of <0.75 were counted as putative sites given that the amino acid (aa) sequence in combination with the number of phosphate groups was not identified with localized sites elsewhere in the dataset. Subsequently, the intensity ratios of all localized sites were analyzed statistically at the log_2_-scale. A linear model was applied to jointly estimate IGF-1 and LY treatment effects and for assessing their significance. In the EasyPhos-SMOAC analysis, the effects of the LY and MK inhibitors with simultaneous IGF-1 treatment were analyzed. In this analysis, two labeling effects were included into the linear model to adjust for a labeling bias. The estimated residual variances of the individual proteins were regularized by averaging with the median variance of all proteins. False-discovery rates were calculated by the linear step-up procedure introduced by Benjamini and Hochberg. In the IGF-1/LY study, phosphopeptides with a fold-change > 1.5 and a *p* value < 0.05 were considered as regulated. These peptides were further analyzed with the R motif-X package^53^ to identify enriched motifs. The +/− 7 residues sequence windows of phosphorylated residues within the regulated peptides were used for the analysis. The default motif-X parameter, minimum motif occurrence of 10 and a *p* value cutoff of 1e-6 were used. For the visualization of the sequence logos, the Python package Logomaker^54^ was used. In the LY/MK study, phosphopeptides with a fold-change > 1.5 and a moderated adjusted *p* value < 0.01 were deemed significantly regulated.

For the kinase-substrate enrichment analysis (KSEA)^27^ quantified phosphopeptides were annotated with their respective kinases according to the PhosphositePlus^55^ kinase-substrate dataset (downloaded July 2019), filtered for kinase-substrate interactions reported in vivo for mice. Kinase isoforms were grouped prior to calculating the kinase score and only kinases with at least 5 mapped substrates were regarded in the further analysis. The normalized kinase score was calculated with the following equation based on a z-score transformation^27^: ${\textstyle{{\left( {\bar s - \bar p} \right)\surd m} \over \sigma }}$. Where, $\bar s$ denotes the mean log_2_ fold-change of a kinase’s substrates, $\bar p$ the log_2_ fold-change of all quantified phosphopeptides, m denotes the total number of quantified phosphopeptides attributed to the respective kinase and σ is the standard deviation of the log_2_ fold-change of all phosphopeptides.

For the hierarchical cluster analysis, the IGF-1/LY and LY/MK datasets were combined and the phosphopeptides were filtered for at least one log_2_ fold-change above 0.58 and an adjusted *p* value ˂ 0.05 and ˂ 0.01 for IGF-1/LY and LY/MK data, respectively. Missing values were imputed on the basis of a normal distribution (downshift: 2σ; width: 0.3σ). A Pearson’s correlation-based distance matrix was used for the hierarchical clustering with Ward’s method. For heatmap visualization row-wise z-score transformation was performed. Functional enrichment analysis of Reactome^29^ terms associated with the obtained clusters was conducted as multi-query in the online tool g:profiler^56^ (version: e98_eg45_p14_ce5b097, database update: 02 Oct 2019) using default parameters.

For analysis of MS data from in vitro kinase assays, raw files were processed using Andromeda embedded in MaxQuant 1.5.5.1 and searched against the sequences of human FLNc d18–21 using the UniProt ProteomeSet *Escherichia coli* database (release 01.2018, 4,326 protein entries) as background for FDR calculation. Precursor and fragment mass tolerances were set to 10 ppm and 0.5 Da, respectively. Search parameters were as follows: proteolytic enzyme: trypsin (≤2 missed cleavages), variable modifications: methionine oxidation and S/T/Y phosphorylation. MaxQuant msms.txt files, all raw files and the FLNc d18–21 sequence were imported into Skyline 4.1.0^57^. MS1 intensities were calculated using the MS1 filtering tutorial provided by the software developers. Skyline peptide settings were as follows: tryptic peptides (≤1 missed cleavage); time window, 3 min; min. and max. peptide length 8 and 30 aa, respectively; exclusion of cysteine peptide; variable modifications (max. number 3), S/T/Y phosphorylation and methionine oxidation; neutral losses, max. number 1. Orbitrap default parameters were used for transition settings. Extracted ion chromatograms of imported peptides were manually inspected for correct peak picking and peak integration was adjusted, if necessary. Total MS1 areas for all peptides with ≥6 MS/MS spectra and a mass error of ≤3 ppm were exported into a pivot table and processed using Excel2010 and Origin 9.1. Mean and standard error of the mean (SEM) were calculated for technical replicates and then for biological replicates. Intensities of all phosphopeptides were summed and normalized by the respective summed intensity.

Raw files from PRM analyses were pre-processed into mascot generic files using ProteomeDiscoverer 1.4 and searched against Uniprot ProteomeSet database (release 11.2016, 92,933 protein entries) including the sequence of FLNc d18–21 and its phosphosite mutants as background for FDR calculation. Precursor and fragment mass tolerances were set to 5 ppm and 0.1 Da, respectively. Search parameters were: proteolytic enzyme, trypsin (≤1 missed cleavages); variable modifications, methionine oxidation and S/T/Y phosphorylation. Mascot.dat files, all raw files and FLNc d18–21 sequences were imported into Skyline 4.1.0, processed as described above and visualized by Instant Clue^58^.

Raw files from BioID experiments were searched with MaxQuant 1.5.5.1 against the Uniprot ProteomeSet mouse database (release Jan 2016, 58,790 protein entries) supplemented with the fusion protein BirA*FLNc d18–21. Trypsin was set as proteolytic enzyme (≤2 missed cleavages), cysteine carbamidomethylation as fixed modification, N-terminal acetylation, methionine oxidation and S/T/Y phosphorylation as variable modifications. The precursor mass tolerance the first search was set to 20 ppm and to 4.5 ppm for main search. The mass tolerance of fragment ions was set to 0.5 Da. A FDR of 1% was applied on both peptide (on modified peptides separately) and protein lists and a minimum of one unique peptide was enabled for protein identification. The protein groups file was processed using Perseus 1.5.2.6 and OriginPro 9.0. Reverse and potential contaminant hits as well as proteins positive for “only identified by site” were removed. Data were filtered for at least three SILAC ratios in each biological replicate and log_10_-transformed mean SILAC ratios were calculated. Mean log_10_ ratios were subjected to hierarchical clustering by Euclidean average *k*-means cluster analysis. Gene Ontology (GO) analysis for cellular component (release 30 Apr 2015) was performed using the Cytoscape 3.3.0 app ClueGO 2.1.7^59^.

Raw files from FILIP1 pull-down analyses were pre-processed into mascot generic files using ProteomeDiscoverer 1.4 and searched against Uniprot ProteomeSet mouse database (release Mar 2016, 58,761 protein entries) with Mascot 2.4.0. Precursor and fragment mass tolerances were set to 5 ppm and 0.1 Da, respectively. Search parameters were: proteolytic enzyme, trypsin (≤3 missed cleavages); variable modifications, methionine oxidation and S/T/Y phosphorylation.

Raw files from FLNc d18–21 and FLNc d1–3 pull-down analyses were searched with MaxQuant 1.6.10.43 using the UniProt knowledge base isoform mouse database (release July 2019, 95,824 protein entries) with additional sequences from hFLNc d18–21 and hFLNc d1–3. Precursor and fragment mass tolerances were set to 4.5 ppm and 0.1 Da, respectively. Search parameters were: proteolytic enzyme, trypsin (≤2 missed cleavages); variable modifications, methionine oxidation and acetylation (Protein N-term); fixed modification, carbamidomethylation.

Text mining for the identification of kinases potentially phosphorylating significantly regulated peptides was performed on publicly available biomedical literature sources, i.e., the entire Medline and the open access subset of PubMed Central databases. As of February 2020, Medline contained over 30 million abstracts and the open access content of PubMed Central comprised over 2.7 million full texts that were eligible for an automated literature scan. Tools from the JCoRe repository were used for linguistic processing, performing tasks such as segmentation of each text into basic linguistic units (sentences and words), acronym recognition (to determine the long forms of abbreviation terms), and grammatical analysis^60^. For subsequent semantic analysis, GeNo tagger was used to identify gene and protein occurrences in text^61^ followed by running the BioSem tool to identify molecular events between previously recognized gene and protein mentions^62^. Event items referencing members of significantly regulated phosphopeptides were extracted and further post-processed to obtain the final result list. To optimize recall of the final result, UniProt Accession IDs were sought for all given gene names using the mapping tool ‘UniProt Mapping Service’ provided at the UniProt website (http://www.uniprot.org/mapping/). These identifiers were ultimately mapped to all associated EntrezGene identifiers resulting in an extended list of Entrez IDs, which, in turn, were used for querying event selection. These identifiers were ultimately mapped to all associated EntrezGene identifiers resulting in an extended list of Entrez IDs which, in turn, were used for event selection.

### Yeast two-hybrid and pull-down assays

Direct yeast two-hybrid experiments were performed by co-transforming L40 yeast cells with different cDNA fragments of human FLNc cloned into a modified pLex vector and a FILIP1 construct encoding its carboxy-terminus in a modified pAct2 vector. An interaction of bait and prey proteins was tested by transformation of constructs into L40 cells, culturing on selective plates and analysis of β-galactosidase activity as described previously^16^. Expression and purification of recombinant His_6_- or GST-fusion proteins were performed according to standard protocols described before with minor modifications^33^. In brief, *E. coli* BL21(DE3) cells were grown in LB medium (1% tryptone, 0.5% yeast extract, 0.5% NaCl, pH 7.3) supplemented with 100 mg/l ampicillin (and, for His_6_-tagged proteins, 34 mg/l chloramphenicol). Protein expression was induced at OD_600_ = 0.6 by addition of 1 mM IPTG. After 4 h at 37 °C, cells were harvested by centrifugation. For His_6_-tagged proteins, cells were resuspended in 4 ml lysis buffer (50 mM NaH_2_PO_4_ pH 8.0, 300 mM NaCl, 10 mM imidazole, 1 mg/ml lysozyme), sonicated and centrifuged (3,800 *g*, 30 min, 4 °C). The supernatant was incubated with Ni^2+^-NTA agarose beads (Qiagen, Hilden, Germany) under constant rotation at 4 °C for 1 h. Beads were transferred to a column and washed five times with 4.5 ml washing buffer (50 mM sodium phosphate, pH 8.0, 300 mM NaCl, 20 mM imidazole). Bound protein was eluted with elution buffer (50 mM sodium phosphate, pH 8.0, 300 mM NaCl, 250 mM imidazole). Purification of GST-tagged proteins was performed similarly using glutathione-agarose beads (Macherey-Nagel, Düren, Germany) and following buffers: lysis buffer (50 mM NaH_2_PO_4_ pH 8.0, 300 mM NaCl), washing buffer (50 mM Tris pH 8.0, 150 mM NaCl). For FILIP1 pull-down experiments, GST-FILIP1 carboxy-terminus (CT) fusion protein immobilized on glutathione-agarose beads was incubated with purified His_6_ and EEF-tagged FLNc fragments (FLNc d1–3, FLNc d18–21 WT, AS, AA, DS, DD) under constant agitation at 4 °C for 1 h. Beads were washed with GST washing buffer and samples were eluted by addition of Laemmli sample buffer at 95 °C. Subsequently, samples were analyzed by SDS-PAGE followed by quantitative Western blot analysis using antibodies directed against the respective tag of the protein. Alternatively, His_6_-tagged FILIP1-2 CT was used to pull-down putative binding partners from extracts prepared from C2C12 cells. Differentiated C2C12 myotubes were scraped in PBS from four 10 cm culture dishes, pelleted and frozen until use. Cell pellets were lysed in 3 ml lysis buffer (50 mM Na_2_HPO_4_, 300 mM NaCl, 20 mM imidazole, 0.5% (w/v) Tween-20, pH 8.0), and extensively sonicated (UP50H, Hielscher, Teltow, Germany). Insoluble material was removed by centrifugation (30 min, 4000 *g*). 1 ml of the cleared lysates was incubated while rotating at 4 °C for 1 h with 150 µl empty Ni^2+^-NTA-agarose beads or beads with bound FILIP1-2 CT (aa 776–1177). As a control, beads with bound FILIP1-2 CT were incubated with lysis buffer only. After extensive washing (50 mM Na_2_HPO_4_, 300 mM NaCl, 40 mM imidazole, pH 8.0), FILIP1-2 CT with associated proteins was eluted from the beads by addition of 120 µl elution buffer (50 mM Na_2_HPO_4_, 300 mM NaCl, 200 mM imidazole, pH 8.0). The eluate was mixed with 30 μl 5x Laemmli sample buffer and heated for 10 min at 95 °C. Eluted proteins (30 µl) were analyzed by SDS-PAGE. A band observed only in the sample obtained from FILIP1-2 CT-coated beads incubated with cell extract was cut from the gel. For protein identification, a tryptic digest was prepared and analyzed by LC-MS using a Q Exactive Plus system and a 1 h LC gradient.

For FLNc d18–21 and d1–3 pull-down experiments, FLNc-His variants immobilized on Ni^2+^-NTA beads were purified as described above and used to pull-down putative binding partners from C2 myotube lysates. Differentiated myotubes (1xT74 flask) were lysed in 1 ml lysis buffer [25 mM Tris, 150 mM NaCl, 0.5% (v/v) sodiumdeoxycholate, 1% (v/v) Nonidet P-40, 10% (v/v) glycerol, 2 mM EDTA and a protease inhibitor cocktail tablet, pH 8.0]. Lysates were sonicated for 10 s and insoluble material was removed by centrifugation (10 min, 21,000 *g*). Cleared lysates were incubated while rotating at 10 °C for 1 h with empty Ni^2+^-NTA-agarose beads or beads with bound FLNc d1–3 or FLNc d18–21. After five times washing with lysis buffer, bound proteins were eluted from the beads by addition of 100 µl 1x Laemmli sample buffer and heated for 10 min at 95 °C. Eluted proteins (20 µl) were analyzed by SDS-PAGE. At around 130 kDa, a band from each lane was cut, subjected to in-gel protein digestion using trypsin and peptide extracts were analyzed by LC-MS on a Q Exactive Plus system with a 1 h LC gradient.

### Co-immunoprecipitation and in vitro binding assays

HEK293 cells were co-transfected with FILIP1-GFP CT and Myc-FLNc d1-4, Myc-FLNc d18–21 or the corresponding FLNc phosphosite mutant forms. Forty-eight hours after transfection, cells were lysed on ice using co-immunoprecipitation (co-IP) lysis buffer [100 mM NaCl, 20 mM Tris, 1% Triton X-100, 1 mM sodium orthovanadate, 10 mM β-glycerophosphate, 9.5 mM sodium fluoride, 10 mM sodium pyrophosphate, protease inhibitor cocktail tablet (Roche Diagnostics, Mannheim, Germany), pH 7.6]. Lysates were centrifuged at 10,000 *g* for 5 min. 800 μl of each supernatant were incubated with 10 μl anti-c-Myc Magnetic Beads (Thermo Fisher Scientific) at 10 °C for 3 h on a rotating wheel. Samples were washed three times with 300 μl co-IP lysis buffer using a magnetic rack, and bead-bound proteins were resuspended in 50 μl Laemmli sample buffer for Western blot analysis. Bacterial expression and purification of recombinant protein fragments of FILIP1-2 or FILIP1-4 (T7-tagged) and FLNc and FLNa (EEF-tagged) for co-immunoprecipitation and dot-blot overlay experiments were performed as described above in the section ‘Yeast two-hybrid and pull-down assays’. Purified FILIP protein fragments were spotted on a PBS pre-conditioned nitrocellulose membrane and air-dried. The membrane was blocked (1% bovine serum albumin, 0.2% Tween-20, 100 mM KCl, 20 mM imidazole-HCl pH 7.0, 0.1 mM DTT) for 30 min and treated with the respective purified FLNc protein fragments in the same buffer. Three washing steps were followed by a 45 min incubation step with the YL1/2 antibody directed against the EEF-tag. Peroxidase-conjugated goat anti-rat secondary antibodies (Dianova) were used to visualize signals by enhanced chemiluminesence.

### Fluorescence correlation spectroscopy

HEK293T cells co-expressing either FILIP1 CT-GFP and FLNc d18–21 or FILIP1 CT-GFP and FLNc d18–21 DD mutant were washed in PBS and lysed in ice-cold 0.1% Tween20/PBS for 1 min and centrifuged at 3,000 *g* for 15 s. The supernatant was used immediately for binding studies. FCS was performed with an instrumental setup described previously^63^. A ConfoCor 2 spectrofluorimeter (Carl Zeiss-Evotec, Germany) equipped with an air-cooled Argon-laser (LASOS Lasertech GmbH, Germany; intensity 70 μW) and a water immersion objective (C-Apochromat 63 ×/1.2 W Corr) was used for monitoring competition interactions. The focus in the z-direction was set 150 μm over the cover glass of a 1536-well plate (Greiner Bio-One, Germany) to record diffusion of fluorescent particles through the focal element. The diameter of the pinhole was set to 35 μm and the confocal volume was calibrated using DyLight 488 dye (Dtrans = 4.0 × 10^−10^ m^2^ s^−1^). All FCS measurements were performed in PBS (pH 7.4) supplemented with 0.05% Tween-20 in a sample volume of 10 μl and at 20 °C. For the competition assay various concentrations from 44,000 nM to 21 nM of FLNc d19–21 or of 60,000–78 nM FLNc d19–21 DD mutant, respectively, were titrated to a fix volume of lysate comprising FILIP1 CT-GFP bound to wild-type FLNc d18–21 or phosphomimetic DD mutant. Intensity fluctuations were recorded by an avalanche photodiode (SPCM-CD 3017) in photon counting mode over a time period of 20 s and autocorrelated with a hardware correlator (ALV 5000, ALV, Germany). Evaluation of the autocorrelated curves was performed with the FCS ACCESS Fit (Carl Zeiss-Evotec, Germany) software package using a Marquardt nonlinear least-squares algorithm for a one-component fitting model.

### Fluorescence recovery after photobleaching

Fluorescence recovery after photobleaching (FRAP) experiments were performed and analyzed essentially as described previously^9,17^. In brief, experiments were performed on myotubes derived from immortalized mouse skeletal muscle cells^64^ expressing full-length hFLNc-EGFP and differentiated for 6 days, using a Cell Observer SD microscope (Carl Zeiss, Jena, Germany) with a Plan-Apochromat 63×/1.4 oil objective and an external 474 nm laser. During the experiments the cells were constantly kept at 37 °C and 5% CO_2_. In each myotube, 1–3 regions of interest (ROIs, single Z-discs) were bleached. ROIs were photographed before bleaching, immediately after bleaching as well as 5, 20, 60 and 120 s after bleaching. Mean halftimes were calculated on the basis of exchange process of bound protein with the soluble fraction in the slower phase of the biphasic recovery profile. Mobile fractions are calculated based on the recovery of the fluorescence in comparison to the initial starting intensity in the ROIs.

### Transient siRNA-mediated knockdown of FILIP1

C2 or C2C12 myoblasts were transiently transfected with FILIP1 siRNA (#NM_001081243: SASI_Mm02_00296577, MISSION, Sigma Aldrich) or control siRNA (#SIC002, Sigma Aldrich) in a final concentration of 10 nmol using RNAiMax reagent (ThermoFisher Scientific) according to the manufacturer´s protocol. Six hours after transfection, cell culture medium was changed and, after further 18 h, differentiation of cells was induced as described above. At specific time points after transfection, cells were lysed for Western Blot analysis or analyzed by fluorescence microscopy. For western blot analysis, cells were lysed using RIPA buffer and protein concentrations were equalized using the Bradford assay.

### Immunofluorescence staining of cells

C2C12 cells were fixed with 4% paraformaldehyde in PBS for 10 min and permeabilized for 10 min with 0.5% Triton X-100 in PBS. Fixed cells were incubated with T12 anti-titin antibody and Alexa-594-conjugated goat-anti-mouse IgG1 secondary antibodies. Cell nuclei were stained by adding DAPI to the secondary antibodies. Cells were embedded with Fluoromount-G mounting medium (ThermoFisher Scientific) and analyzed with an LSM710 confocal laser scanning microscope (Carl Zeiss, Jena, Germany).

### Ubiquitination assay

C2 cells, seeded on 10 cm culture dishes, were co-transfected with HA-tagged full-length FILIP1 and Myc-FLNc d18–21 or the corresponding FLNc phosphosite mutant variants. Eighteen hours after transfection, cells were treated for 6 h with 10 µM of MG-132. Twenty-four hours after transfection, cells were lysed on ice with 1 ml modified RIPA buffer supplemented with a protease inhibitor cocktail tablet (Roche Diagnostics, Mannheim, Germany). Lysates were sonicated for 30 s and centrifuged at 10,000 *g* for 5 min. Enrichment of the Myc-tagged FLNc fragment was performed with 20 µl Myc-Dynabeads (Invitrogen) according to the manufacturer’s protocol. Samples were eluted by addition of Laemmli sample buffer at 95 °C and analyzed via SDS-PAGE and subsequent Western blot analysis.

### Site-directed mutagenesis

Cloning of the full-length hFLNc cDNA in pEGFP vectors was described before^65^. Briefly, a fragment encoding domains 15-24 was amplified by PCR from a human skeletal muscle cDNA library (Clontech) and cloned into a pEGFP-N3 vector. Subsequently, a fragment encoding ABD-d15 was amplified using a FLNC cDNA as a template (HP07616-ARi57A02, deposited by Seishi Kato, Research Institute of National Rehabilitation Center for Persons with Disabilities and provided by the RIKEN BioResource Center) and cloned via BclI restriction site in the partial FLNC clone to obtain a full-length construct. Site-directed mutagenesis of full-length FLNc was performed using the QuikChange® Lightning Site-Directed Mutagenesis Kit (Stratagene/Agilent Technologies, Waldbronn, Germany) as described in the manual. AA (S2233/S2236 to alanine) and DD (S2233/S2236 to aspartate) mutants were obtained by PCR using the primer pairs (5′–3′) GGGATCCTTCGGCGCCATCACCCGGCAG with CTGCCGGGTCATGGCGCCGAAGGATCCC, and TGGGATCCTTCGGCGACATCACCCGGCAGC with GCTGCCGGGTGATGTCGCCGAAGGATCCCA, respectively, using the non-mutant variant as template. All other constructs used in this work were obtained by cloning PCR products amplified with primers including restriction sites. Amplicons were cloned in the appropriate vectors and used for expression in immortalized mouse myoblasts, HEK293 or L40 yeast cells, or in *E. coli*. AA and DD variants of hFLNC d18–21 in pET23a/EEF and pcDNA3.1/Myc-His were obtained by amplifying the mutated cDNA fragment from the full-length mutant constructs. The AS (S2233 to A), DS (S2233 to D), SA (S2236 to A) and SD (S2236 to D) mutants of hFLNC d18–21 in pET23a/EEF and pcDNA3.1/Myc-His were obtained by single strand site-directed mutagenesis. Single strand PCR of the non-mutant plasmid was performed using primer pairs CTGGGAGCCTTCGGCAG, GATGCTGCCGAAGGCTCCCAG and CCTGGGAGACTTCGGCAG, CTGCCGAAGTCTCCCAGGCG for the AS mutant and DS mutant, respectively. For the generation of the SA and SD mutants, primer pairs CCTTCGGCGCCATCACCCG, CCTTCGGCGCCATCACCCG and CCTTCGGCGACATCACCCG, CGGGTGATGTCGCCGAAGG were used, respectively. Complementary PCR products were mixed and incubated for 5 min at 98 °C. PCR mixtures were purified using the mi-PCR Purification Kit (Metabion International AG, Planegg/Steinkirchen, Germany). To remove template DNA, samples were digested using DpnI and transformed into *E. coli* (DH5 alpha strain).

### Antibodies

Anti-Akt pan (1:1000, #4691), anti-Akt-pT308 (1:1000, #2965), anti-Akt-pS473 (1:1000, #4060), anti-eEF2 (1:1000, #2332), anti-eEF2-T56 (1:1000, #2331), anti-eIF4B (1:1000, #3592), anti-eIF4B-pS406 (1:1000, #5399), anti-GAPDH (1:1000, #2118), anti-GSK-3β (1:1000, #12456), anti-GSK-3α/β-pS21/9 (1:1000, #8566), anti-p70S6K (1:1000, #2708), anti-p70S6K-pT389 (1:1000, #9206), anti-phospho serine PKC substrate (1:1000, #2261), anti-Rictor (1:1000, #9476), anti-PKCα (1:1000, #2056), anti-p44/42 MAPK (ERK1/2) (1:1000, #4695), anti-p44/42 MAPK (ERK1/2)-pT202/pY204 (1:1000, #4370) and anti-Rictor-pT1135 (1:1000, #3806) were purchased from Cell Signaling Technology (Leiden, the Netherlands). Anti-PKCα-pS657 (1:1000, ab180848) was purchased from Abcam (Cambridge, UK). Anti-His (1:3000, H1029) and anti-myc (1:2000, #60003-2-Ig) antibodies were purchased from Sigma Aldrich and Proteintech (Manchester, UK), respectively. Anti-HA (1:2000, 51064-2-AP), Anti-Bag3 (1:1000, 10599-1-AP) and anti-LC3 antibody (1:1000, 14600-1-AP) were purchased from Proteintech. Anti-Ubiquitin monoclonal antibody (P4D1) was purchased from Enzo Life Sciences (1:1000, BML-PW0930-0100). FLNc antibody RR90 was used in 1:100 dilution as described before^26^, anti-FLNc-pS2233 antibody was purchased from Kinasource Limited (0.2 µg/ml, Dundee, UK). Another FLNc polyclonal antibody^66^ was raised against recombinantly expressed FLNc d16–20 (1:50,000 BioGenes, Berlin, Germany). FLNa polyclonal antibody^67^ was raised against recombinantly expressed FLNa d16–20 (1:50,000, BioGenes). Alpha-tubulin and proteins with an EEF-tag were detected using monoclonal antibody YL1/2, diluted 1:2000^68^. A novel rabbit antiserum against FILIP1 was raised against the recombinantly expressed carboxy-terminus of FILIP1-2 (BioGenes, Berlin, Germany). The serum was affinity-purified against the antigen and preabsorbed against the carboxy-terminus of the highly homologous FILIP1L to avoid cross-reactivity and used in 1:250 dilution. Horseradish peroxidase (HRP)-conjugated anti-rabbit, anti-mouse, anti-rat and anti-sheep immunoglobulins were used in 1:10,000 dilution if not otherwise stated and purchased from Sigma-Aldrich, Thermo and Dianova (Hamburg, Germany), respectively.

### Statistics and reproducibility

Information about the experimental design and statistical rationale for the different analyses performed in this work are provided within the respective subsections in the results. Unless otherwise stated, boxes generally represent the median as a line surrounded by a box, which represents the 25-75 percentiles of the data. Whiskers range within the 5-95 percentiles. Numbers of sample size, replicates, controls, and statistical tests were chosen according to published data with comparable methodology and generally accepted standards. SDS-PAGE and Western blotting were performed using standard protocols; signals were detected using HRP-coupled secondary antibodies and an enhanced chemiluminescence system (Thermo Fisher Scientific) either with a ChemoCam (Intas, Göttingen, Germany) equipped with a full-frame 3.2 megapixel Kodak KAF-3200ME camera, or by exposure to an X-ray film (Fujifilm, Minato, Japan) which was developed subsequently (Curix 60, AGFA, Mortsel, Belgium). No image processing, other than cropping, scaling and contrast adjustment, was applied. Quantification of Western blot signals was performed with Quantity One 4.6.9 (Bio-Rad, Hercules, CA, USA). For statistical analysis, paired two-sided *t*-tests were performed using OriginPro 9.1 (OriginLab, Northampton, MA, USA). All quantitative Western blot data are presented as mean ± SEM or standard deviation (SD). To minimize the effects of subjective bias, Western blot data were generated and analyzed by two different experimenters.

### Reporting summary

Further information on research design is available in the Nature Research Reporting Summary linked to this article.

## Supplementary information

Supplementary Data 1

Supplementary Data 2

Supplementary Data 3

Supplementary Data 4

Supplementary Data 5

Supplementary Data 6

Supplementary Data 7

Supplementary Data 8

Supplementary Data 9

Supplementary Data 10

Supplementary Data 11

Supplementary Data 12

Supplementary Data 13

Supplementary Information

Description of Additional Supplementary Files

Reporting Summary

Peer Review File

## Data Availability

All raw data and original MaxQuant result files have been deposited to the ProteomeXchange Consortium (http://proteomecentral.proteomexchange.org) via the PRIDE partner repository^69^ with the dataset identifiers PXD009117 (large-scale quantitative analysis with DMSO, LY, IGF-1), PXD016721 (quantitative EasyPhos analysis with IGF-1, IGF-1+LY, IGF-1+MK), PXD008893 (in vitro kinase assay), PXD009228 (targeted PRM assay), PXD009159 (BioID experiments) PXD017670 (FilaminC pull-down experiment from myotube lysates) and PXD008875 (FILIP1 pull-down experiment). Processed data of in vitro and in cellulo kinase assays (https://panoramaweb.org/FLNc_d18-21_ivka_AKT_PKCa.url) and PRM assays (https://panoramaweb.org/FLNc_S2233_S2236_PRM.url) analyzed with Skyline and their results are available on PanoramaWeb interface^70^. Uncropped images of Western blots, sequence alignments, constructs used for cell transfection and bacterial transformation as well as bright field and fluorescence microscopic pictures are shown in Supplementary Figs. 2, 5, 7, 10, 11 and 12. Molecular mass markers and the outlines of cropping presented in the main figures are indicated.
