# Supplementary material for: Phosphoproteomics identifies dual-site phosphorylation in an extended basophilic motif regulating FILIP1-mediated degradation of filamin-C
Source: Commun Biol. 2020 May 22;3:253. doi: 10.1038/s42003-020-0982-5 (PMC7244511; doi:10.1038/s42003-020-0982-5)
Supplement: Supplementary file 14 — Supplementary Information [file 42003_2020_982_MOESM14_ESM.pdf]

Supplementary Figure 1

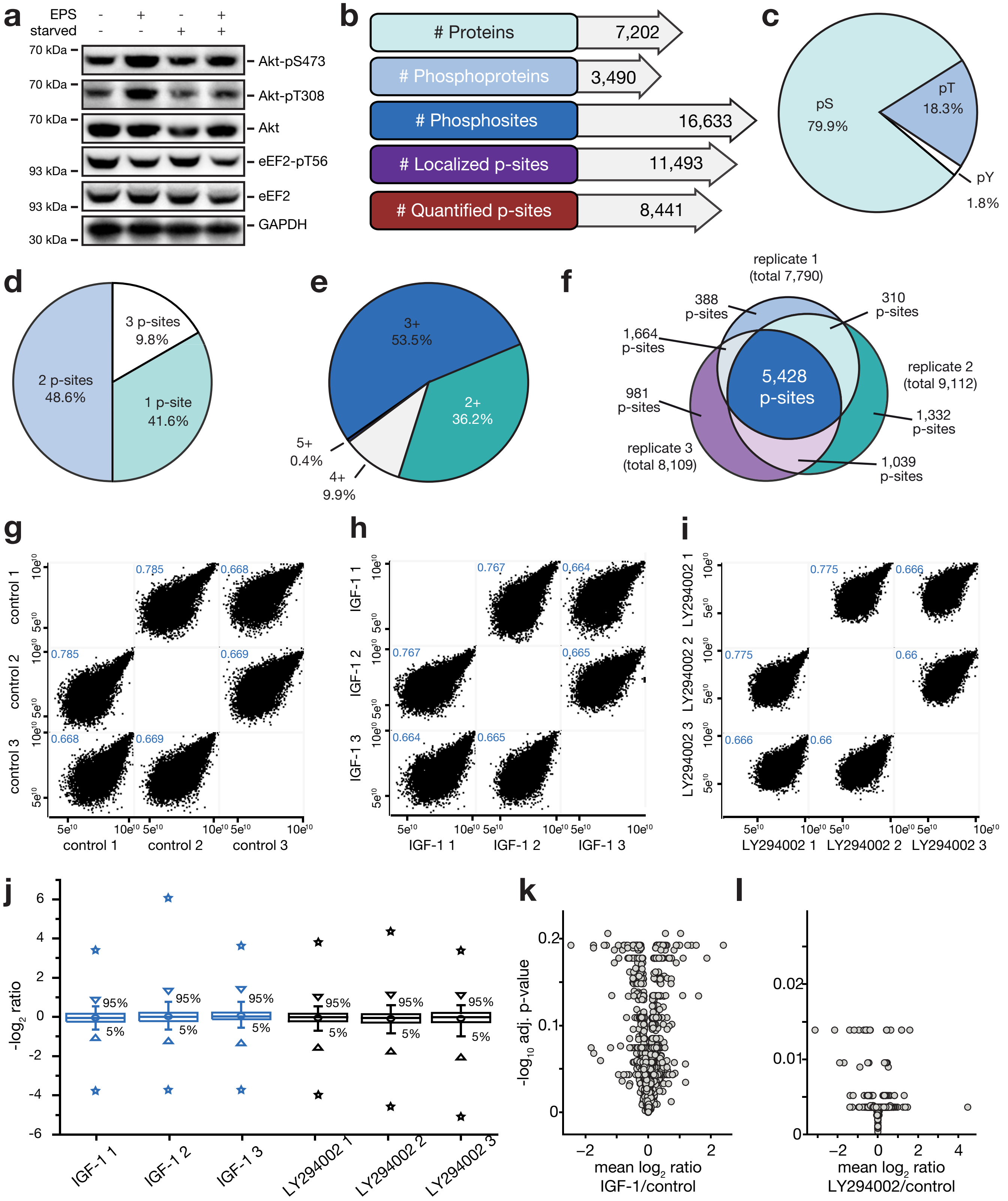

Supplementary Figure 1. Evaluation of quantitative phosphoproteome data from mouse skeletal myotubes follwoing treatment with IGF-1 or LY294002. **a.** C2 myotubes were starved over night and treated with EPS for 4 h. Lysates were subjected to SDS-PAGE and immunoblot analysis using total and phospho-specific antibodies. **b.** Overview of the number of identified proteins and phosphoproteins as well as the number of counted, localized (≥ 0.75) and quantified phosphosites obtained by SILAC-MS and MaxQuant data analysis. **c.** Distribution of peptides identified to be phosphorylated at serine (pS) threonine (pT) or tyrosine (pY). **d.** Distribution of singly, doubly and triply phosphorylated peptides. **e.** Distribution of the charge state of phosphopeptides. **f.** Overlap of localized and quantified phosphosites between independent experiments. In sum, 8,441 phosphosites (73%) of all localized sites were quantified in two out of three replicates. **g-i.** Multi-scatter plots with Pearson correlation analysis between biological replicates for each condition (i.e. control, IGF-1 and LY294002 treatment). **j.** Box-plot analysis of log_2-transformed phosphopeptide ratios in replicates 1-3. Only a small fraction of all quantified phosphopeptides exhibits a log_2 ratio ≥ 0.584 or ≤ -0.584 (i.e. minimum fold-change of 1.5). **k and l.** Scatter plots of quantified proteins after 1 h treatment with IGF-1 (k) and LY294002 (l) in comparison to DMSO-treated control. No changes in the relative abundance of proteins were found (n=3 independent experiments; two-tailed moderated student's t-test).

Supplementary Figure 2

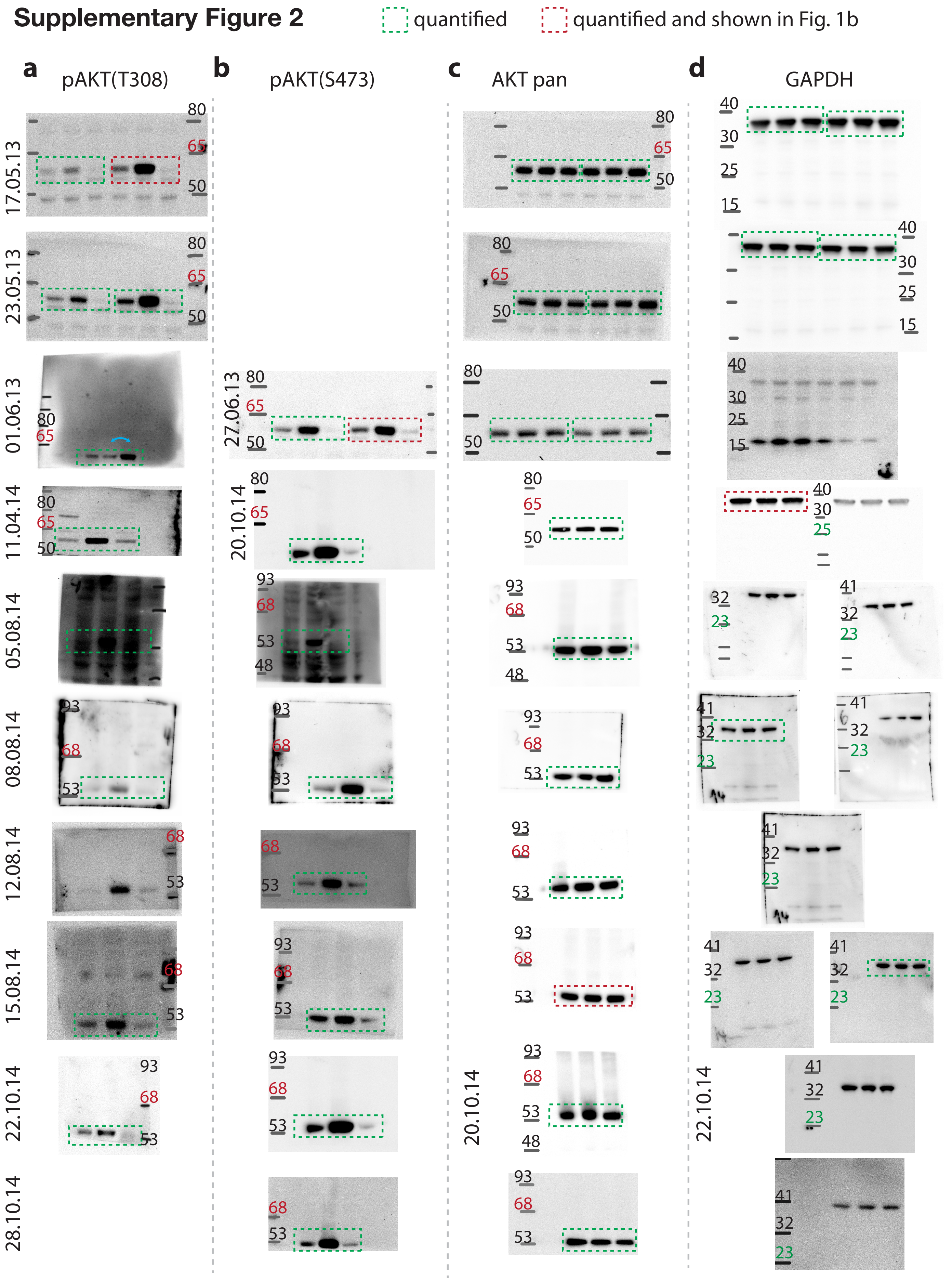

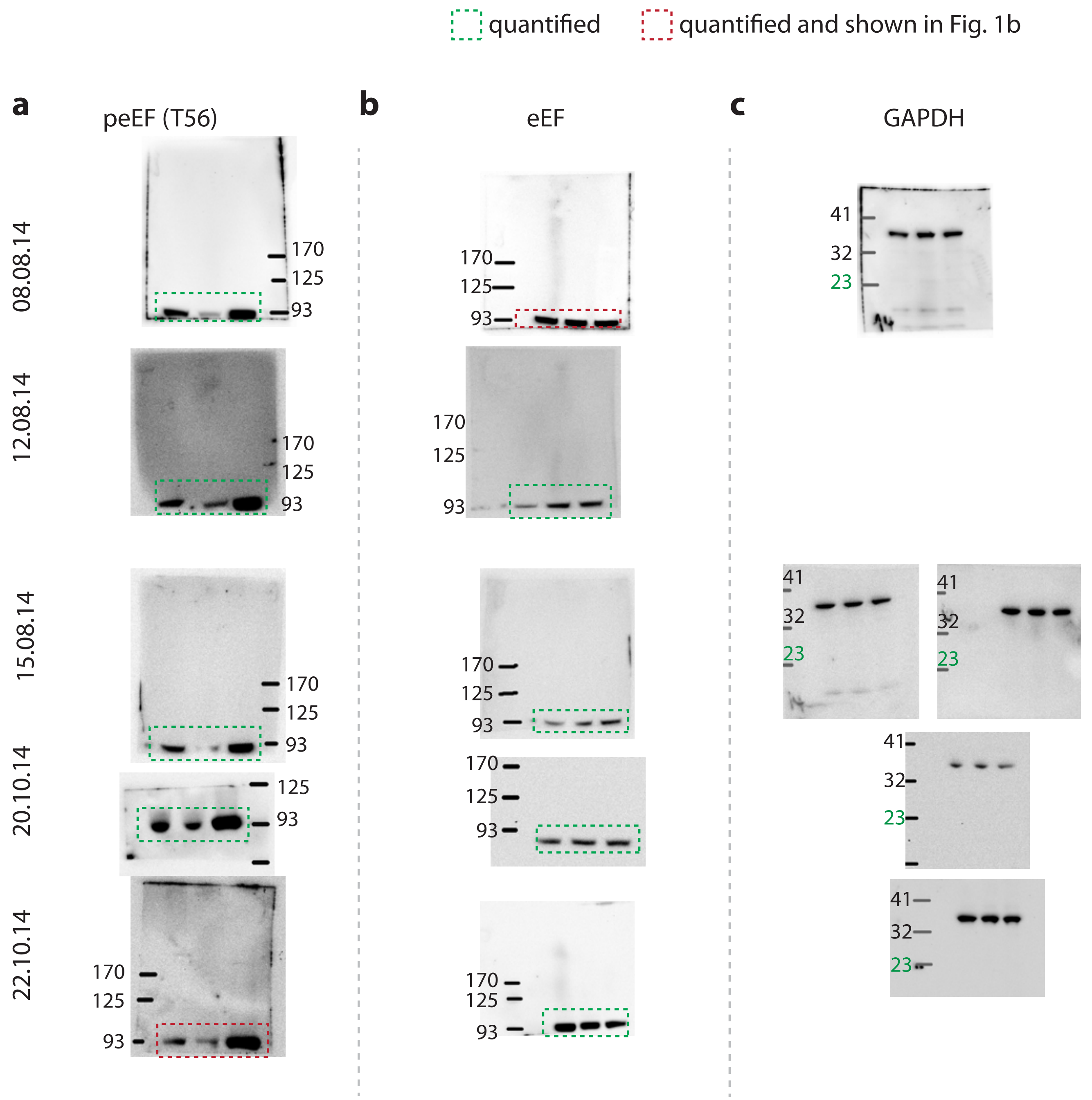

quantified
quantified and shown in Fig. 1b
a
peEF (T56)
b
eEF
c
GAPDH
08.08.14
12.08.14
15.08.14
20.10.14
22.10.14
170
125
93
41
32
23

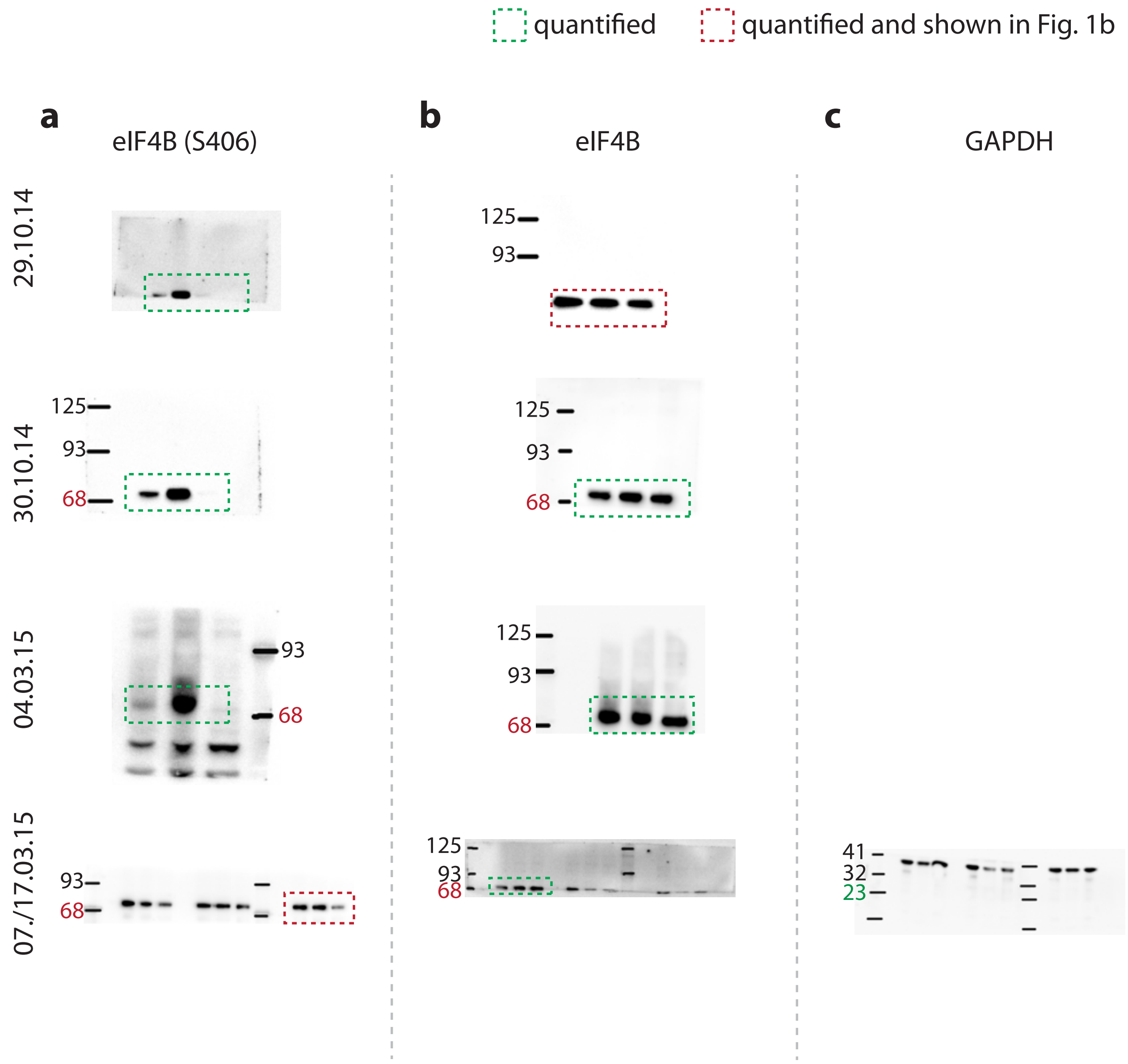
quantified
quantified and shown in Fig. 1b
a
eIF4B (S406)
29.10.14
30.10.14
04.03.15
07./17.03.15
b
eIF4B
c
GAPDH
125
93
68
41
32
23

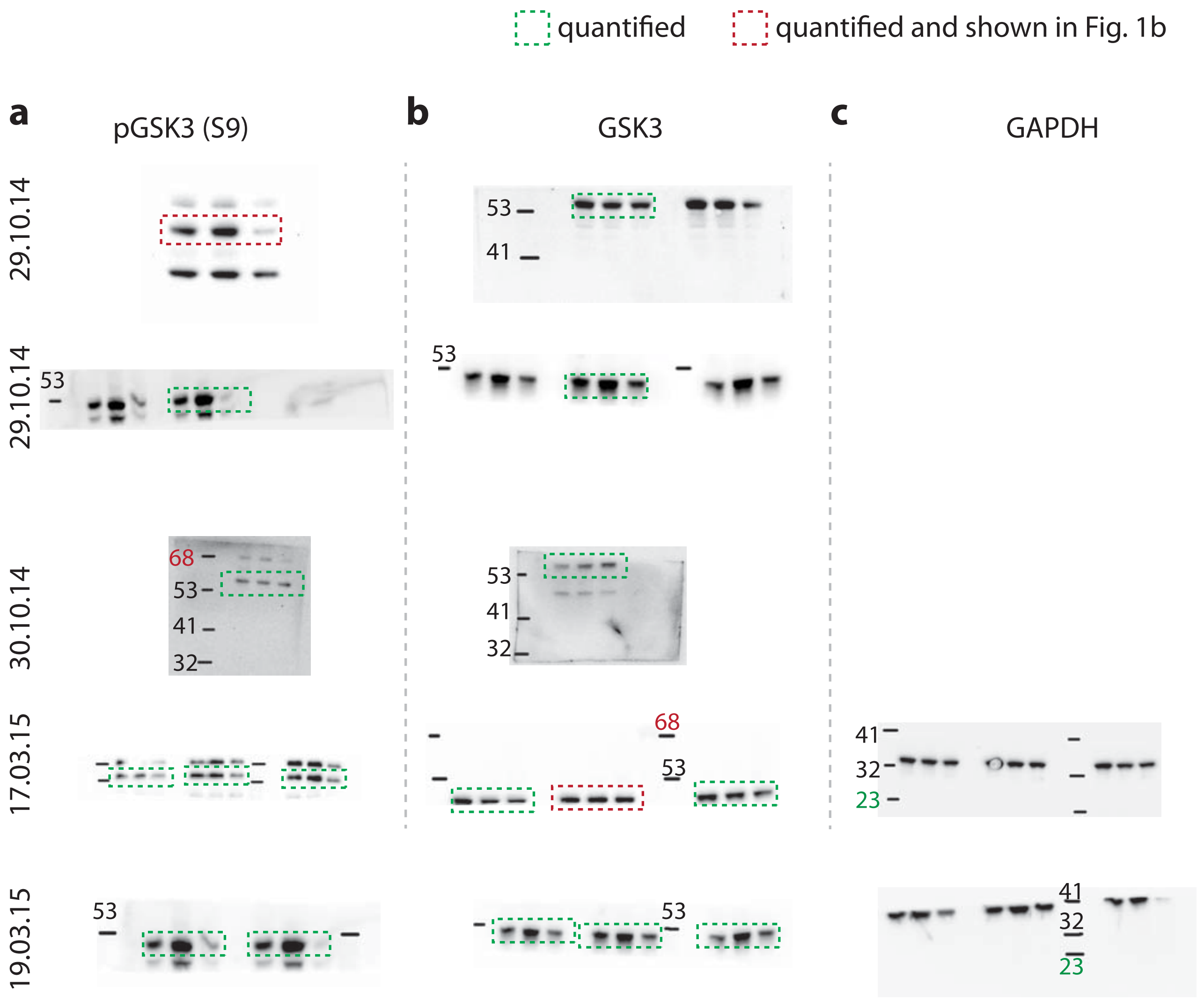
quantified
quantified and shown in Fig. 1b
a
pGSK3 (S9)
b
GSK3
c
GAPDH
29.10.14
29.10.14
30.10.14
17.03.15
19.03.15
53
41
68
32
23

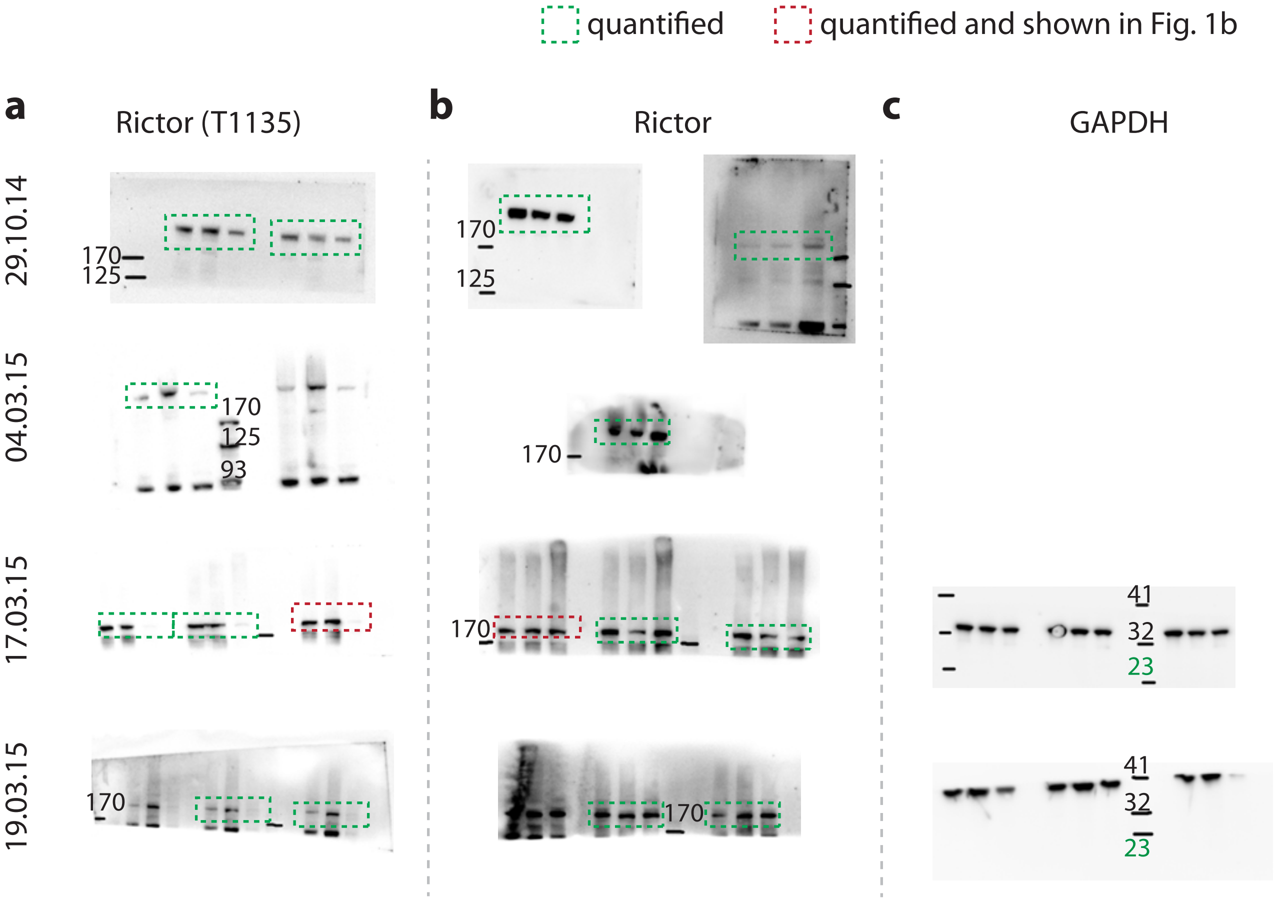

quantified
quantified and shown in Fig. 1b
a
Rictor (T1135)
b
Rictor
c
GAPDH
29.10.14
04.03.15
17.03.15
19.03.15
170
125
93
41
32
23

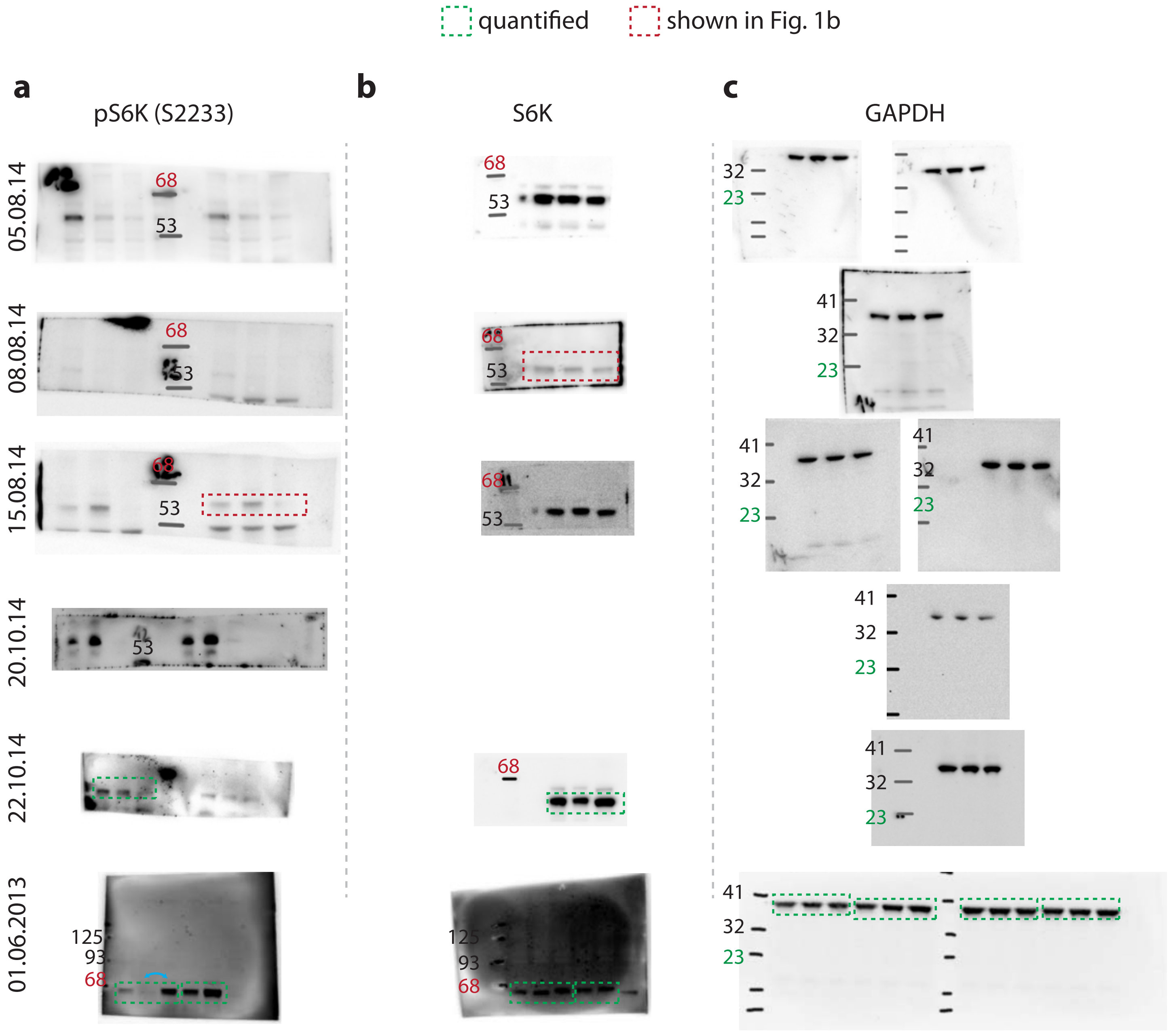

quantified
shown in Fig. 1b
a
pS6K (S2233)
b
S6K
c
GAPDH
05.08.14
08.08.14
15.08.14
20.10.14
22.10.14
01.06.2013
125
93
68
53
41
32
23

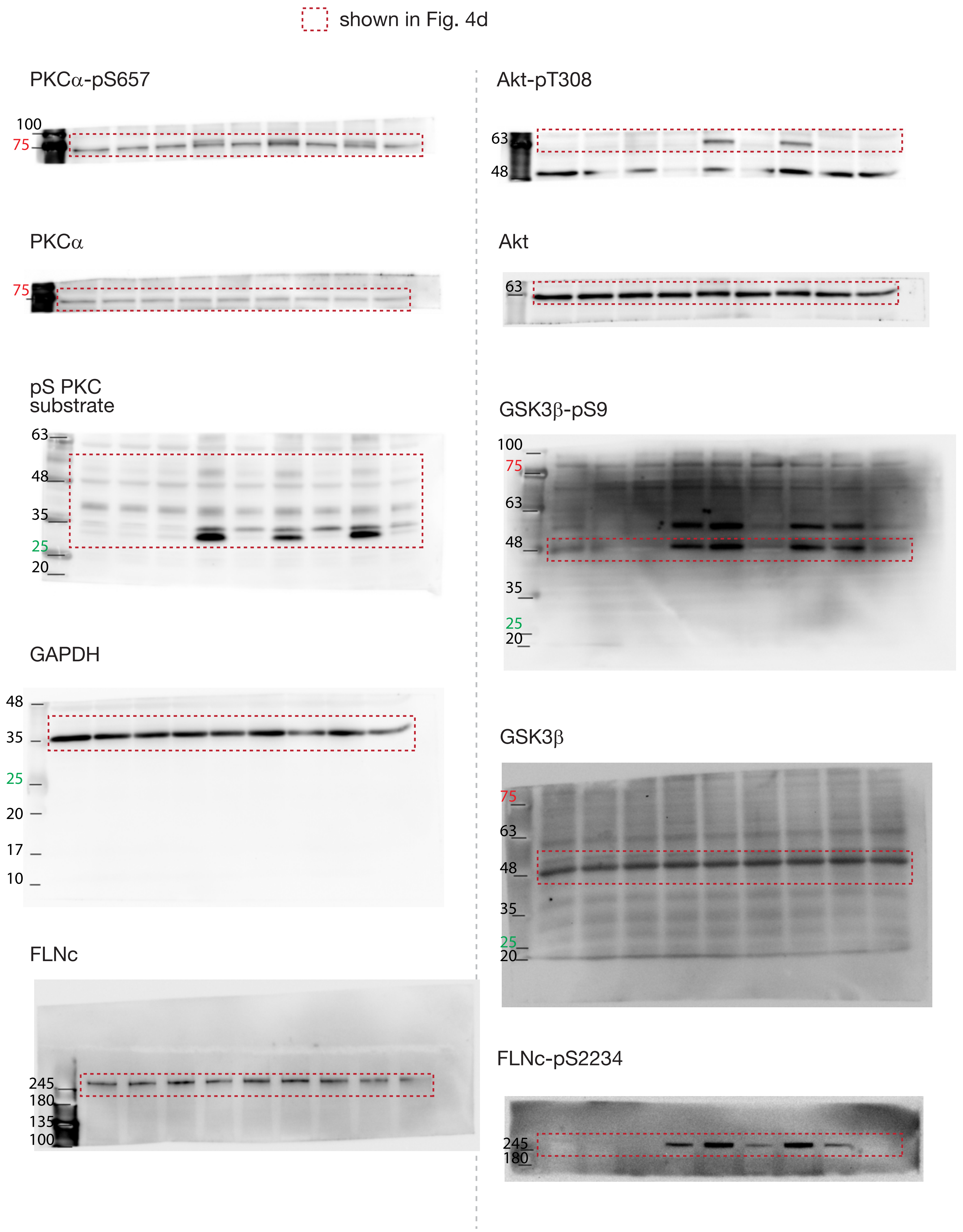
shown in Fig. 4d
PKCα-pS657
100
75
PKCα
75
pS PKC substrate
63
48
35
25
20
GAPDH
48
35
25
20
17
10
FLNc
245
180
135
100
Akt-pT308
63
48
Akt
63
GSK3β-pS9
100
75
63
48
35
25
20
GSK3β
75
63
48
35
25
20
FLNc-pS2234
245
180

shown in Fig. 5e

His

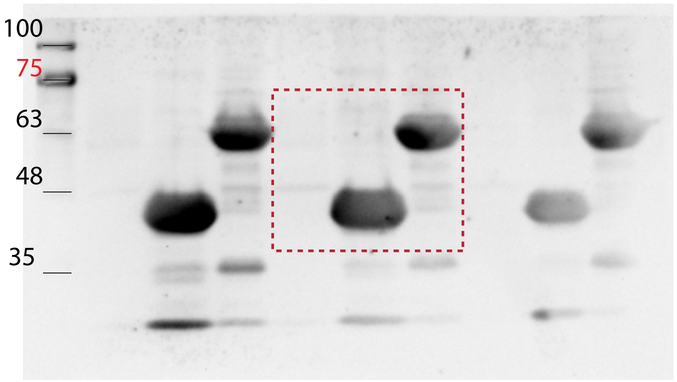

FILIP1

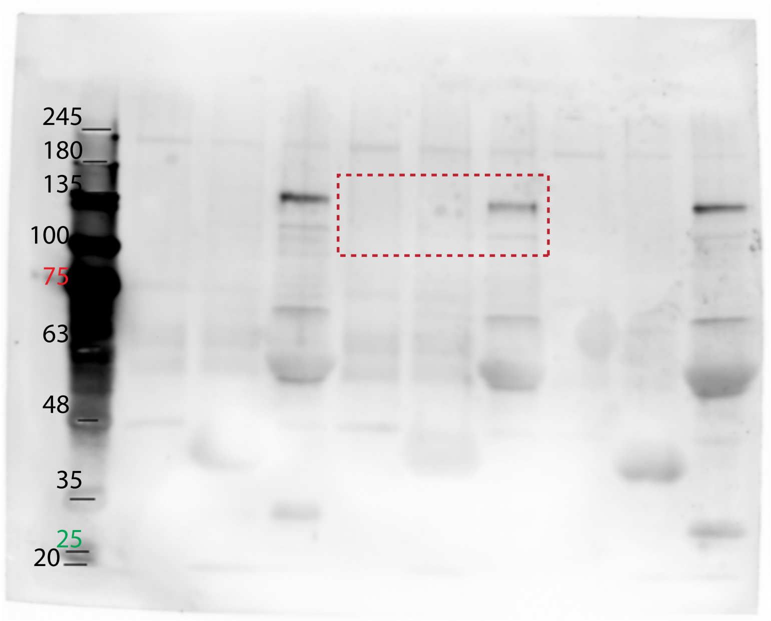

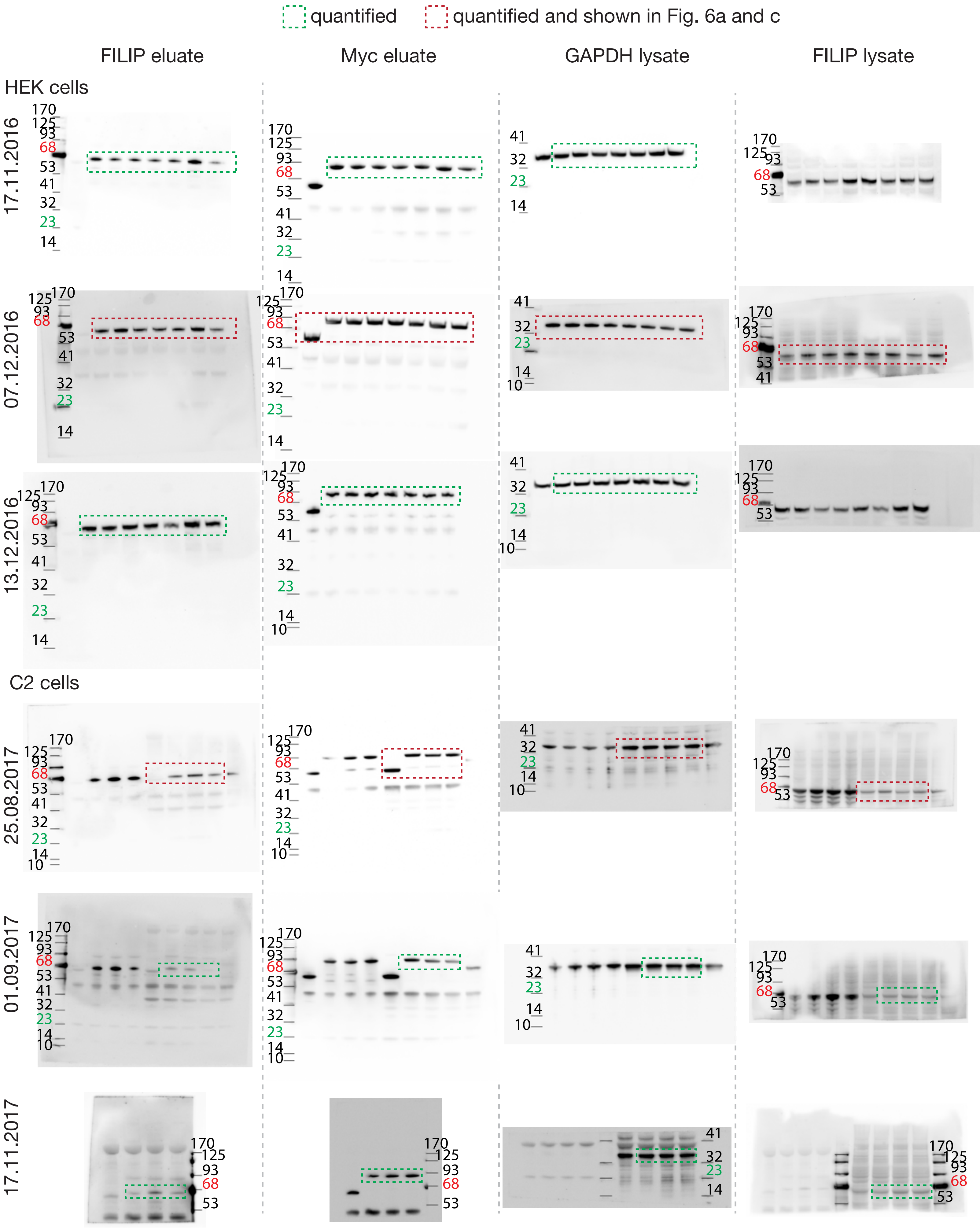

quantified
quantified and shown in Fig. 6a and c
FILIP eluate
Myc eluate
GAPDH lysate
FILIP lysate
HEK cells
17.11.2016
07.12.2016
13.12.2016
C2 cells
25.08.2017
01.09.2017
17.11.2017
170
125
93
68
53
41
32
23
14
10

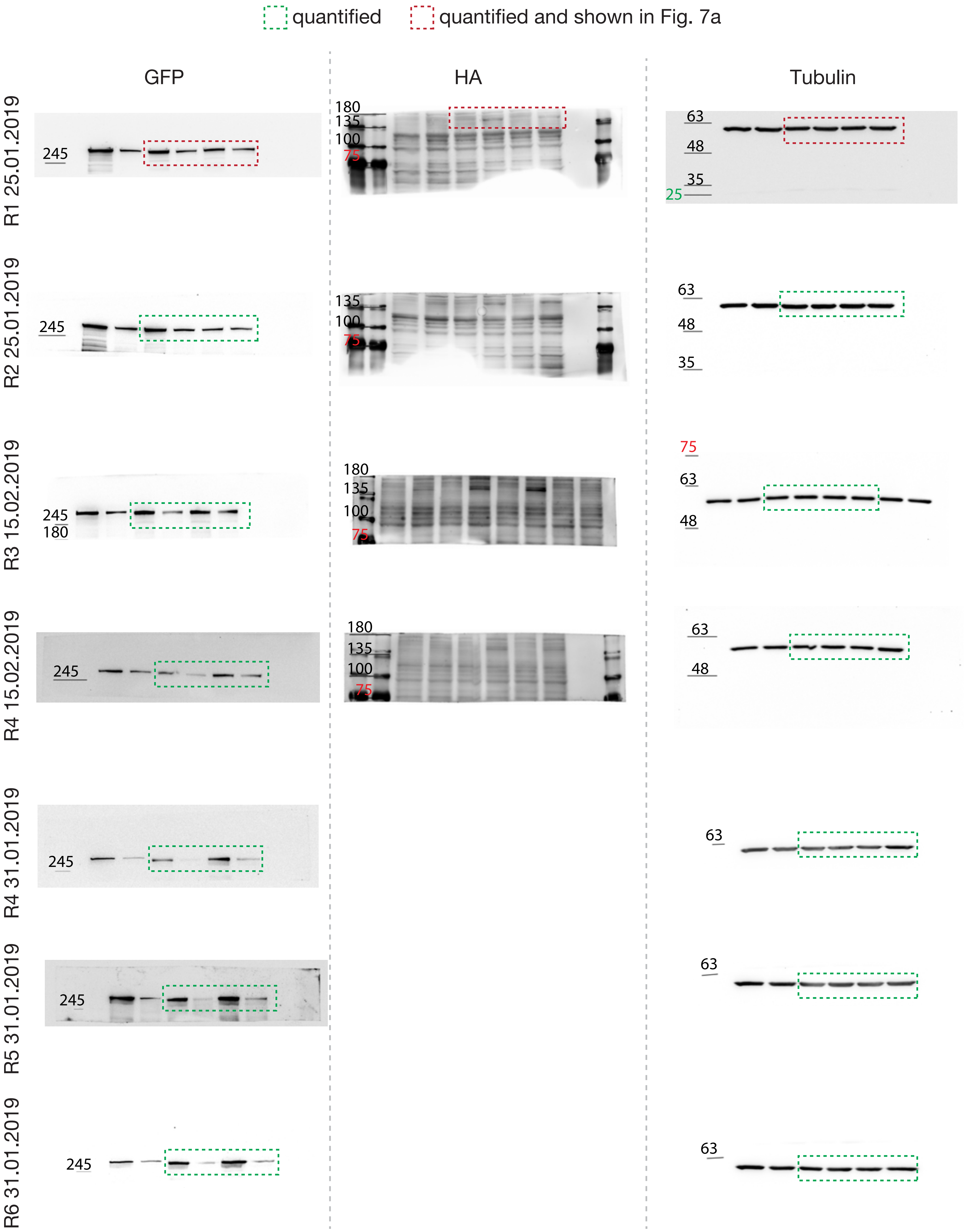
quantified
quantified and shown in Fig. 7a
GFP
HA
Tubulin
R1 25.01.2019
R2 25.01.2019
R3 15.02.2019
R4 15.02.2019
R4 31.01.2019
R5 31.01.2019
R6 31.01.2019
245
180
180
135
100
75
63
48
35
25

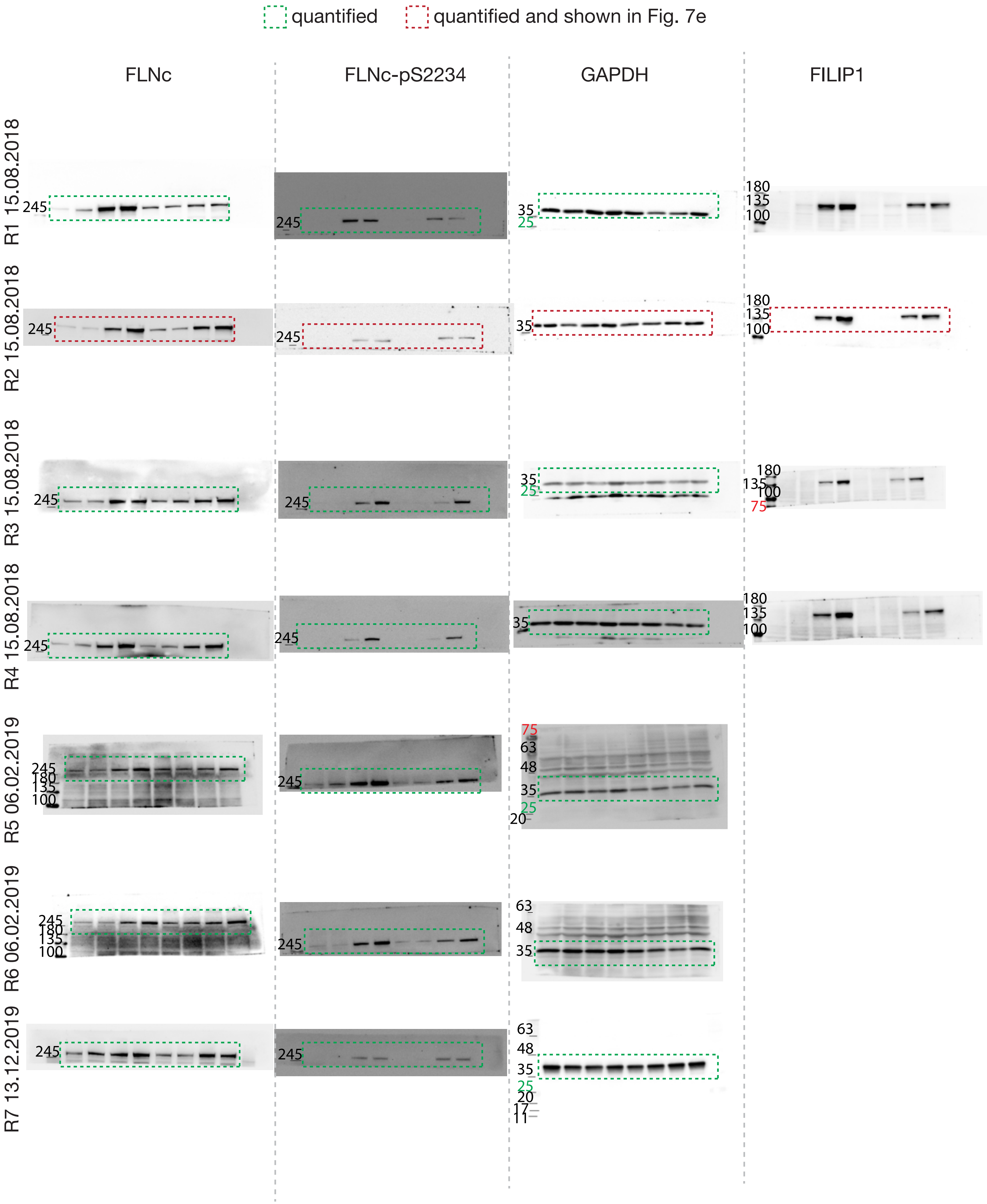
quantified
quantified and shown in Fig. 7e
FLNc
FLNc-pS2234
GAPDH
FILIP1
R1 15.08.2018
R2 15.08.2018
R3 15.08.2018
R4 15.08.2018
R5 06.02.2019
R6 06.02.2019
R7 13.12.2019
245
35
25
180
135
100
75
63
48
20
17
11

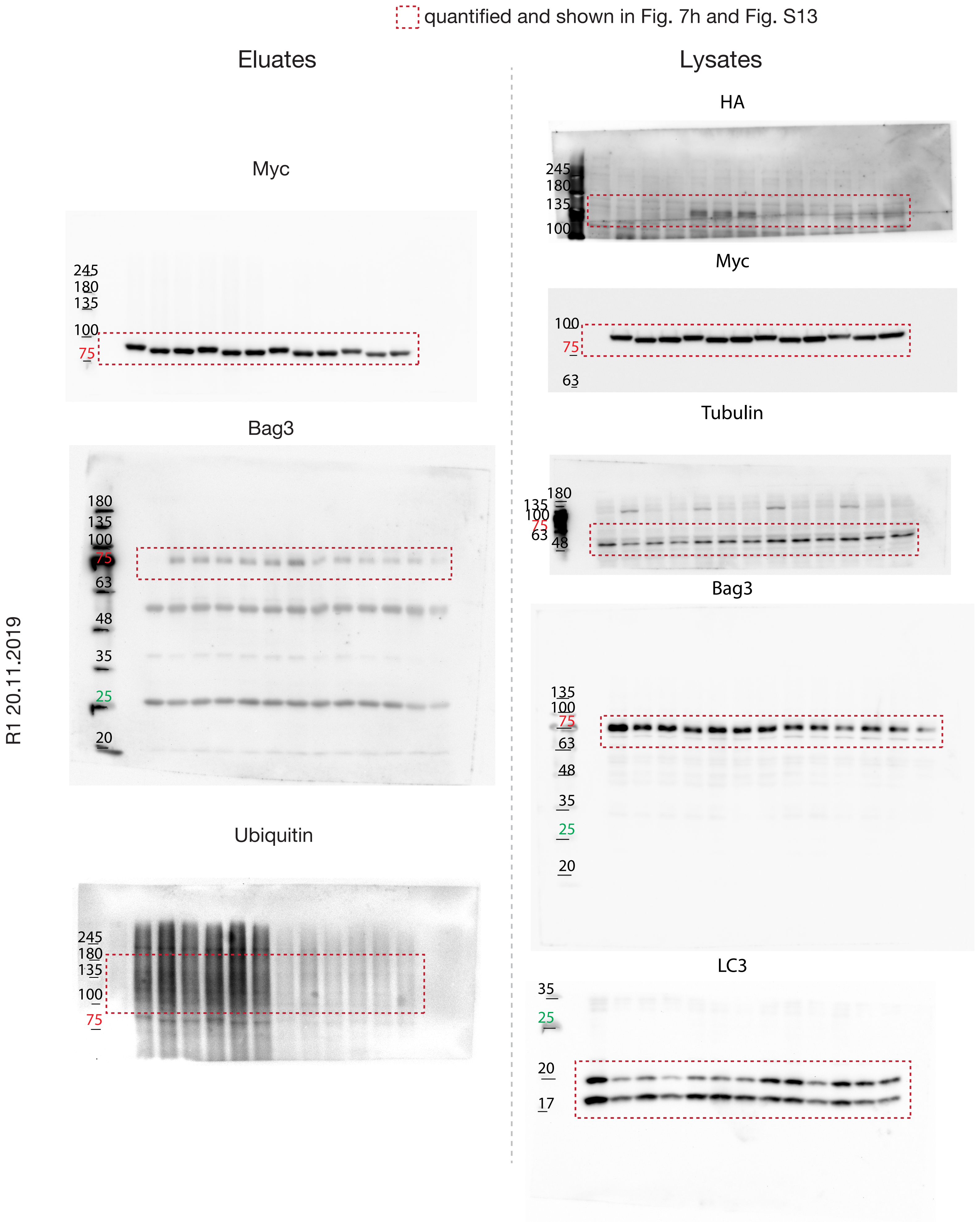
quantified and shown in Fig. 7h and Fig. S13
Eluates
Lysates
R1 20.11.2019
Myc
245
180
135
100
75
Bag3
180
135
100
75
63
48
35
25
20
Ubiquitin
245
180
135
100
75
HA
245
180
135
100
Myc
100
75
63
Tubulin
180
135
100
75
63
48
Bag3
135
100
75
63
48
35
25
20
LC3
35
25
20
17

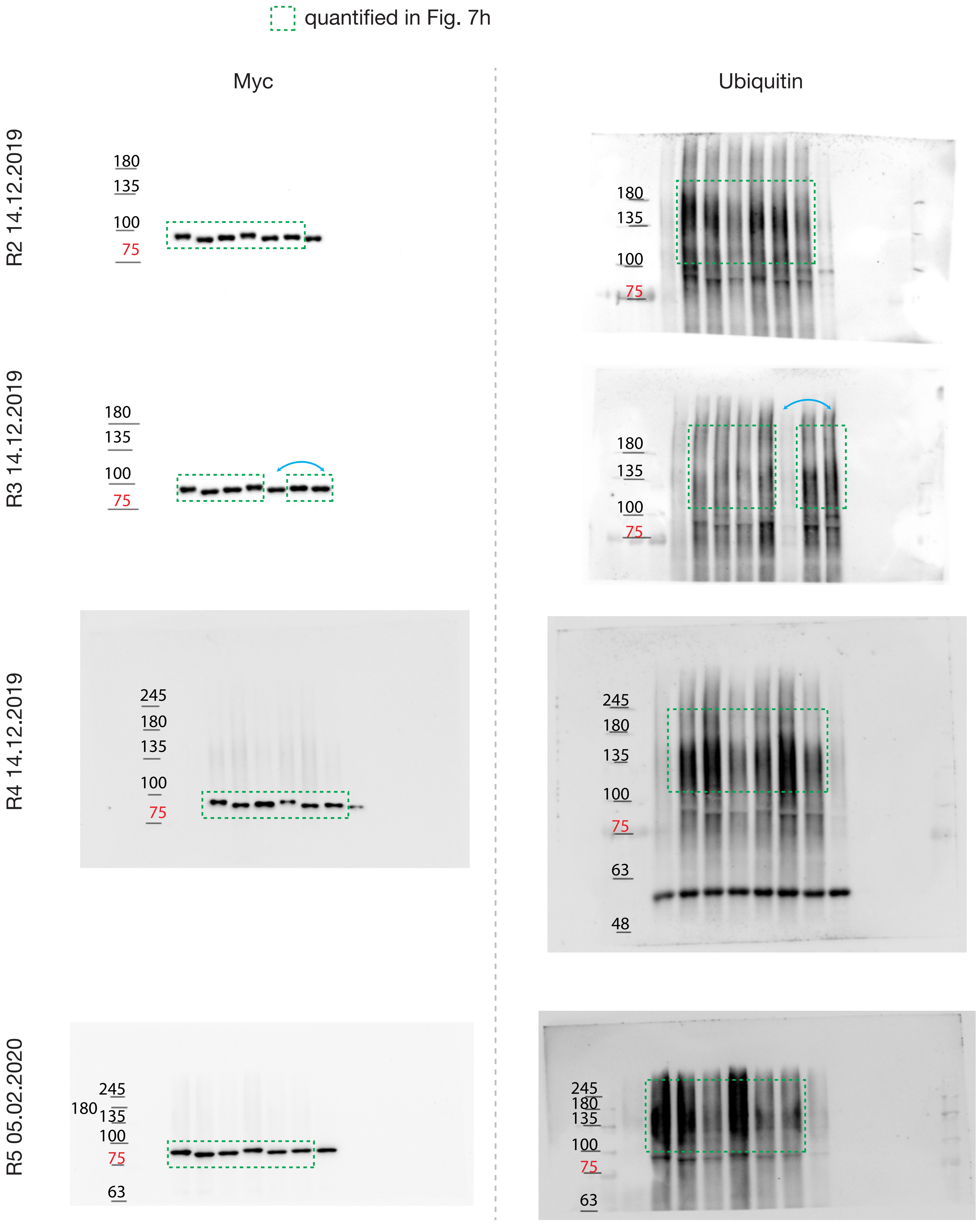
quantified in Fig. 7h
Myc
Ubiquitin
R2 14.12.2019
R3 14.12.2019
R4 14.12.2019
R5 05.02.2020
245
180
135
100
75
63
48

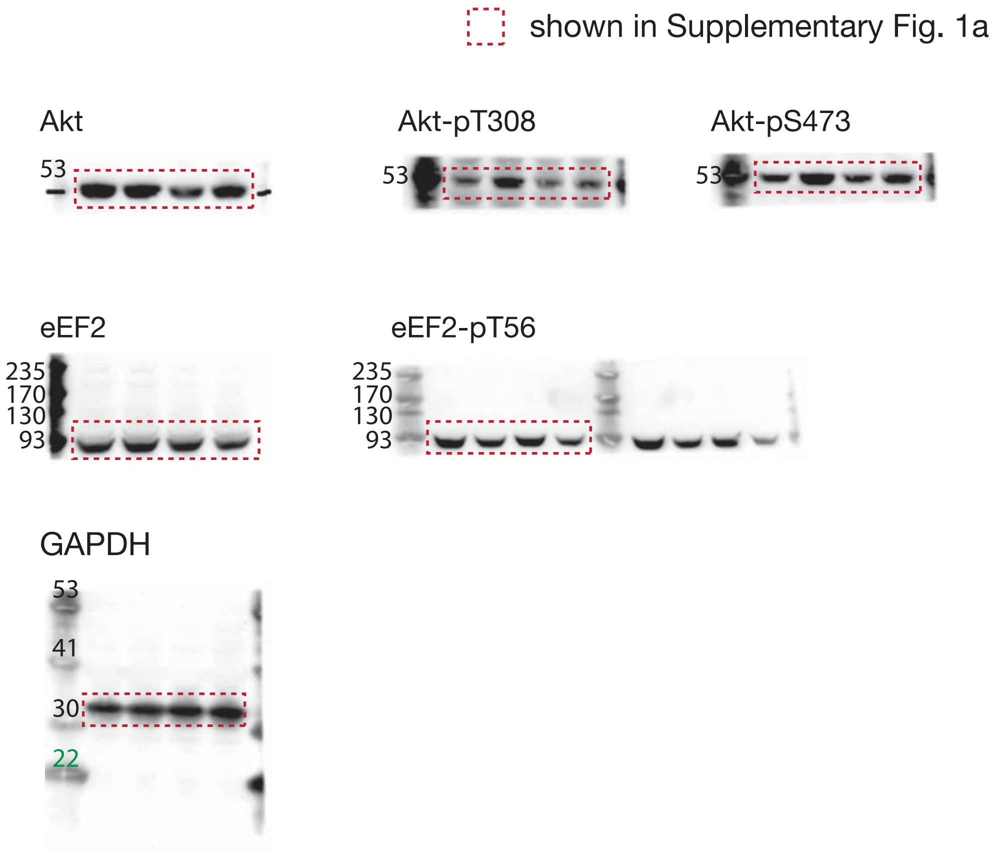
shown in Supplementary Fig. 1a
Akt
53
Akt-pT308
53
Akt-pS473
53
eEF2
235
170
130
93
eEF2-pT56
235
170
130
93
GAPDH
53
41
30
22

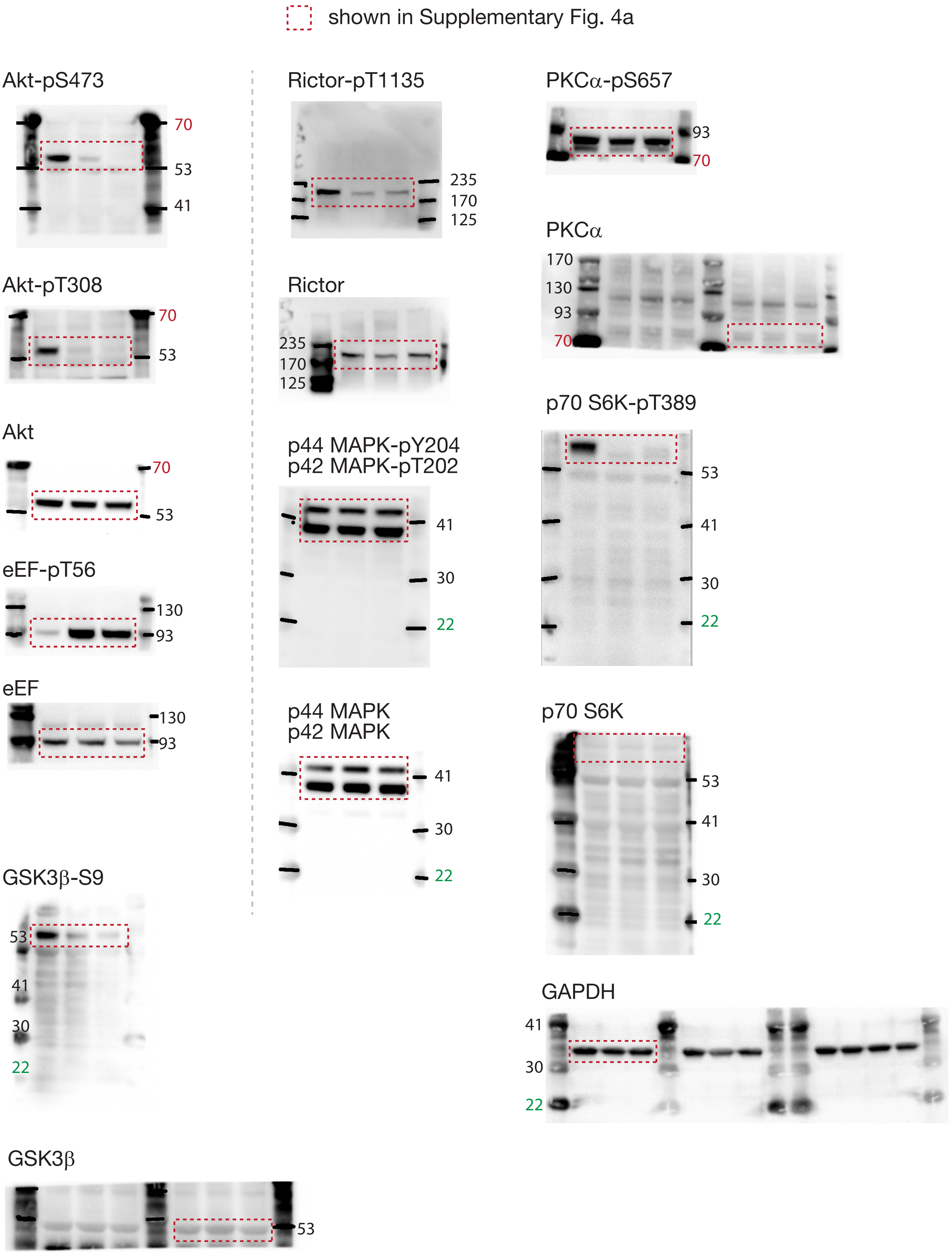

shown in Supplementary Fig. 4a
Akt-pS473
70
53
41
Akt-pT308
70
53
Akt
70
53
eEF-pT56
130
93
eEF
130
93
GSK3β-S9
53
41
30
22
GSK3β
53
Rictor-pT1135
235
170
125
Rictor
235
170
125
p44 MAPK-pY204
p42 MAPK-pT202
41
30
22
p44 MAPK
p42 MAPK
41
30
22
PKCα-pS657
93
70
PKCα
170
130
93
70
p70 S6K-pT389
53
41
30
22
p70 S6K
53
41
30
22
GAPDH
41
30
22

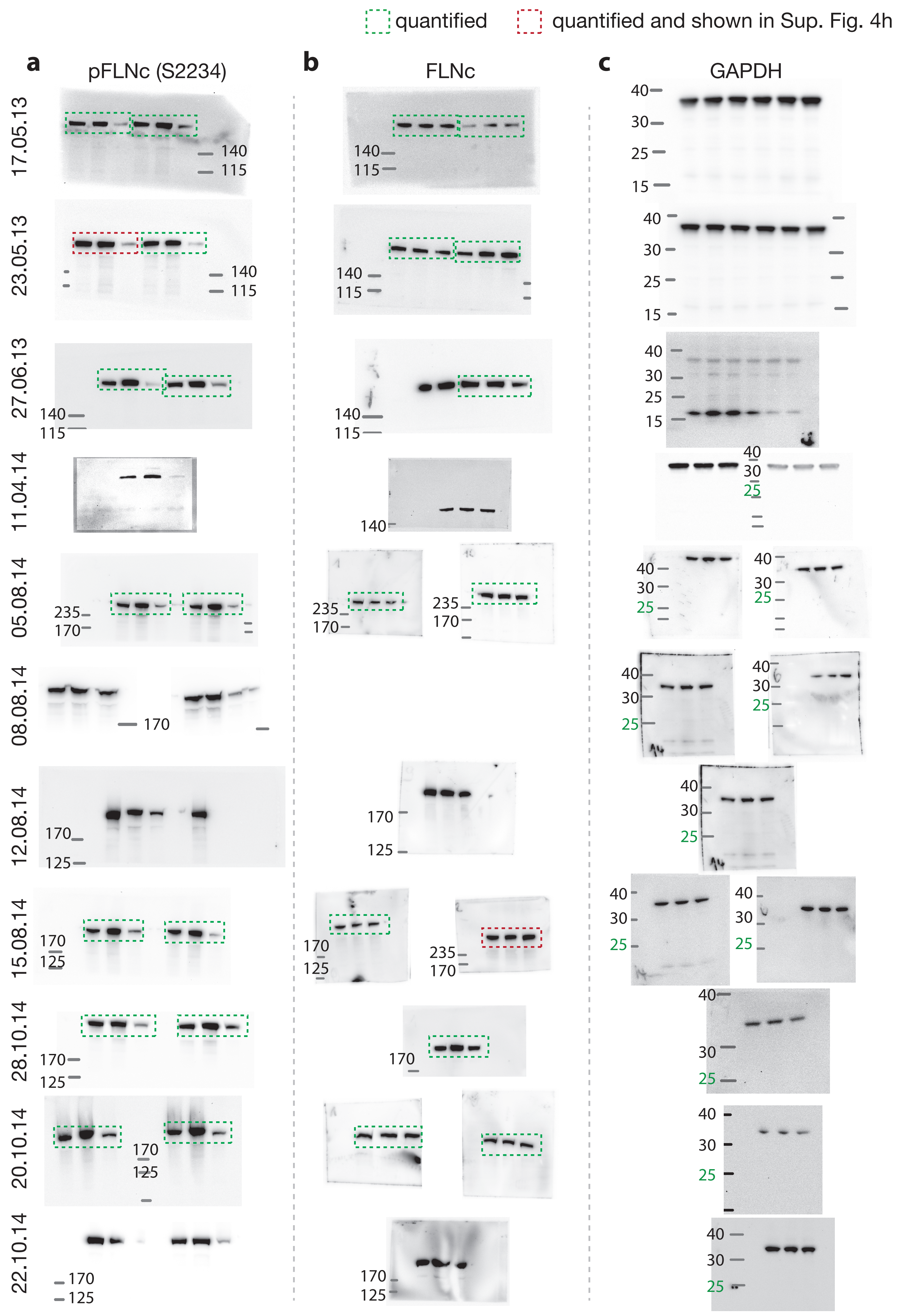

quantified
quantified and shown in Sup. Fig. 4h
a
pFLNc (S2234)
b
FLNc
c
GAPDH
17.05.13
23.05.13
27.06.13
11.04.14
05.08.14
08.08.14
12.08.14
15.08.14
28.10.14
20.10.14
22.10.14
140
115
235
170
125
40
30
25
15

shown in Supplementary Fig. 6b

FLNc-pS2233

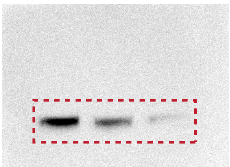

FLNc d16-20

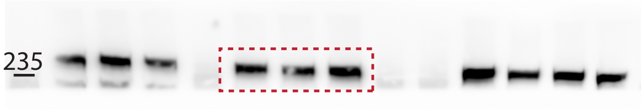

GAPDH

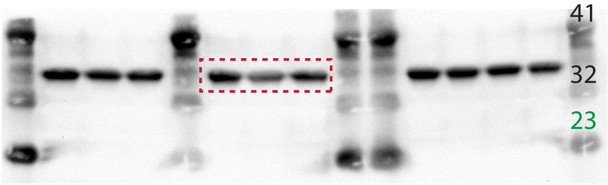

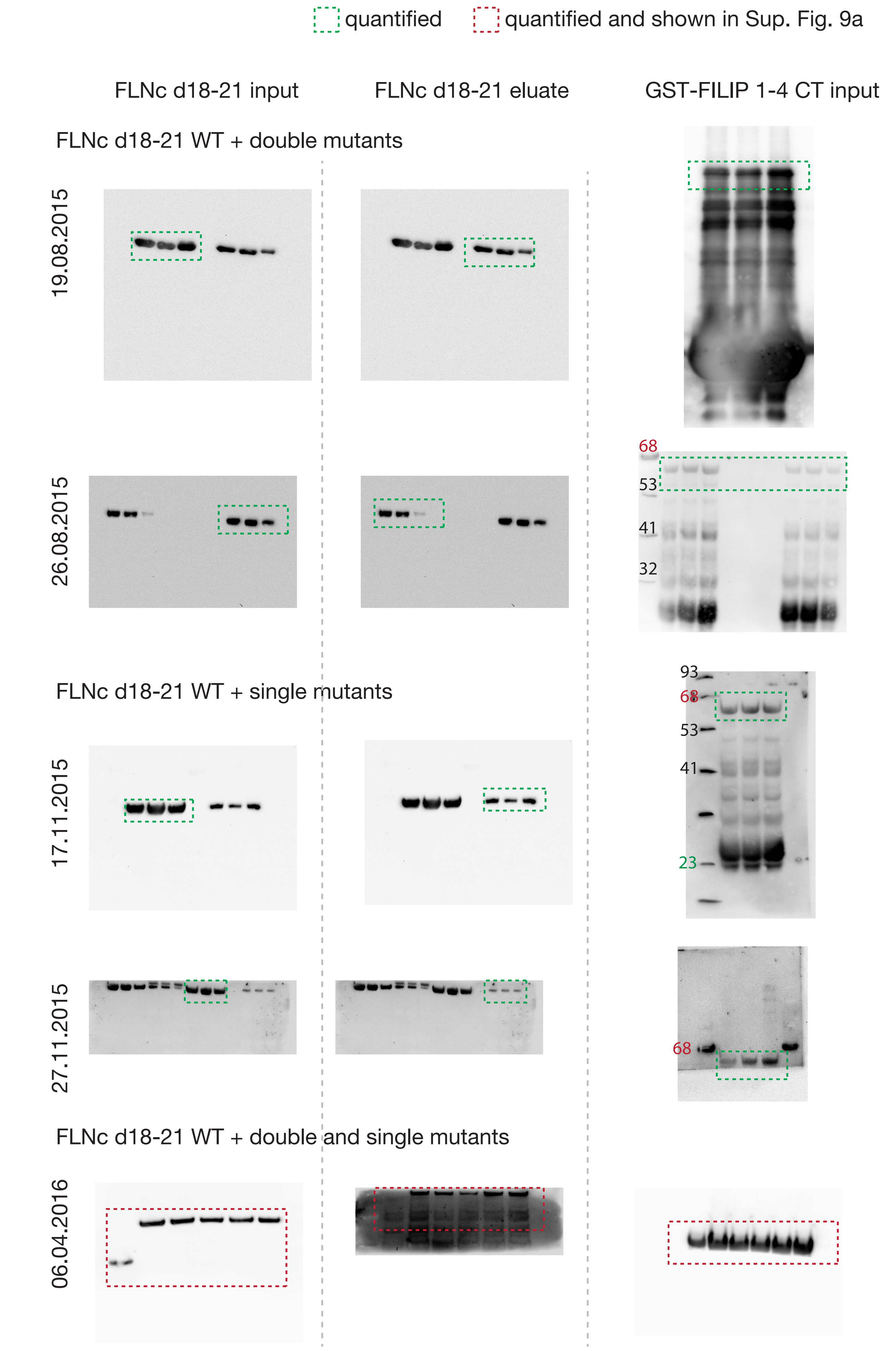

Supplementary Figure 2. Uncropped images of Western blots, shown in Fig. 1b, 4d, 5e, 6c, 6e, 7a, 7e, 7h/S13h, S1a, S4a, S4h, S6b and S9a.

Supplementary Figure 3

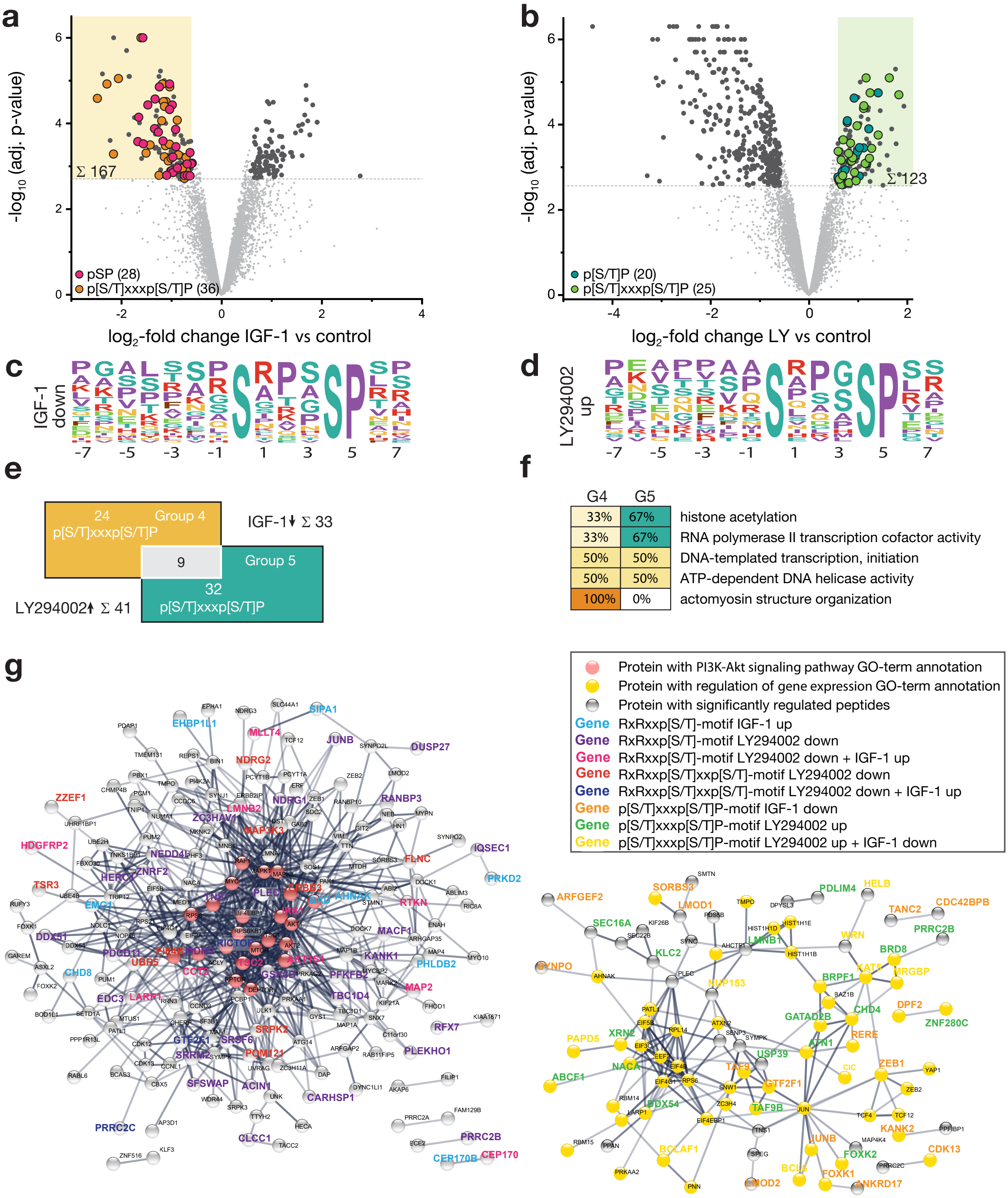

Supplementary Figure 3. Sequence motif analysis for proteins with phosphosites downregulated in response to PI3K/Akt/mTOR pathway activation or upregulated following inhibition. **a, b.** Volcano plots of phosphopeptides with localized phosphosites quantified in contracting C2 myotubes following IGF-1 (a) or LY294002 (b) treatment. Log_2-transformed mean SILAC ratios (control/treatment) were plotted against $-log_{10}$ adjusted p-values. Phosphopeptides with a minimum fold change of 1.5 and a FDR lower than 0.05 (n=3 independent experiments; moderated two-tailed student's t-test) are shown as larger dark gray circles. Among these, phosphopeptides are color-coded according to their phosphorylation sequence motif as indicated and regions of interest are highlighted in each plot. **c, d.** Motif-X analysis of regulated phosphopeptides. The proline-directed motif pSxxxSP was 30-fold and and 26-fold enriched following treatment with IGF-1 (c) and LY294002 (d), respectively. x, any amino acid. **e.** Overlap of unique phosphopeptides comprising the proline-directed motif p[S/T]xxxp[S/T]P in Group 4 (IGF-1 downregulated) and Group 5 (LY294002 upregulated). **f.** Combined GO enrichment analysis of phosphopeptides in group 4 and 5 (G4, G5) shown in (e). GO analysis was performed with the Cytoscape app ClueGO using Benjamini-Hochberg correction for p-value calculation. Only pathways with a p-value ≤ 0.05 were considered to be enriched. **g.** STRING network analysis of proteins with regulated phosphopeptides (fold-change ≥ 1.5; FDR < 0.05; n=3; moderated two-tailed student's t-test) following treatment of C2 myotubes with LY294002 or IGF-1. Proteins annotated as components of the PI3K/Akt pathway are highlighted by red bullets, proteins annotated with the GO term "gene expression" by yellow bullets. Gene names of proteins comprising a regulated phosphorylation site within a short or extended basophilic motif or the p[S/T]xxxp[S/T]P motif are color-coded as indicated.

Supplementary Figure 4

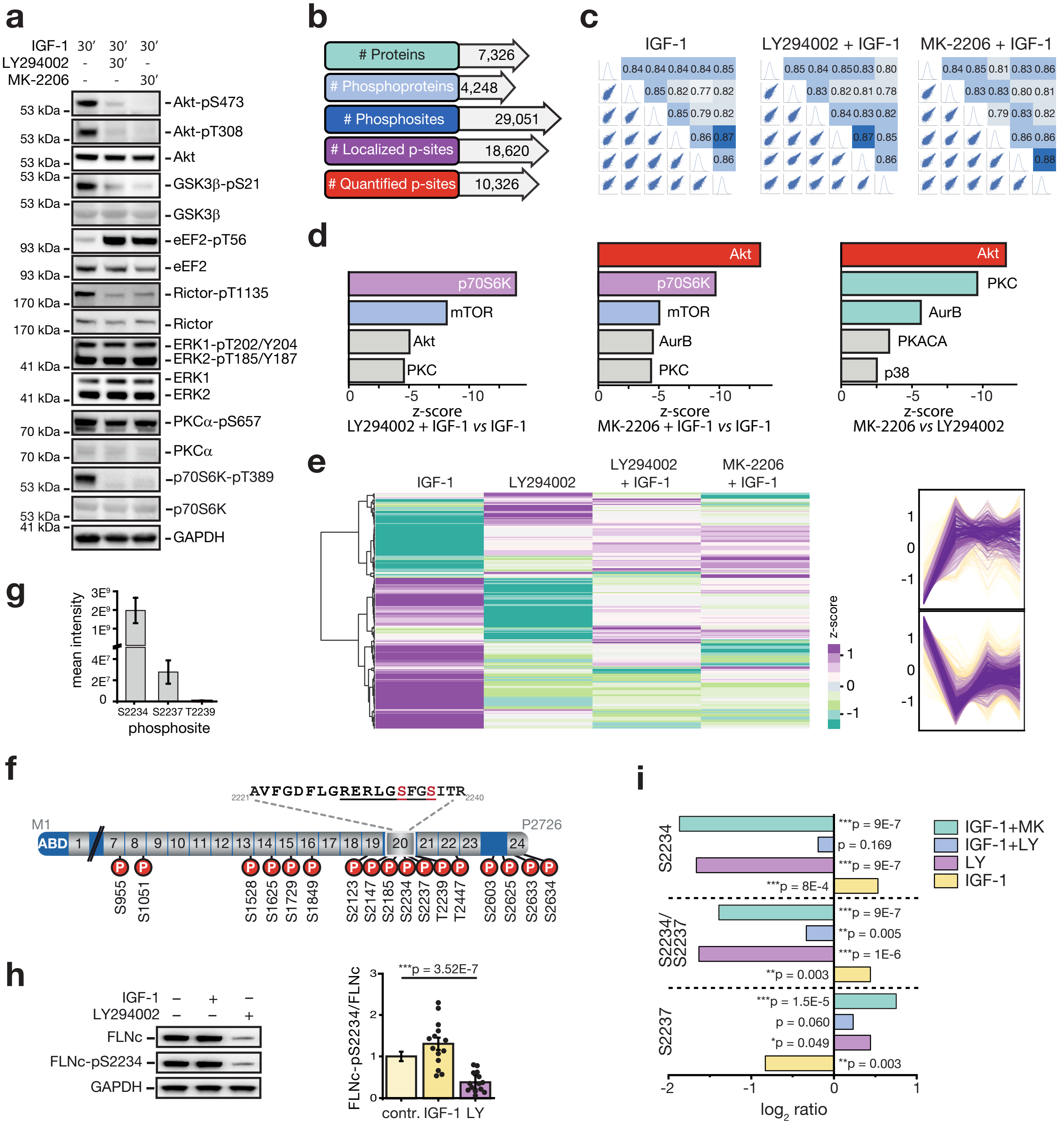

Supplementary Figure 4. Study of the PI3K/Akt/mTOR signaling landscape in contracting C2 myotubes using LY294002 (LY) and MK-2206 (MK). **a.** Contracting C2 myotubes, differentially labeled by SILAC amino acids, were treated for 30 min with IGF-1 in combination with LY or MK as indicated. Immunoblot analysis of PI3K/Akt/mTOR pathway activity using phospho-specific antibodies against established readouts. **b.** Overview of the number of identified proteins and phosphoproteins as well as the number of counted, localized (≥ 0.75) as well as localized and reproducibly quantified phosphosites in 3 out of 6 independent experiments obtained by quantitative MS-based phosphoproteome analysis and MaxQuant data analysis together with the IGF-1/LY data. **c.** Pearson correlation analysis of the phosphoproteomic data for the three different treatments (IGF-1, IGF-1+LY, IGF-1+MK, n=6). **d.** Z-score for the top down-regulated kinases based on kinase-substrate enrichment analysis (KSEA) for the different treatments as indicated. **e.** Hierarchical cluster analysis of all peptides that were significantly regulated in at least one of the four treatments (fold-change > ±1.5, adjusted p-value < 0.05 for IGF-1/LY dataset and < 0.01 for the LY/MK dataset) in the combined analysis (left panel). Two main cluster were obtained, the upper with 317 phosphopeptides, the lower with 560 phosphopeptides (right panel) **f.** Phosphosites of endogenous FLNc from mouse skeletal myotubes identified and quantified in this study. pS2234 and pS2237 are located in the extended basophilic motif RxRxxpSxxpS within the conserved Ig-like domain 20 of FLNc. **g.** Mean intensities of FLNc phosphopeptides with pS2234, pS2237 or pS2239 (n=3 independent experiments, SEM) **h.** Immunoblot analysis and quantification of signals detected for endogenous FLNc-pS2234 in comparison to total FLNc following treatment of mouse skeletal myotubes with IGF-1 or LY. Shown are normalized values from 14 replicates ± SEM. A paired two-tailed student's t-test was performed. **i.** FLNc phosphopeptides with the sequence 2232-LGSFGSITR-2240, either singly or doubly phosphorylated at S2234 and S2237, were regulated in abundance following activation (IGF-1) or inhibition (LY, IGF-1+MK, IGF-1+LY) of PI3K/Akt signaling. Shown are the $\log_2$ ratios and adjusted p-values calculated for each of the phosphosites.

Supplementary Figure 5

```
                                        10        20        30        40        50        60        70        80
                              ....|....|....|....|....|....|....|....|....|....|....|....|....|....|....|....|..
Homo sapiens                  GNWFQMVSAQERLTRTFTRSSHTYTRTERTEISKTRGGETKREVRVEESTQVGGDPFPAVFGDFLGRERLGSFGSITRQQEG
Mus musculus                  GNWFQMVSAQERLTRTFTRSSHTYTRTERTEISKTRGGETKREVRVEESTQVGGDPFPAVFGDFLGRERLGSFGSITRQQEG
Acinonyx jubatus              GNWFQMVSAQERLTRTFTRSSHTYTRTERTEISKTRGGETKREVRVEESTQVGGDPFPAVFGDFLGRERLGSFGSITRQQEG
Gulo gulo                     GNWFQMVSAQERLTRTFTRSSHTYTRTERTEISKTRGGETKREVRVEESTQVGGDPFPAVFGDFLGRERLGSFGSITRQQEG
Bison bison bison             GNWFQMVSAQERLTRTFTRSSHTYTRTERTEISKTRGGETKREVRVEESTQVGGDPFPAVFGDFLGRERLGSFGSITRQQEG
Castor canadensis             GNWFQMVSAQERLTRTFTRSSHTYTRTERTEISKTRGGETKREVRVEESTQVGGDPFPAVFGDFLGRERLGSFGSITRQQEG
Lynx canadensis               GNWFQMVSAQERLTRTFTRSSHTYTRTERTEISKTRGGETKREVRVEESTQVGGDPFPAVFGDFLGRERLGSFGSITRQQEG
Heterocephalus glaber         GNWFQMVSAQERLTRTFTRSSHTYTRTERTEISKTRGGETKREVRVEESTQVGGDPFPAVFGDFLGRERLGSFGSITRQQEG
Myotis lucifugus              GNWFQMVSAQERLTRTFTRSSHTYTRTERTEISKTRGGETKREVRVEESTQVGGDPFPAVFGDFLGRERLGSFGSITRQQEG
Elephantulus edwardii         GNWFQMVSAQERLTRTFTRSSHTYTRTERTEISKTRGGETKREVRVEESTQVGGDPFPAVFGDFLGRERLGSFGSITRQQEG
Peromyscus maniculatus bairdi GNWFQMVSAQERLTRTFTRSSHTYTRTERTEISKTRGGETKREVRVEESTQVGGDPFPAVFGDFLGRERLGSFGSITRQQEG
Physeter catodon              GNWFQMVSAQERLTRTFTRSSHTYTRTERTEISKTRGGETKREVRVEESTQVGGDPFPAVFGDFLGRERLGSFGSITRQQEG
Equus asinus                  GNWFQMVSAQERLTRTFTRSSHTYTRTERTEISKTRGGETKREVRVEESTQVGGDPFPAVFGDFLGRERLGSFGSITRQQEG
Fukomys damarensis            GNWFQMVSAQERLTRTFTRSSHTYTRTERTEISKTRGGETKREVRVEESTQVGGDPFPAVFGDFLGRERLGSFGSITRQQEG
Erinaceus europaeus           GNWFQMVSAQERLTRTFTRSSHTYTRTERTEISKTRGGETKREVRVEESTQVGGDPFPAVFGDFLGRERLGSFGSITRQQEG
Eptesicus fuscus              GNWFQMVSAQERLTRTFTRSSHTYTRTERTEISKTRGGETKREVRVEESTQVGGDPFPAVFGDFLGRERLGSFGSITRQQEG
Phyllostomus discolor         GNWFQMVSAQERLTRTFTRSSHTYTRTERTEISKTRGGETKREVRVEESTQVGGDPFPAVFGDFLGRERLGSFGSITRQQEG
Myotis davidii                GNWFQMVSAQERLTRTFTRSSHTYTRTERTEISKTRGGETKREVRVEESTQVGGDPFPAVFGDFLGRERLGSFGSITRQQEG
Octodon degus                 GNWFQMVSAQERLTRTFTRSSHTYTRTERTEISKTRGGETKREVRVEESTQVGGDPFPAVFGDFLGRERLGSFGSITRQQEG
Chinchilla lanigera           GNWFQMVSAQERLTRTFTRSSHTYTRTERTEISKTRGGETKREVRVEESTQVGGDPFPAVFGDFLGRERLGSFGSITRQQEG
Dipodomys ordii               GNWFQMVSAQERLTRTFTRSSHTYTRTERTEISKTRGGETKREVRVEESTQVGGDPFPAVFGDFLGRERLGSFGSITRQQEG
Nannospalax galili            GNWFQMVSAQERLTRTFTRSSHTYTRTERTEISKTRGGETKREVRVEESTQVGGDPFPAVFGDFLGRERLGSFGSITRQQEG
Acinonyx jubatus              GNWFQMVSAQERLTRTFTRSSHTYTRTERTEISKTRGGETKREVRVEESTQVGGDPFPAVFGDFLGRERLGSFGSITRQQEG
Sus scrofa                    GNWFQMVSAQERLTRTFTRSSHTYTRTERTEISKTRGGETKREVRVEESTQVGGDPFPAVFGDFLGRERLGSFGSITRQQEG
Galeopterus variegatus        GNWFQMVSAQERLTRTFTRSSHTYTRTERTEISKTRGGETKREVRVEESTQVGGDPFPAVFGDFLGRERLGSFGSITRQQEG
Eptesicus fuscus              GNWFQMVSAQERLTRTFTRSSHTYTRTERTEISKTRGGETKREVRVEESTQVGGDPFPAVFGDFLGRERLGSFGSITRQQEG
Canis lupus familiaris        GNWFQMVSAQERLTRTFTRSSHTYTRTERTEISKTRGGETKREVRVEESTQVGGDPFPAVFGDFLGRERLGSFGSITRQQEG
Mustela putorius furo         GNWFQMVSAQERLTRTFTRSSHTYTRTERTEISKTRGGETKREVRVEESTQVGGDPFPAVFGDFLGRERLGSFGSITRQQEG
Panthera pardus               GNWFQMVSAQERLTRTFTRSSHTYTRTERTEISKTRGGETKREVRVEESTQVGGDPFPAVFGDFLGRERLGSFGSITRQQEG
Felis catus                   GNWFQMVSAQERLTRTFTRSSHTYTRTERTEISKTRGGETKREVRVEESTQVGGDPFPAVFGDFLGRERLGSFGSITRQQEG
Canis lupus dingo             GNWFQMVSAQERLTRTFTRSSHTYTRTERTEISKTRGGETKREVRVEESTQVGGDPFPAVFGDFLGRERLGSFGSITRQQEG
Mastomys coucha               GNWFQMVSAQERLTRTFTRSSHTYTRTERTEISKTRGGETKREVRVEESTQVGGDPFPAVFGDFLGRERLGSFGSITRQQEG
Piliocolobus tephrosceles     GNWFQMVSAQERLTRTFTRSSHTYTRTERTEISKTRGGETKREVRVEESTQVGGDPFPAVFGDFLGRERLGSFGSITRQQEG
Equus caballus                GNWFQMVSAQERLTRTFTRSSHTYTRTERTEISKTRGGETKREVRVEESTQVGGDPFPAVFGDFLGRERLGSFGSITRQQEG
Peromyscus leucopus           GNWFQMVSAQERLTRTFTRSSHTYTRTERTEISKTRGGETKREVRVEESTQVGGDPFPAVFGDFLGRERLGSFGSITRQQEG
Oryctolagus cuniculus         GNWFQMVSAQERLTRTFTRSSHTYTRTERTEISKTRGGETKREVRVEESTQVGGDPFPAVFGDFLGRERLGSFGSITRQQEG
Colobus angolensis palliatus  GNWFQMVSAQERLTRTFTRSSHTYTRTERTEISKTRGGETKREVRVEESTQVGGDPFPAVFGDFLGRERLGSFGSITRQQEG
Cricetulus griseus            GNWFQMVSAQERLTRTFTRSSHTYTRTERTEISKTRGGETKREVRVEESTQVGGDPFPAVFGDFLGRERLGSFGSITRQQEG
Tupaia chinensis              GNWFQMVSAQERLTRTFTRSSHTYTRTERTEISKTRGGETKREVRVEESTQVGGDPFPAVFGDFLGRERLGSFGSITRQQEG
Delphinapterus leucas         GNWFQMVSAQERLTRTFTRSSHTYTRTERTEISKTRGGETKREVRVEESTQVGGDPFPAVFGDFLGRERLGSFGSITRQQEG
Dasypus novemcinctus          GNWFQMVSAQERLTRTFTRSSHTYTRTERTEISKTRGGETKREVRVEESTQVGGDPFPAVFGDFLGRERLGSFGSITRQQEG
Cricetulus griseus            GNWFQMVSAQERLTRTFTRSSHTYTRTERTEISKTRGGETKREVRVEESTQVGGDPFPAVFGDFLGRERLGSFGSITRQQEG
Physeter catodon              GNWFQMVSAQERLTRTFTRSSHTYTRTERTEISKTRGGETKREVRVEESTQVGGDPFPAVFGDFLGRERLGSFGSITRQQEG
Myotis davidii                GNWFQMVSAQERLTRTFTRSSHTYTRTERTEISKTRGGETKREVRVEESTQVGGDPFPAVFGDFLGRERLGSFGSITRQQEG
Fukomys damarensis            GNWFQMVSAQERLTRTFTRSSHTYTRTERTEISKTRGGETKREVRVEESTQVGGDPFPAVFGDFLGRERLGSFGSITRQQEG
Marmota flaviventris          GNWFQMVSAQERLTRTFTRSSHTYTRTERTEISKTRGGETKREVRVEESTQVGGDPFPAVFGDFLGRERLGSFGSITRQQEG
Lipotes vexillifer            GNWFQMVSAQERLTRTFTRSSHTYTRTERTEISKTRGGETKREVRVEESTQVGGDPFPAVFGDFLGRERLGSFGSITRQQEG
Theropithecus gelada          GNWFQMVSAQERLTRTFTRSSHTYTRTERTEISKTRGGETKREVRVEESTQVGGDPFPAVFGDFLGRERLGSFGSITRQQEG
Cercocebus atys               GNWFQMVSAQERLTRTFTRSSHTYTRTERTEISKTRGGETKREVRVEESTQVGGDPFPAVFGDFLGRERLGSFGSITRQQEG
Dasypus novemcinctus          GNWFQMVSAQERLTRTFTRSSHTYTRTERTEISKTRGGETKREVRVEESTQVGGDPFPAVFGDFLGRERLGSFGSITRQQEG
Macaca mulatta                GNWFQMVSAQERLTRTFTRSSHTYTRTERTEISKTRGGETKREVRVEESTQVGGDPFPAVFGDFLGRERLGSFGSITRQQEG
Panthera tigris altaica       GNWFQMVSAQERLTRTFTRSSHTYTRTERTEISKTRGGETKREVRVEESTQVGGDPFPAVFGDFLGRERLGSFGSITRQQEG
Delphinapterus leucas         GNWFQMVSAQERLTRTFTRSSHTYTRTERTEISKTRGGETKREVRVEESTQVGGDPFPAVFGDFLGRERLGSFGSITRQQEG
Fukomys damarensis            GNWFQMVSAQERLTRTFTRSSHTYTRTERTEISKTRGGETKREVRVEESTQVGGDPFPAVFGDFLGRERLGSFGSITRQQEG
Cavia porcellus               GNWFQMVSAQERLTRTFTRSSHTYTRTERTEISKTRGGETKREVRVEESTQVGGDPFPAVFGDFLGRERLGSFGSITRQQEG
Chlorocebus sabaeus           GNWFQMVSAQERLTRTFTRSSHTYTRTERTEISKTRGGETKREVRVEESTQVGGDPFPAVFGDFLGRERLGSFGSITRQQEG
Marmota flaviventris          GNWFQMVSAQERLTRTFTRSSHTYTRTERTEISKTRGGETKREVRVEESTQVGGDPFPAVFGDFLGRERLGSFGSITRQQEG
Erinaceus europaeus           GNWFQMVSAQERLTRTFTRSSHTYTRTERTEISKTRGGETKREVRVEESTQVGGDPFPAVFGDFLGRERLGSFGSITRQQEG
Equus asinus                  GNWFQMVSAQERLTRTFTRSSHTYTRTERTEISKTRGGETKREVRVEESTQVGGDPFPAVFGDFLGRERLGSFGSITRQQEG
Bos indicus                   GNWFQMVSAQERLTRTFTRSSHTYTRTERTEISKTRGGETKREVRVEESTQVGGDPFPAVFGDFLGRERLGSFGSITRQQEG
Bos indicus x Bos taurus      GNWFQMVSAQERLTRTFTRSSHTYTRTERTEISKTRGGETKREVRVEESTQVGGDPFPAVFGDFLGRERLGSFGSITRQQEG
Ornithorhynchus anatinus      GNWFQMVSAQERLTRTFTRSSHTYTRTERTEISKTRGGETKREVRVEESTQVGGDPFPAVFGDFLGRERLGSFGSITRQQEG
Jaculus jaculus               GNWFQMVSAQERLTRTFTRSSHTYTRTERTEISKTRGGETKREVRVEESTQVGGDPFPAVFGDFLGRERLGSFGSITRQQEG
Loxodonta africana            GNWFQMVSAQERLTRTFTRSSHTYTRTERTEISKTRGGETKREVRVEESTQVGGDPFPAVFGDFLGRERLGSFGSITRQQEG
Heterocephalus glaber         GNWFQMVSAQERLTRTFTRSSHTYTRTERTEISKTRGGETKREVRVEESTQVGGDPFPAVFGDFLGRERLGSFGSITRQQEG
Hipposideros armiger          GNWFQMVSAQERLTRTFTRSSHTYTRTERTEISKTRGGETKREVRVEESTQVGGDPFPAVFGDFLGRERLGSFGSITRQQEG
Myotis brandtii               GNWFQMVSAQERLTRTFTRSSHTYTRTERTEISKTRGGETKREVRVEESTQVGGDPFPAVFGDFLGRERLGSFGSITRQQEG
Vulpes vulpes                 GNWFQMVSAQERLTRTFTRSSHTYTRTERTEISKTRGGETKREVRVEESTQVGGDPFPAVFGDFLGRERLGSFGSITRQQEG
Ornithorhynchus anatinus      GNWFQMVSAQERLTRTFTRSSHTYTRTERTEISKTRGGETKREVRVEESTQVGGDPFPAVFGDFLGRERLGSFGSITRQQEG
Saimiri boliviensis boliviens GNWFQMVSAQERLTRTFTRSSHTYTRTERTEISKTRGGETKREVRVEESTQVGGDPFPAVFGDFLGRERLGSFGSITRQQEG
Desmodus rotundus             GNWFQMVSAQERLTRTFTRSSHTYTRTERTEISKTRGGETKREVRVEESTQVGGDPFPAVFGDFLGRERLGSFGSITRQQEG
Orcinus orca                  GNWFQMVSAQERLTRTFTRSSHTYTRTERTEISKTRGGETKREVRVEESTQVGGDPFPAVFGDFLGRERLGSFGSITRQQEG
Enhydra lutris kenyoni        GNWFQMVSAQERLTRTFTRSSHTYTRTERTEISKTRGGETKREVRVEESTQVGGDPFPAVFGDFLGRERLGSFGSITRQQEG
Cebus capucinus imitator      GNWFQMVSAQERLTRTFTRSSHTYTRTERTEISKTRGGETKREVRVEESTQVGGDPFPAVFGDFLGRERLGSFGSITRQQEG
Colobus angolensis palliatus  GNWFQMVSAQERLTRTFTRSSHTYTRTERTEISKTRGGETKREVRVEESTQVGGDPFPAVFGDFLGRERLGSFGSITRQQEG
Nomascus leucogenys           GNWFQMVSAQERLTRTFTRSSHTYTRTERTEISKTRGGETKREVRVEESTQVGGDPFPAVFGDFLGRERLGSFGSITRQQEG
Microcebus murinus            GNWFQMVSAQERLTRTFTRSSHTYTRTERTEISKTRGGETKREVRVEESTQVGGDPFPAVFGDFLGRERLGSFGSITRQQEG
Macaca fascicularis           GNWFQMVSAQERLTRTFTRSSHTYTRTERTEISKTRGGETKREVRVEESTQVGGDPFPAVFGDFLGRERLGSFGSITRQQEG
Vulpes vulpes                 GNWFQMVSAQERLTRTFTRSSHTYTRTERTEISKTRGGETKREVRVEESTQVGGDPFPAVFGDFLGRERLGSFGSITRQQEG
Equus asinus                  GNWFQMVSAQERLTRTFTRSSHTYTRTERTEISKTRGGETKREVRVEESTQVGGDPFPAVFGDFLGRERLGSFGSITRQQEG
Cavia porcellus               GNWFQMVSAQERLTRTFTRSSHTYTRTERTEISKTRGGETKREVRVEESTQVGGDPFPAVFGDFLGRERLGSFGSITRQQEG
Pan paniscus                  GNWFQMVSAQERLTRTFTRSSHTYTRTERTEISKTRGGETKREVRVEESTQVGGDPFPAVFGDFLGRERLGSFGSITRQQEG
Piliocolobus tephrosceles     GNWFQMVSAQERLTRTFTRSSHTYTRTERTEISKTRGGETKREVRVEESTQVGGDPFPAVFGDFLGRERLGSFGSITRQQEG
Pan troglodytes               GNWFQMVSAQERLTRTFTRSSHTYTRTERTEISKTRGGETKREVRVEESTQVGGDPFPAVFGDFLGRERLGSFGSITRQQEG
Papio anubis                  GNWFQMVSAQERLTRTFTRSSHTYTRTERTEISKTRGGETKREVRVEESTQVGGDPFPAVFGDFLGRERLGSFGSITRQQEG
Gorilla gorilla gorilla       GNWFQMVSAQERLTRTFTRSSHTYTRTERTEISKTRGGETKREVRVEESTQVGGDPFPAVFGDFLGRERLGSFGSITRQQEG
Mus caroli                    GNWFQMVSAQERLTRTFTRSSHTYTRTERTEISKTRGGETKREVRVEESTQVGGDPFPAVFGDFLGRERLGSFGSITRQQEG
Mus pahari                    GNWFQMVSAQERLTRTFTRSSHTYTRTERTEISKTRGGETKREVRVEESTQVGGDPFPAVFGDFLGRERLGSFGSITRQQEG mammals
CONSENSUS                     ------------------------------------------------------------------RXRXXSXXS-------
```

Supplementary Figure 5. Sequence alignment across mammalian species of the FLNc specific insertion in domain 20, comprising 82 amino acids.

Supplementary Figure 6

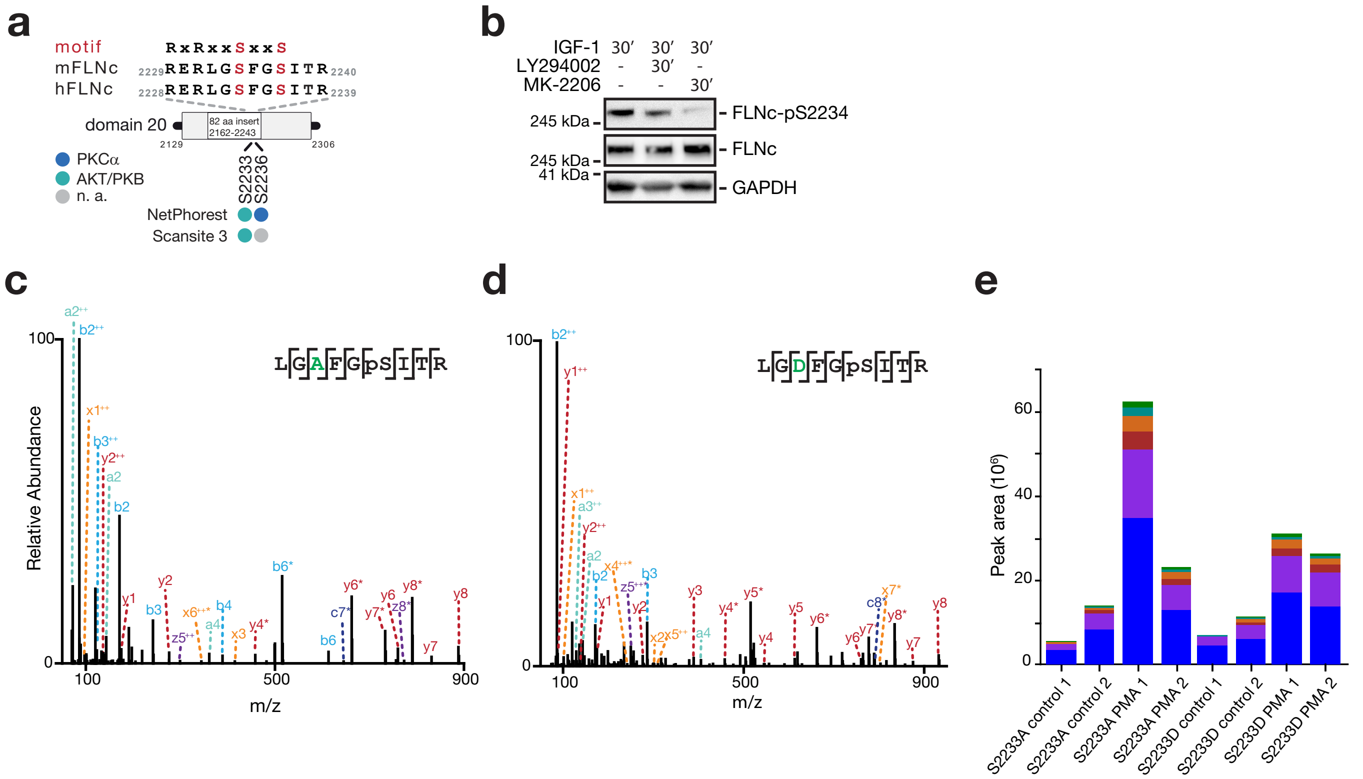

Supplementary Figure 6. Analysis of phosphorylation status of endogenous mouse (m) FLNc as well as human (h) FLNc d18-21 S2233D and S2233A mutants. **a.** Kinase prediction for pS2233 and pS2236 of mFLNc and hFLNc using the algorithms NetPhorest and Scansite 3. Both phosphosites are part of the extended basophilic motif located in the unique 82-amino acid insert in domain 20, which is highly conserved in mammals. **b.** Immunoblot analysis of mFLNc phosphorylation at S2234 in C2 myotubes in dependency of MK-2206 and LY294002 inhibition in combination IGF-1 as indicated. **c, d.** C2 cells expressing hFLNc d18-21 in which S2233 was mutated to alanine (A) or aspartate (D) were treated with PMA for activation of PKC followed by phosphopeptide analysis in a targeted MS approach using higher collisional dissociation (HCD) as outlined in Figure 4c. HCD spectrum of the phosphopeptide (a) LGAFGpSITR from the FLNc d18-21 S2233A mutant and (b) LGDFGpSITR from the FLNc d18-21 S2233D mutant. The site-exchanged amino acids (S to A, S to D) are highlighted in green. **e.** Precursor and transition areas determined by Skyline analysis of the targeted MS data obtained for the phosphopeptides shown in (c) and (d).

Supplementary Figure 7

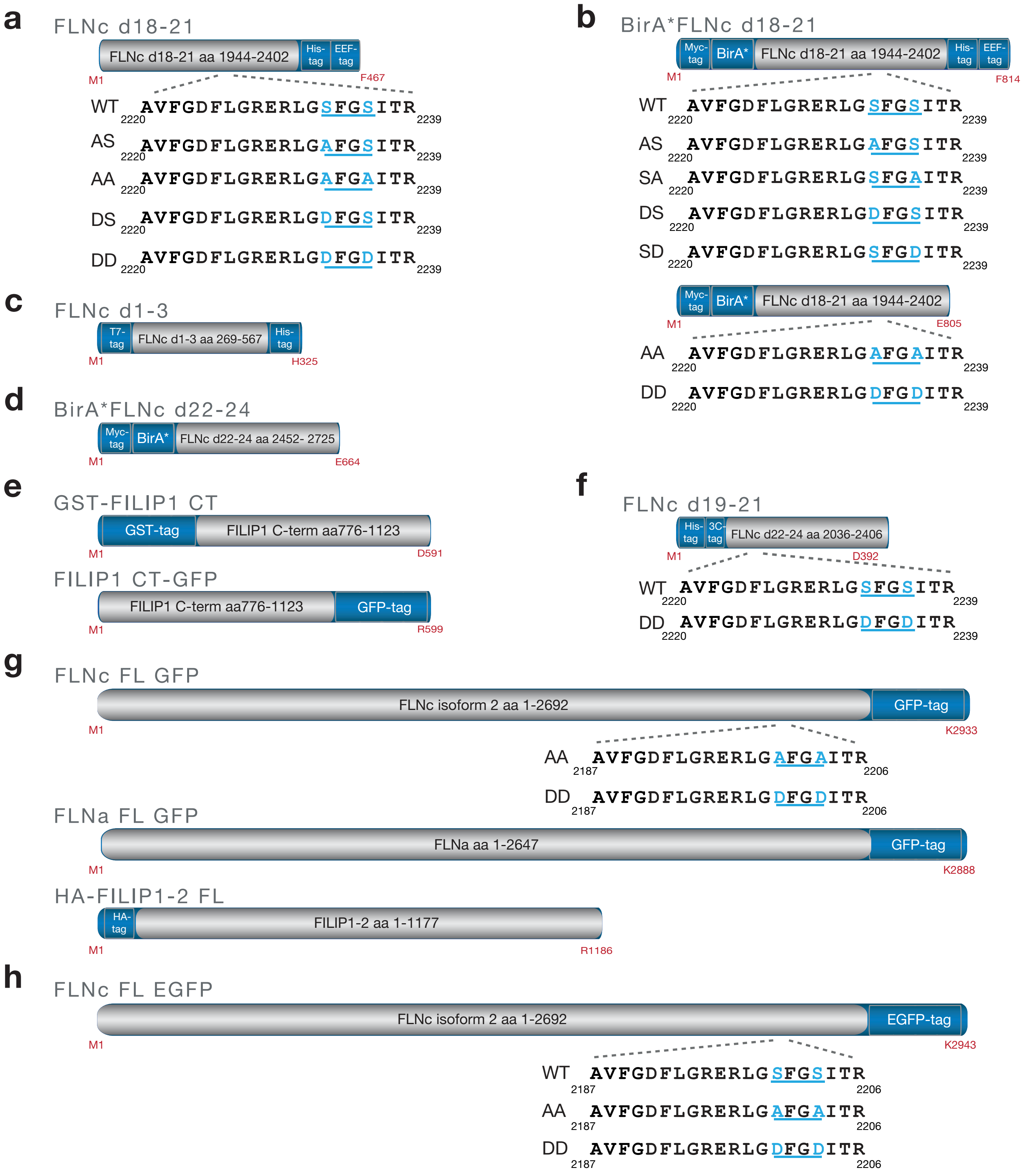

Supplementary Figure 7. FLNc phosphosite mutants and fusion proteins used in this work. **a.** Schematic illustration of human FLNc d18-21 constructs used for bacterial transformations comprising a 6xHis tag and an EEF tag fused to the carboxy-terminus. Phosphosite mutants were generated from the respective wild-type construct by exchange of one or two serines (S) to alanine (A) or aspartate (D) as indicated. WT, wild-type; d, domain **b.** Schematic illustration of human FLNc d18-21 constructs used for transfections of mammalian cells comprising a Myc-tag and a BirA* sequence fused to the amino-terminus. Additionally, wild-type and single site mutants contain a 6xHis tag and an EEF tag fused to the carboxy-terminus. Phosphosite mutants were generated as described in (a). BirA*, promiscuous biotin ligase. **c.** Schematic illustration of human FLNc d1-3 construct used for bacterial transformations comprising a T7 tag and a 6xHis tag fused to the amino- and carboxy-terminus, respectively. **d.** Schematic illustration of human FLNc d22-24 construct used for transfections of mammalian cells comprising a Myc-tag and a BirA* sequence fused to the amino-terminus. **e.** Schematic illustration of FILIP1 carboxy-terminal constructs. For transformation of bacterial cells, the construct was fused to GST at its amino-terminus. For transfection of mammalian cells, the construct was fused to GFP at its carboxy-terminus. CT, carboxy-terminus. **f.** Schematic illustration of the phosphomimicking human FLNc d19-21 construct used for fluorescence correlation spectroscopy analysis comprising a 6xHis tag fused to the amino-terminus. The phosphomimicking mutant was generated from the respective wild-type construct by exchange of two serine residues (SS) to aspartate (DD) as indicated. WT, wild-type. **g.** Schematic illustration of constructs used for co-expression of FLNa, FLNc and FILIP1. FLNa and FLNc full-length constructs were fused to GFP at its carboxy-terminus. FLNc double phosphosite mutants were generated from the respective WT construct by exchange of two serine residues (SS) to alanine (AA) or aspartate (DD) as indicated. FILIP1-2 was fused to an HA-tag at its amino-terminus. FL, full-length. **h.** Schematic illustration of FLNc constructs used for fluorescence recovery after photobleach fused to a carboxy-terminal EGFP. Phosphomimicking mutants were generated from the respective wild-type construct by exchange of two serine residues (SS) to aspartate (DD) or alanine (AA) as indicated. FL, full length. WT, wild-type.

Supplementary Figure 8

a

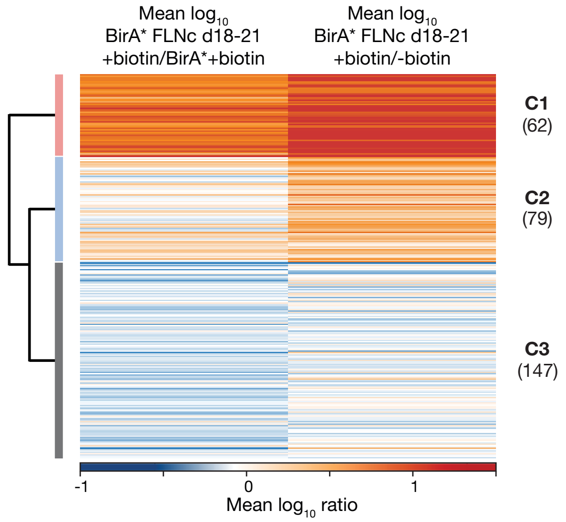

b

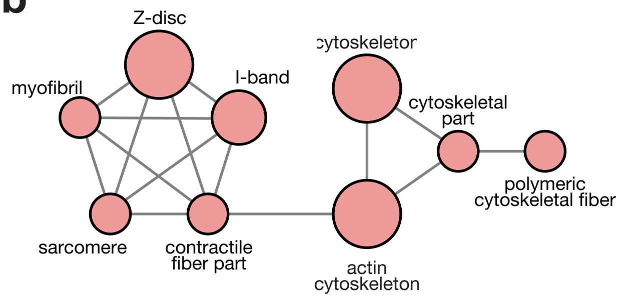

c

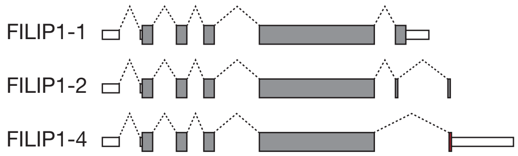

d

```
Q7Z7B0     FILIP1-1  1121     GASKVTSTITITPVTTSSARGTQSV---SGQDGSSQRPTPTRIPMSKGMKAGKPVVAAPGAGNLTKFEPRAETQSMKIELKKSAASSTTSLGGGKG    1213
Q7Z7B0-2   FILIP1-2  1121     GASKVTSTITITPVTTSSARGTQSV---SGQDGSSQRPTPTRIPMSK---ESIIIHQLRMNSR                                     1177
Q7Z7B0-3   FILIP1-3  873      GASKVTSTITITPVTTSSARGTQSV---SGQDGSSQRPTPTRIPMSKGMKAGKPVVAAPGAGNLTKFEPRAETQSMKIELKKSAASSTTSLGGGKG     965
Novel      FILIP1-4  1121     GASKVTSTITITPVTTSSARGTQSV---KASSFTSYE                                                               1154
```

e

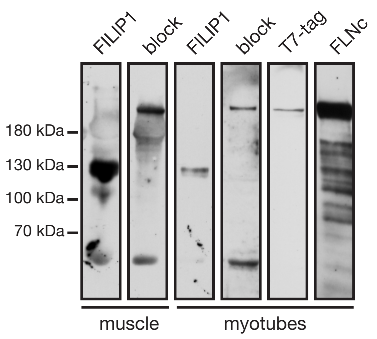

f

FLNc domains	lacZ	His3
d16-19	+	+++
d16-17	-	+
d17-19	+++	+++
d18-21	+++	+++
d18-19	-	-
d20-21	++	++

g

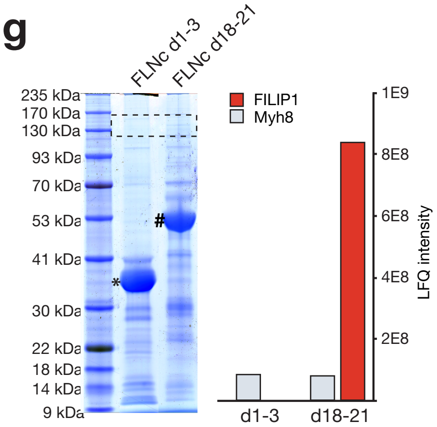

h

FLNC_MOUSE Isoform 2 of Filamin-C Q8VHX6-2
Protein sequence coverage: 78%

```
1    MMNNSNYSDA SGLGLVDEAD EMPSTEKDLA EDAPWKKIQQ NTFTRWCNEH LKCVGKRLTD LQRDLSDGLR LIALLEVLSQ KRMYRKFHPR PNFRQMKLEN
101  VSVALEFLER EHIKLVSIDS KAIVDGNLKL ILGLIWTLIL HYSISMPMWE DEDDEDARKQ TPKQRLLGWI QNKVPQLPIT NFNRDWQDGK ALGALVDNCA
201  PGLCPDWEAW DPNQPVQNAR EAMQQADDWL GVPQVIAPEE IVDPNVDEHS VMTYLSQFPK AKLKPGAPVR SKQLNPKKAI AYGPGIEPQG NTVLQPAHFT
301  VQTVDAGVGE VLVYIEDPEG HTEEAKVVPN NDKDRTYAVS YVPKVAGLHK VTVLFAGQNI ERSPFEVNVG MALGDANKVS ARGPGLEPVG NVANKPTYFD
401  IYTAGAGTGD VAVVIVDPQG RRDTVEVALE DKGDNTFRCT YRPVMEGPHT VHVAFAGAPI TRSPFPVHVA EACNPNACRA SGRGLQPKGV RVKEVADFKV
501  FTKGAGSGEL KVTVKGPKGT EEPVKVREAG DGVFECEYYP VVPGKYVVTI TWGGYAIPRS PFEVQVSPEA GAQKVRAWGP GLETGQVGKS ADFVVEAIGT
601  EVGTLGFSIE GPSQAKIECD DKGDGSCDVR YWPTEPGEYA VHVICDDEDI RDSPFIAHIQ PAPPDCFPDK VKAFGPGLEP TGCIVDRPAE FTIDARAAGK
701  GDLKLYAQDA DGCPIDIKVI PNGDGTFRCS YVPTKPIKHT IIVSWGGVNV PKSPFRVNVG EGSHPERVKV YGPGVEKTGL KANEPTYFTV DCSEAGQGDV
801  SIGIKCAPGV VGPVEADIDF DIIKNDNDTF TVKYTPPGAG HYTIMVLFAN QEIPASPFHI KVDPSHDASK VKAEGPGLSR TGVEVGKPTH FTVLTKGAGK
901  AKLDVHFAGA AKGEAVRDFE IIDNHDYSYT VKYTAVQQGN MAVTVTYGGD PVPKSPFVVN VAPPLDLSKV KVQGLNSKVA VGQEQAFSVN TRGAGGQGQL
1001 DVRMTSPSRR PIPCKLEPGG GAEAQAVRYM PPEEGPYKVD ITYDGHPVPG SPFAVEGVLP PDPSKVCAYG PGLKGGLVGT PAPFSIDTKG AGTGGLGLTV
1101 EGPCEAKIEC QDNGDGSCAV SYLPTEPGEY TINILFAEAH IPGSPFKATI QPVFDPSKVR ASGPGLERGK AGEAATFTVD CSEAGEAELT IEILSDAGVK
1201 AEVLIQNNAD GTYHITYSPA FPGTYTITIK YGGHPIPKFP TRVHVQPAVD TSGIKVSGPG VEPHGVLREV TTEFTVDARS LTATGGNHVT ARVLNPSGAK
1301 TDTYVTDNGD GTYRVQYTAY EEGVHLVEVL YDEVAVPKSP FRVGVTEGCD PTRVRAFGPG LEGGLVNKAN RFTVETRGAG TGGLGLAIEG PSEAKMSCKD
1401 NKDGSCTVEY IPFTPGDYDV NITFGGQPIP GSPFRVPVKD VVDPGKVKCS GPGLGTGVRA RVPQTFTVDC SQAGRAPLQV AVLGPTGVAE PVEVRDNGDG
1501 THTVHYTPAT DGPYTVAVKY ADQEVPRSPF KIKVLPSHDA SKVRASGPGL NASGIPASLP VEFTIDARDA GQGLLTVQIL DPEGKPKKAN IRDNGDGTYT
1601 VSYLPDMSGR YTITIKYGGD EIPYSPFRIH ALPTGDASKC LVTVSIGGHG LGACLGPRIQ IGEETVITVD AKAAGKGKVT CTVSTPDGAE LDVDVVENHD
1701 GTFDIYYTAP EPGKYVITIR FGGEHIPNSP FHVLATEEPV VPVEPLESML RPFNLVIPFT VQKGELTGEV RMPSGKTARP NITDNKDGTI TVRYAPTEKG
1801 LHQMGIKYDG NHIPGSPLQF YVDAINSRHV SAYGPGLSHG MVNKPATFTI VTKDAGEGGL SLAVEGPSKA EITCKDNKDG TCTVSYLPTA PGDYSIIVRF
1901 DDKHIPGSPF TAKITGDDSM RTSQLNVGTS TDVSLKITEG DLSQLTASIR APSGNEEPCL LKRLPNRHIG ISFTPKEVGE HVVSVRKSGK HVTNSPFKIL
2001 VGPSEIGDAS KVRVWGKGLS EGQTFQVAEF IVDTRNAGYG GLGLSIEGPS KVDINCEDME DGTCKVTYCP TEPGTYIINI KFADKHVPGS PFTVKVTGEG
2101 RMKESITRRR QAPSIATIGS TCDLNLKIPG NWFQMVSAQE RLTRTFTRSS HTYTRTERTE ISKTRGGETK REVRVEESTQ VGGDPFPAVF GDFLGRERLG
2201 SFGSITRQQE GEASSQDMTA QVTSPSGKTE AAEIVEGEDS AYSVRFVPQE MGPHTVTVKY RGQHVPGSPF QFTVGPLGEG GAHKVRAGGT GLERGVAGVP
2301 AEFSIWTREA GAGGLSIAVE GPSKAEIAFE DRKDGSCGVS YVVQEPGDYE VSIKFNDEHI PDSPFVVPVA SLSDDARRLT VTSLQETGLK VNQPASFAVQ
2401 LNGARGVIDA RVHTPSGAVE ECYVSELDSD KHTIRFIPHE NGVHSIDVKF NGAHIPGSPF KIRVGEQSQA GDPGLVSAYG PGLEGGTTGV SSEFIVNTQN
2501 AGSGALSVTI DGPSKVQLDC RECPEGHVVT YTPMAPGNYL IAIKYGGPQH IVGSPFKAKV TGPRLSGGHS LHETSTVLVE TVTKSSSSRG ASYSSIPKFS
2601 SDASKVVTRG PGLSQAFVGQ KNSFTVDCSK AGTNMMMVGV HGPKTPCEEV YVKHMGNRVY NVTYTVKEKG DYILIVKWGD ESVPGSPFKV NVP
```

Supplementary Figure 8. Identification and validation of the interaction of FLNc and FILIP1. **a.** Hierarchical cluster analysis of the SILAC data obtained in the study of the protein nano-environment around hFLNc d18-21 by in vivo proximity labeling in skeletal myotubes. Mean log_{10} transformed SILAC ratios of quantified proteins were used (n=3 independent experiments). Only proteins in cluster 1 were deemed components of the hFLNc d18-21 interactome, since they were strongly enriched in comparison to both controls. Cluster 2 and 3 mainly contained unspecific binding partners and contaminants. Cluster 1, light red; cluster 2, light blue; cluster 3, gray. **b.** GO enrichment analysis. Shown are the cellular compartments containing proteins enriched in cluster 1. Z-disc and Z-disc-associated ontologies were overrepresented in cluster 1. **c.** Schematic illustration of FILIP1 isoforms. Shown are exons and slicing events. The isoforms FILIP1-1 and FILIP1-2 represent the Uniprot entries Q7Z7B0-1 and Q7Z7B0-2, respectively. The isoform FILIP1-4 lacks exon 6 as a result of alternative splicing. **d.** Sequence alignment of the carboxy-termini of FILIP1 isoforms 1 to 4 using Clustal Omega. **e.** Immunoblot analysis using an antiserum directed against its carboxy-terminus shows expression of FILIP1 (~130 kDa) in mouse skeletal muscle (lane 1) and cultured C2C12 myotubes (lane 3). The specificity of the antiserum was shown by adding the antigen (T7-tagged FILIP1-2 CT) to the antibody solution ("block", lanes 2 and 4). Binding to FILIP1 was abolished, but a distinct band above the 180 kDa marker band appeared, indicating binding of the antibody/antigen complex to another protein in the extracts. The presence of FILIP1-2 CT was confirmed using T7-tag antibody (lane 5), whereas binding of the antibody/antigen complex to endogenous FLNc was confirmed using anti-FLNc antibody (lane 6). **f.** Yeast-two hybrid analysis indicates binding of FILIP1-4 to FLNc d16-19, d17-19, d18-21 and d20-21 in yeast cells. -: no binding; +: weak binding; ++: moderate binding; +++: strong binding **g.** Coomassie-stained gel from FLNc d1-3 and FLNc d18-21 pulldown with myotube lysates. Bands of recombinant FLNc d1-3 (*) and recombinant FLNc d18-21 (#) are marked. For identification of FILIP a gel band at approx. 130 kDa was cut from each lane as indicated and analyzed by LC-MS. FILIP1 was unambigously identified in the pulldown with FLNc d18-21, but not FLNc d1-3 as illustrated by the respective LFQ-based intensity bar chart. Myh8, Myosin-8 **h.** Identification of endogenous FLNc in FILIP1 CT pull-down by LC-MS analysis. FLNc was identified with a sequence coverage of 78%. Identified peptides are shown in red.

Supplementary Figure 9

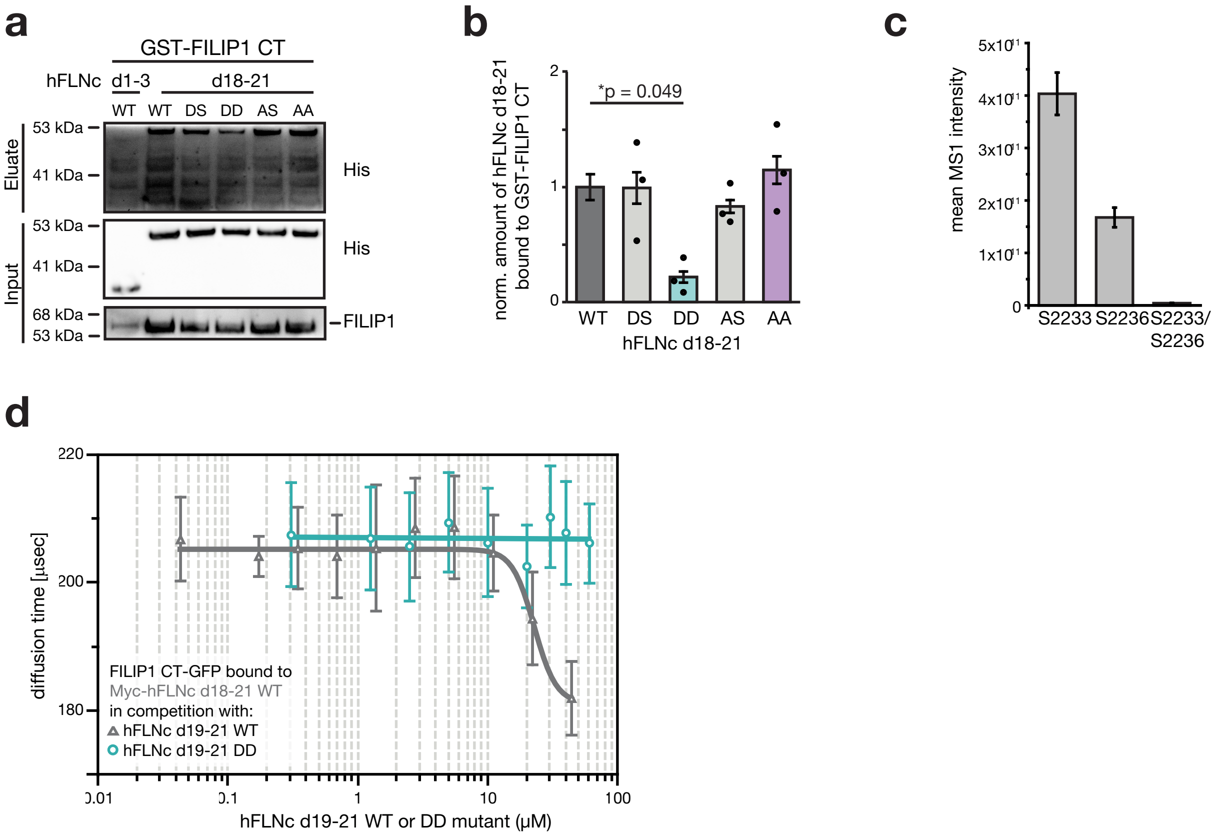

Supplementary Figure 9. Analysis of FILIP1 binding to human (h) FLNc in dependency of S2233/S2236 phosphorylation. **a.** Pull-down experiments using GST-FILIP1 CT as bait and His_6-tagged hFLNc d18-21 wild-type or S2233/S2236 phosphosite mutants as prey as indicated. Wild-type hFLNc d1-3 served as negative control. WT, wild-type; CT, carboxy-terminus; d, domain. **b.** Quantification of immunoblot data shown in (a). Calculated signal intensities were normalized to hFLNc d18-21. SEMs were calculated and a two-tailed student's t-test was performed (n = 3 independent experiments; *p ≤ 0.05). **c.** Mean MS intensities of phosphopeptides derived from recombinantly expressed hFLNc d18-21 in HEK293T cells. Peptides phosphorylated at S2233, S2236 or both 2233 and S2236 were summed and for each the mean MS1 intensity is shown (n=3 independent experiments, ± SEM). **d.** Fluorescence correlation spectroscopy analysis of co-expressed FILIP CT-GFP and Myc-hFLNc d18-21 in competition with recombinantly expressed hFLNc d19-21 WT (Kd = 22.85 + 2.86 µM) and hFLNc d19-21 DD mutant (Kd = n.a.), respectively. Data of 3 independent experiments are shown.

Supplementary Figure 10

area in boxes shown in Fig. 6f

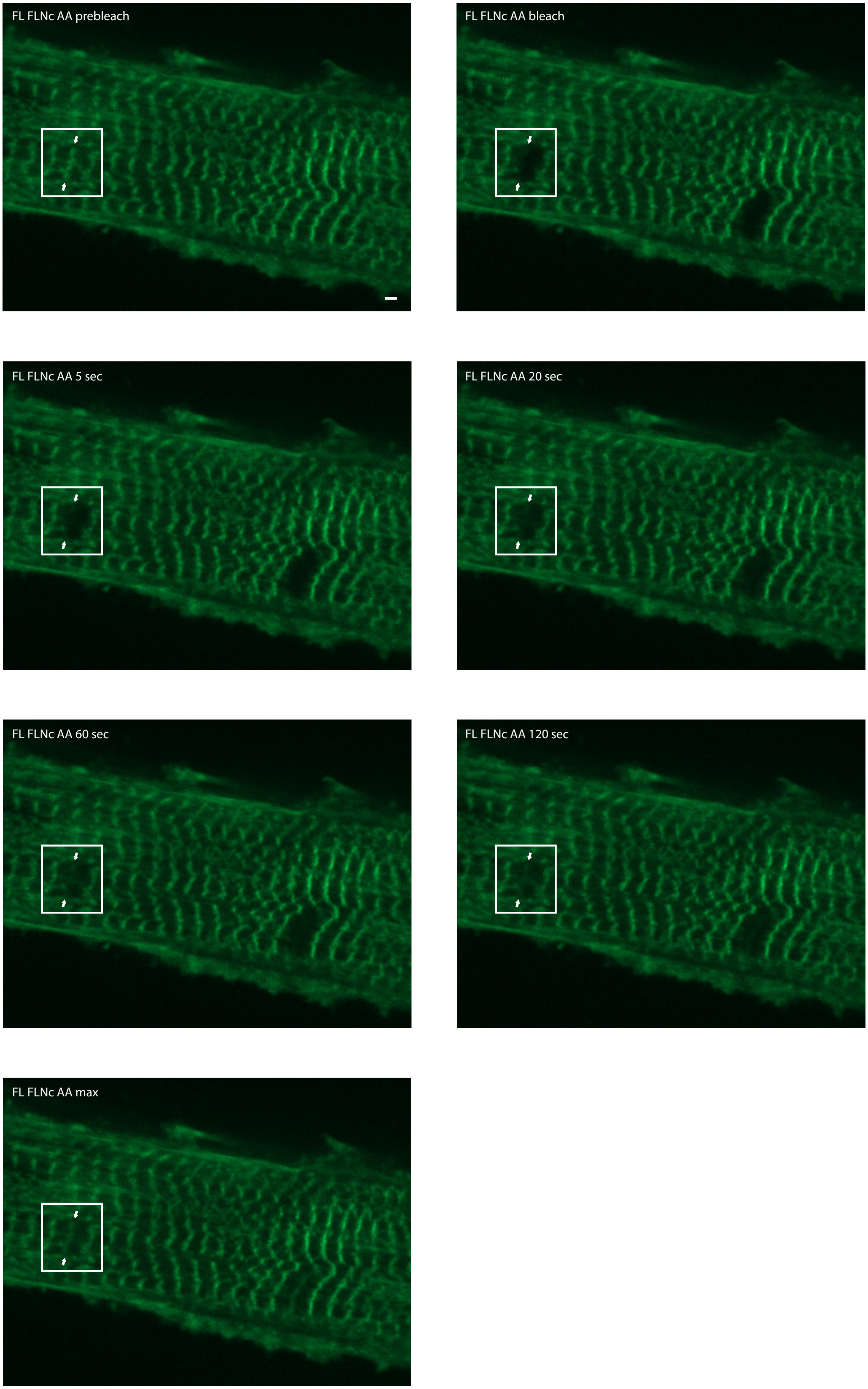

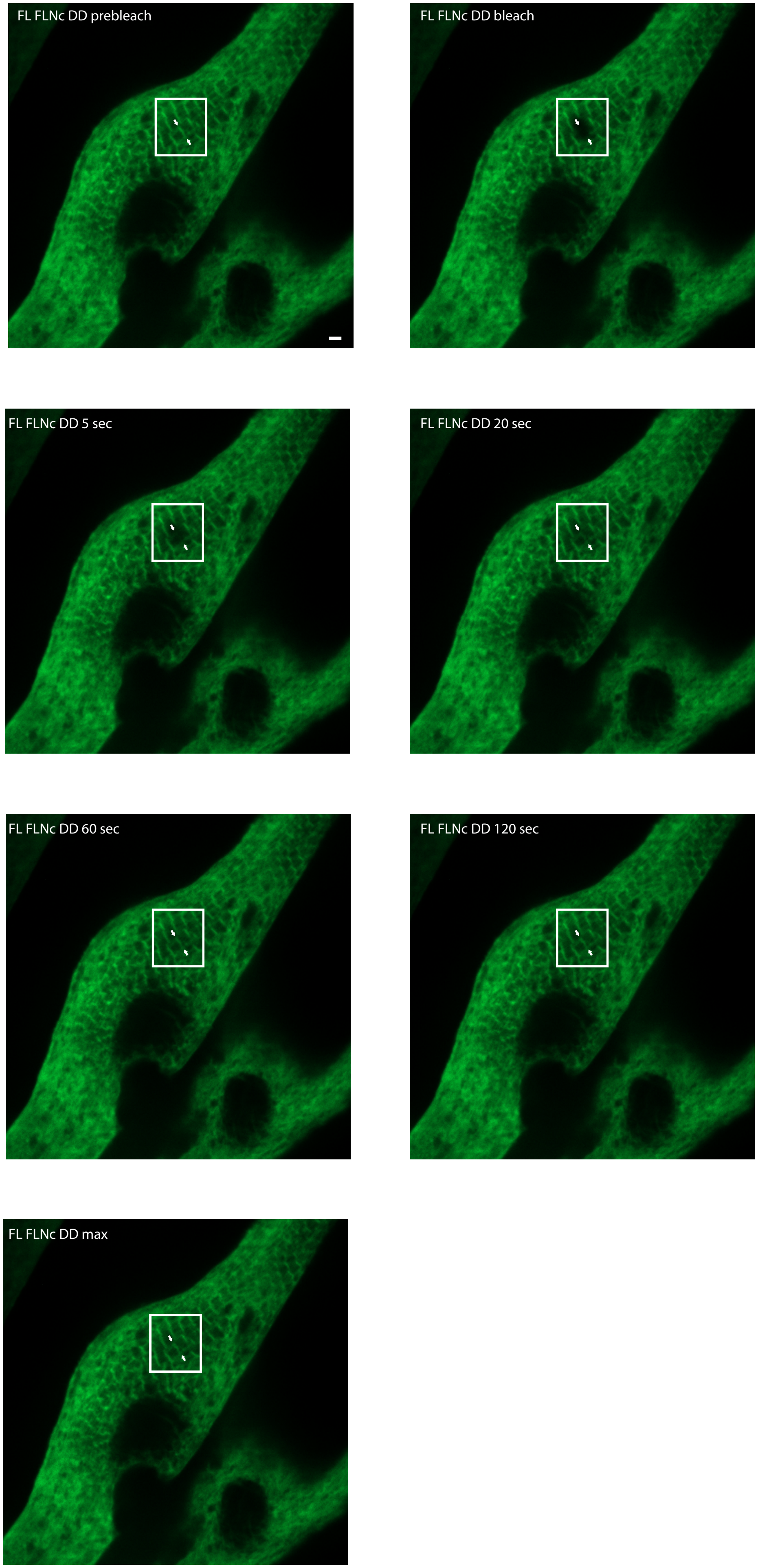
FL FLNc DD prebleach
FL FLNc DD bleach
FL FLNc DD 5 sec
FL FLNc DD 20 sec
FL FLNc DD 60 sec
FL FLNc DD 120 sec
FL FLNc DD max

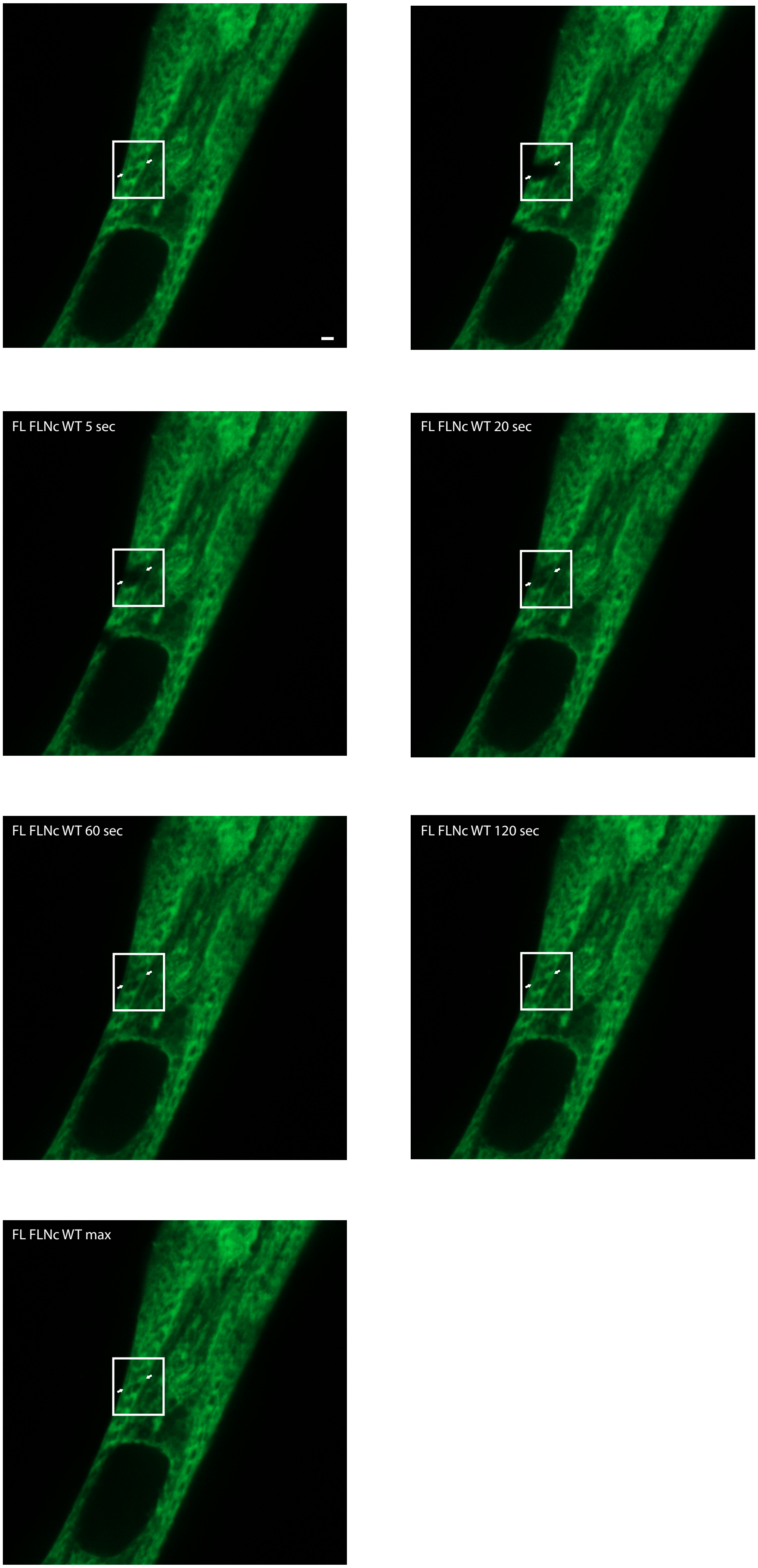

Supplementary Figure 10. Uncropped microscopic data from fluorescence recovery after photobleaching (FRAP) experiments, shown in Fig. 6f.

Supplementary Figure 11

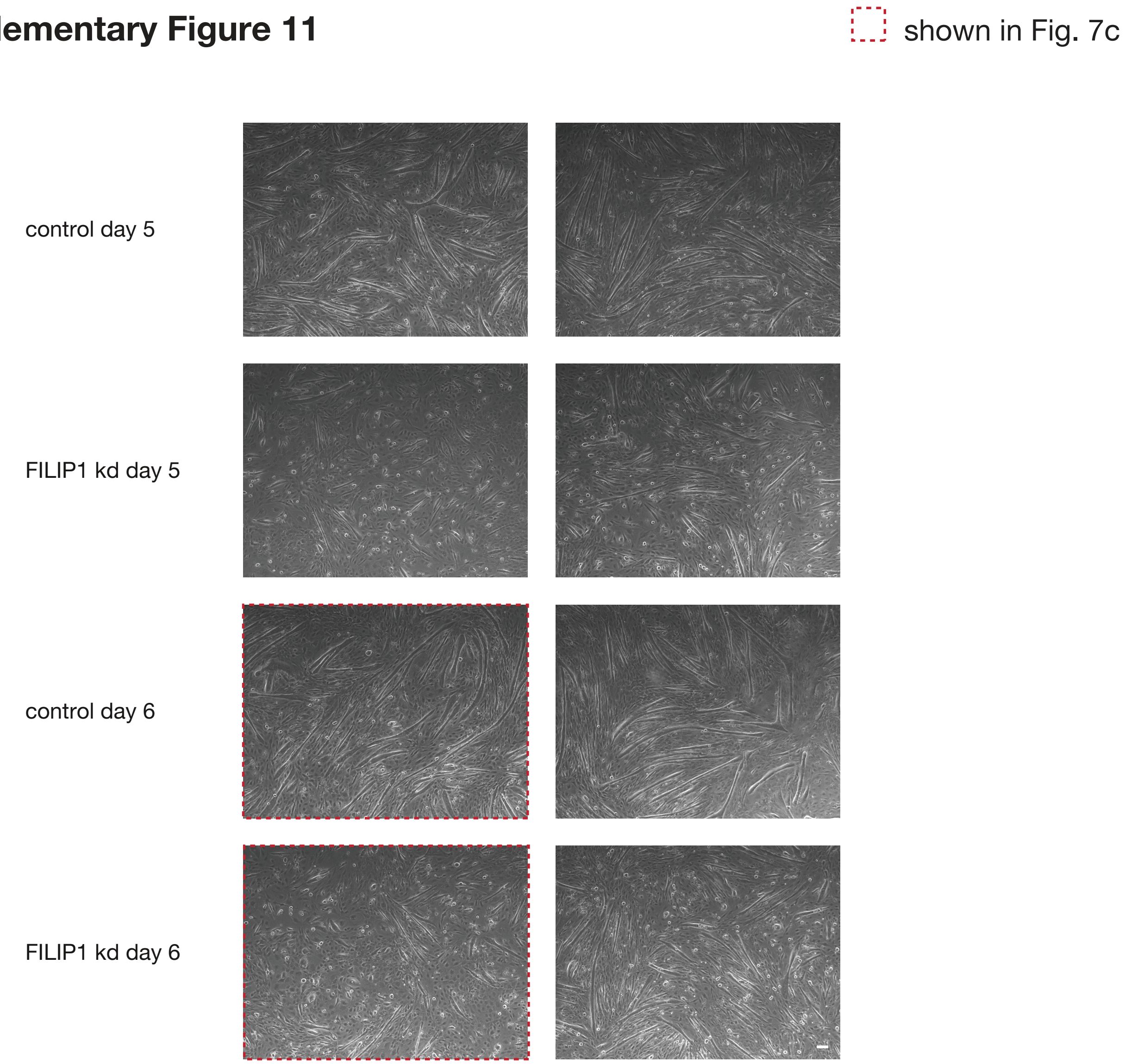

Supplementary Figure 11. Uncropped bright-field microscopy images from FILIP1 knockdown or control C2C12 cells, shown in Fig. 7c.

Supplementary Figure 12 area in boxes shown in Fig. 7d

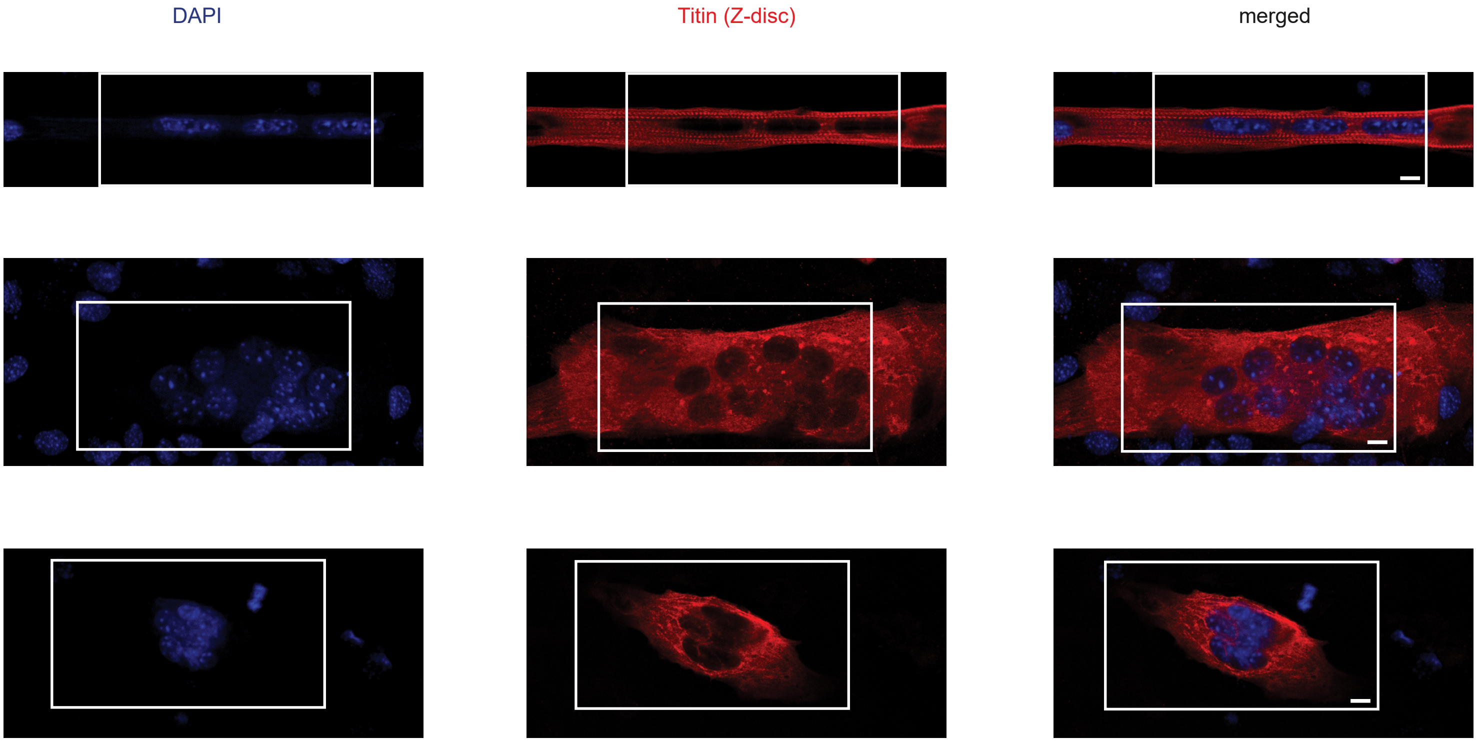

Supplementary Figure 12. Uncropped fluorescence microscopy images of FILIP1 knockdown or control C2C12 cells, shown in Fig. 7d.

Supplementary Figure 13

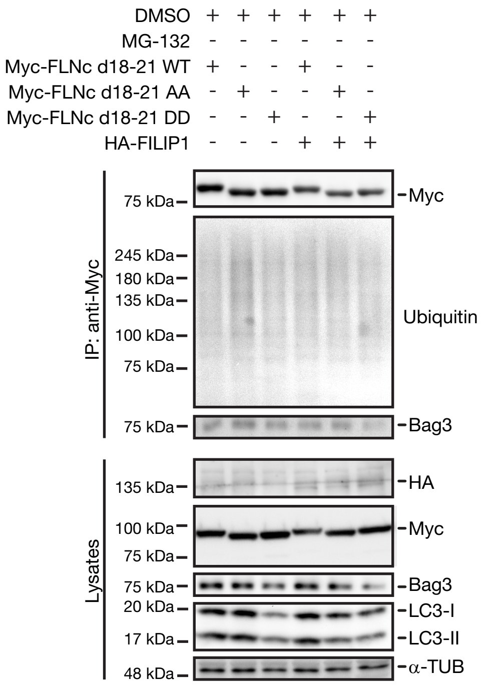

Supplementary Figure 13: Immunoblot analysis of control samples performed for experiment shown in Figure 7h. C2 cells were transfected with FLNc d18-21 WT or mutant forms in the presence or absence of HA-FILIP1. Cells were treated 6 h with DMSO before lysis and anti-Myc IP was performed. The first lane of the immunoblot corresponds to the last lane of the immunoblot shown in Figure 7h.
