# Supplementary material for: Phosphoproteomics identifies dual-site phosphorylation in an extended basophilic motif regulating FILIP1-mediated degradation of filamin-C
Source: Commun Biol. 2020 May 22;3:253. doi: 10.1038/s42003-020-0982-5 (PMC7244511; doi:10.1038/s42003-020-0982-5)
Supplement: Supplementary file 15 — Description of Additional Supplementary Files [file 42003_2020_982_MOESM15_ESM.pdf]

SUPPLEMENTARY DATA LEGENDS

Supplementary Data 1. Proteome dataset. Excerpts from the MaxQuant output files "proteinGroups" (spreadsheet proteins) and "peptides.txt" (spreadsheet peptides) derived from the quantitative proteome analysis of contracting C2 myotubes; contains information about the identification of proteins/protein groups and peptides. Contaminants and reversed entries were removed from the lists.

Supplementary Data 2. Global IGF-1/LY phosphoproteome dataset. Excerpts from the MaxQuant output file "PhosphoSTY" derived from the quantitative phosphoproteome analysis of contracting C2 myotubes treated for 60 min with IGF-1 or LY294002; contains information about phosphosite identification and localization as well as log_2-transformed mean SILAC ratios (treatment/control) after p-value correction. Phosphopeptides with a minimum fold change of 1.5 and an adjusted p-value < 0.05 (n=3; two-tailed moderated student's t-test) are marked as significant. Contaminants and reversed entries were removed from the lists.

Supplementary Data 3. List of unique phosphopeptides used for the motif analysis shown in Fig. 2c and 2d and Fig. S3c and S3d and motif-X results.

Supplementary Data 4. Results of GO enrichment analyses for phosphoproteins comprising the overrepresented sequence motifs.

Supplementary Data 5. Text mining results.

Supplementary Data 6. Global LY/MK phosphoproteome dataset. Excerpts from the MaxQuant output file "PhosphoSTY" derived from the quantitative phosphoproteome analysis of contracting C2 myotubes treated form 30 min with IGF-1, LY294002 + IGF-1 and MK-2206 + IGF1; contains information about phosphosite identification and localization as well as log_2-transformed mean SILAC ratios (treatment/control) after p-value correction. Phosphopeptides with a minimum fold change of 1.5 and an adjusted p-value < 0.01 (n=6; two-tailed moderated student's t-test) are marked as significant. Contaminants and reversed entries were removed from the lists. Table also contains data from Kinase-substrate enrichment analysis).

Supplementary Data 7. Results from hierarchical cluster analysis and Reactome analysis with significantly regulated phosphopeptides from IGF-1/LY and LY/MK phosphoproteomes. Results from (i) first clustering refer to Supplementary Fig. 3e, (ii) second clustering to Fig. 3c and 3e, and (iii) Reactome analysis to Fig. 3d.

Supplementary Data 8. Quantitative MS data from *in vitro* kinase assays using Akt, PKCα and hFLNc d18-21. Label-free quantification was performed using the Skyline software. Phosphopeptides from each of the three independent experiments were analyzed by LC-MS using multi-stage activation (MSA), electron-transfer dissociation (ETD) and higher-energy collisional dissociation (HCD).

Supplementary Data 9. Quantitative MS data from cell-based kinase assays exported from Skyline. Label-free quantification was performed using the Skyline software. Phosphopeptides from each of the four independent experiments were analyzed by targeted LC-MS using parallel reaction monitoring and higher-energy collisional dissociation (HCD).

Supplementary Data 10. SILAC-MS data from *in vivo* proximity labelling experiments using BirA*hFLNc d18-21 in C2 myotubes. Excerpts from the MaxQuant output files "proteinGroups", including information about identified proteins/protein groups as well as the assigned cluster from k-means cluster analysis. Contaminants and reversed entries were removed from the lists.

Supplementary Data 11. Result files from GO enrichment analysis of the BioID dataset after cluster analysis. Enrichment analysis was performed with the Cytoscape 3.3.0 app ClueGO. Enrichment was analyzed for the GO domain cellular component (CC). Bonferroni-step-down false discovery rate correction at a significance level of 0.05 was used for p-value correction of each cluster after the hypergeometric statistic test against all identified proteins within the analysis.

Supplementary Data 12. Excerpts from the MaxQuant 'ProteinGroups' output file for the MS analysis of FLNc d1-3 and d18-21 pull-down experiments. This file contains two spreadsheets with the first one (column header) giving explanations on the column headers used in the spreadsheet "MaxQuant results FLNc pull-down".

Supplementary Data 13. Excerpts from the Mascot output file following MS analysis of FILIP1 pull-down experiment. This file contains two spreadsheets with the first one (column header) giving explanations on the column header used in the spreadsheet "Mascot results FILIP pulldown".
