# Supplementary material for: Phosphoproteomics identifies dual-site phosphorylation in an extended basophilic motif regulating FILIP1-mediated degradation of filamin-C
Source: Commun Biol. 2020 May 22;3:253. doi: 10.1038/s42003-020-0982-5 (PMC7244511; doi:10.1038/s42003-020-0982-5)
Supplement: Supplementary file 17 — Peer Review File [file 42003_2020_982_MOESM17_ESM.pdf]

Editorial Note: *This manuscript has been previously reviewed at another Nature Research journal. The manuscript was considered suitable for publication without further review at Communications Biology*
